# Conditional Activation of Protein Therapeutics by Templated Removal of Peptide Nucleic Acid Masking Groups

**DOI:** 10.1002/anie.202502268

**Published:** 2025-03-17

**Authors:** Bengt H. Gless, Elisabeth Jones, Carlos Labão‐Almeida, Cong Tang, Nicole Gottscheber, Renata Couto, Gonçalo J. L. Bernardes

**Affiliations:** ^1^ Yusuf Hamied Department of Chemistry University of Cambridge Lensfield Road 2 Cambridge CB2 1EW UK; ^2^ GiMM ‐ Gulbenkian Institute for Molecular Medicine Avenida Prof. Egas Moniz Lisboa 1649‐035 Portugal; ^3^ Translational Chemical Biology Group Spanish National Cancer Research Centre (CNIO) Madrid 28029 Spain

**Keywords:** Conditional activation, Cytokines, Immunotherapy, Peptide nucleic acids, Protein masking

## Abstract

Interleukin‐2 (IL‐2)‐based therapeutics are emerging as treatments for immunotherapy; however, systemic activation of immune cells hampers their success. Chemically controlling the activity of potent cytokines could mitigate unwanted T cell stimulation and widen their therapeutic window. In this study, we developed a strategy for the conditional activation of proteins utilizing removable peptide nucleic acid (PNA) masking groups. Site‐specific installation of “Lock”‐PNAs containing a cleavage thioester linkage enabled steric blockage of receptor binding sites. Rapid unmasking and activation were performed by the addition of a complementary “Key”‐PNA containing a cysteine (Cys) residue, which forms a PNA–PNA duplex leading to a proximity‐accelerated cleavage step and release of the active protein. We exemplified the versatility of this methodology on de novo cytokine neoleukin‐2/15 (Neo‐2/15) through the preparation of PNA conjugates including homodimers, PNA‐stapled conjugates, and dual PNA‐bridged dimers. All constructs were effectively unmasked at low micromolar concentrations. Further, we demonstrated the conditional activation of a masked conjugate of Neo‐2/15 in binding studies to the IL‐2 receptors and in an ex vivo T cell signaling assay displaying a 480‐fold potency increase upon activation. Finally, we extended the strategy to a designed ankyrin repeat protein (DARPin) activating the human CD40 receptor demonstrating successful masking and unmasking.

## Graphical abstract

A strategy for the conditional activation of therapeutic proteins utilizing removable peptide nucleic acid (PNA) masking groups is presented. Site‐specific installation of “Lock”‐PNAs containing a cleavage thioester linkage enabled steric blockage of receptor binding sites. On‐demand unmasking induced by a complementary “Key”‐PNA containing a cysteine residue allowed the controlled release of active proteins.

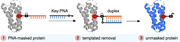

## Introduction

Protein therapeutics play a major role in cancer therapy with immuno‐oncology emerging as a cornerstone for biologics following the success of immune checkpoint blockers^[^ ^1^, ^2^ ^]^ and T cell‐engaging bispecific antibodies.^[^ ^3^, ^4^ ^]^ Cytokine therapeutics, particularly strategies involving IL‐2, have experienced notable advancements in the past decade with multiple candidates currently under evaluation in clinical trials.^[^ ^5^ ^]^ IL‐2 is an important signaling protein in T cell regulation and IL‐2‐based therapies rely on the activation of T cells and natural killer (NK) cells in the tumor tissue to evoke an antitumoral immune response.^[^ ^6^, ^7^ ^]^ However, the systemic stimulation of T cells and resulting adverse effects are a key challenge in the development of successful IL‐2‐based therapy.^[^ ^8^ ^]^ Numerous strategies have been developed to address the shortcomings of IL‐2 to broaden the therapeutic window and enable higher dosing.^[^ ^8^, ^9^, ^10^, ^11^, ^12^ ^]^

**Figure 1:**
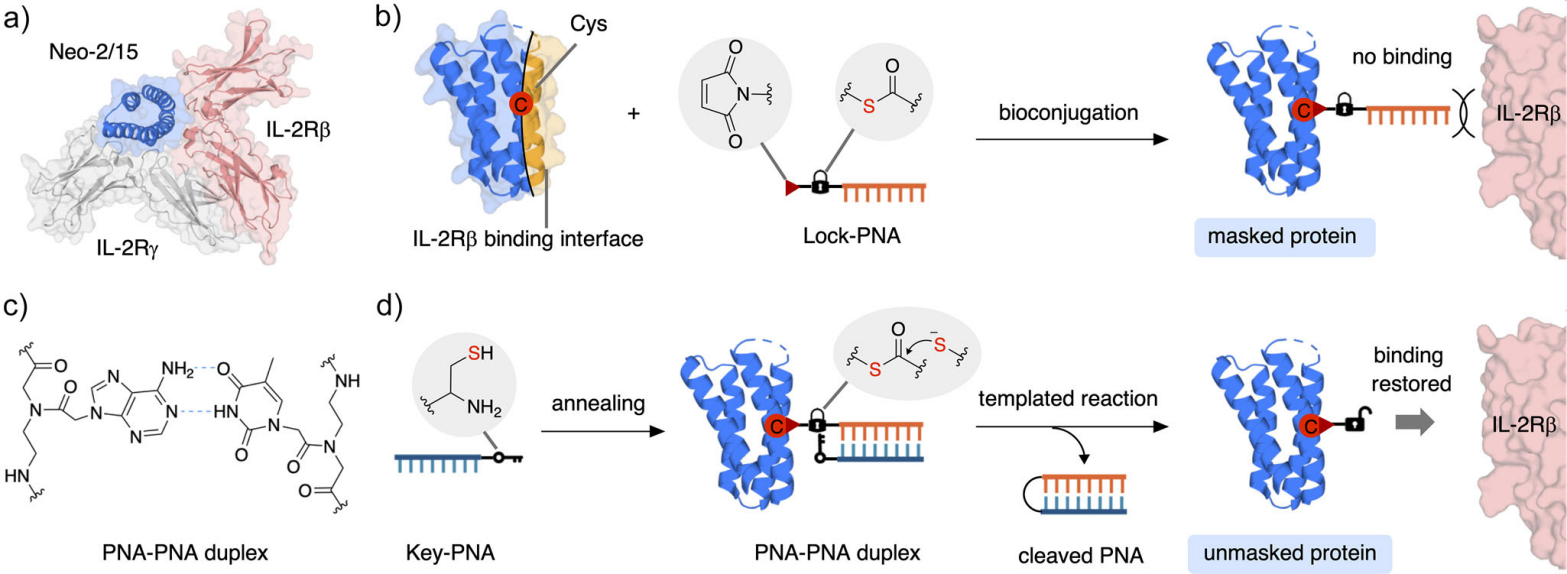
Concept of PNA‐based protein masking for Neo‐2/15. a) Crystal structure of Neo‐2/15 bound to IL‐2Rβ and IL‐2Rγ (PDB: 6DG5). b) Bioconjugation of a maleimide and thioester‐containing Lock‐PNA to a mutant of Neo‐2/15 with a Cys residue in the periphery of the IL‐2Rβ binding interface creates a masked PNA‐protein conjugate. c) Structure of a PNA–PNA duplex. d) Addition of a complementary Key‐PNA containing a C‐terminal Cys residue leads to the formation of PNA–PNA duplex and introduces proximity between the Cys residue and the thioester. The following templated transthioesterification results in the removal of the PNA–PNA duplex and releases a unmasked Neo‐2/15 modified with a trace molecule from the bioconjugation handle.

Alterations to the receptor binding properties^[^ ^13^, ^14^, ^15^ ^]^ and antibody–IL‐2 fusion proteins, so called immunocytokines,^[^ ^16^, ^17^, ^18^, ^19^ ^]^ have been applied to reduce unfavorable T cell activation. In addition, protein masking strategies^[^ ^20^ ^]^ for IL‐2 have emerged to mitigate its picomolar activities by prohibiting receptor binding until a conditionally triggered event.^[^ ^12^ ^]^ Triggers specific to the tumor microenvironment (TME) include pH change,^[^ ^21^ ^]^ enzymatic removal of masking domains through TME abundant proteases,^[^ ^22^, ^23^ ^]^ and binding to a tumor‐associated antigen (TAA) enabling IL‐2 signaling through conformational changes.^[^ ^24^ ^]^ Another promising approach is to computationally design de novo cytokines with improved therapeutic properties.^[^ ^25^, ^26^, ^27^, ^28^ ^]^ In particular, a mimic of IL‐2, called neoleukin‐2/15 (Neo‐2/15),^[^ ^25^ ^]^ received attention and was the first de novo protein in clinical trials. The protein exclusively binds the IL‐2 receptor chain beta (IL‐2Rβ, CD122) and chain gamma (IL‐2Rγ, CD132) with very high affinity but does not bind the IL‐2 receptor chain alpha (IL‐2Rα, CD25) (Figure 1a). As a result, it has no bias for immunosuppressive regulatory T cells (Treg) over CD8^+^ T cell and NK cells and exhibits picomolar potency for T cell activation. Despite promising early clinical development,^[^ ^29^ ^]^ phase 1 clinical trials of a PEGylated Neo‐2/15 conjugate were discontinued due to a lack of efficacy.^[^ ^30^ ^]^

Our aim was to develop a proximity‐based masking methodology for cytokine therapeutics that enables the conditional activation upon addition of a trigger molecule, and we identified Neo‐2/15 as well‐suited proof‐of‐concept target protein due to its therapeutic potential and its excellent stability facilitating chemical modifications. Herein, we report a strategy for the conditional activation of therapeutic proteins, exemplified on Neo‐2/15, by site‐specific installation of removable masking groups consisting of PNAs linked via a cleavable linker (Figure 1b).

## Results and Discussion

### Development of PNA Masking Group

PNAs are non‐charged mimics of nucleic acids with a peptide‐like backbone that follow Watson–Crick base pairing rules and anneal with significantly higher melting temperatures then native nucleic acids (Figure 1c).^[^ ^31^, ^32^ ^]^ The peptidic nature of PNAs renders them suitable for protein bioconjugation^[^ ^33^, ^34^, ^35^ ^]^ and manipulating protein activities,^[^ ^36^, ^37^, ^38^ ^]^ as well as facilitating their preparation through the use of standard Fmoc‐based peptide chemistry.^[^ ^39^ ^]^

We anticipated that PNAs could function as removable masking groups on proteins. We envisioned to install a “Lock”‐PNA containing a bioconjugation handle and cleavable linker onto a Cys residue located in the periphery of the IL‐2Rβ binding interface of a Neo‐2/15 mutant. The masked PNA‐protein conjugate would be unable to bind to IL‐2Rβ due to steric hindrance and be biologically inactive (Figure 1b).

The chemistry for the templated removal reaction had to fulfill certain requirements. It should have slow reactions rates that can be accelerated in proximity, proceed under physiological conditions, and be compatible with the synthesis of PNAs and bioconjugation reactions. We, therefore, chose the chemoselective reaction between a thioester and a N‐terminal Cys at neutral pH in aqueous medium, called native chemical ligation (NCL).^[^ ^40^ ^]^ Standard NCL reactions are used for the ligation of two peptide segments at millimolar concentrations and require acceleration through the addition of aryl thiol catalysts.^[^ ^41^ ^]^ However, the reaction of alkyl thioesters with Cys represents a significantly slower reaction^[^ ^42^, ^43^, ^44^ ^]^ that has been exploited for PNA‐templated NCL reactions in numerous examples and applications.^[^ ^45^, ^46^, ^47^, ^48^, ^49^, ^50^, ^51^ ^]^ Although most PNA‐based NCL reactions take place between two PNA segments on a longer nucleic acid template strand, thioester–thiol exchange reactions between two directly annealing strands have been shown for DNA duplexes.^[^ ^52^ ^]^ The thioester linkage has good hydrolytic stability^[^ ^43^, ^44^ ^]^ and thiol–thioester exchange is slow at the low concentrations of small molecule thiols in serum,^[^ ^53^ ^]^ which could provide sufficient stability of the masked conjugates in vivo. Nevertheless, a potential challenge might arise from serum esterases.

**Figure 2:**
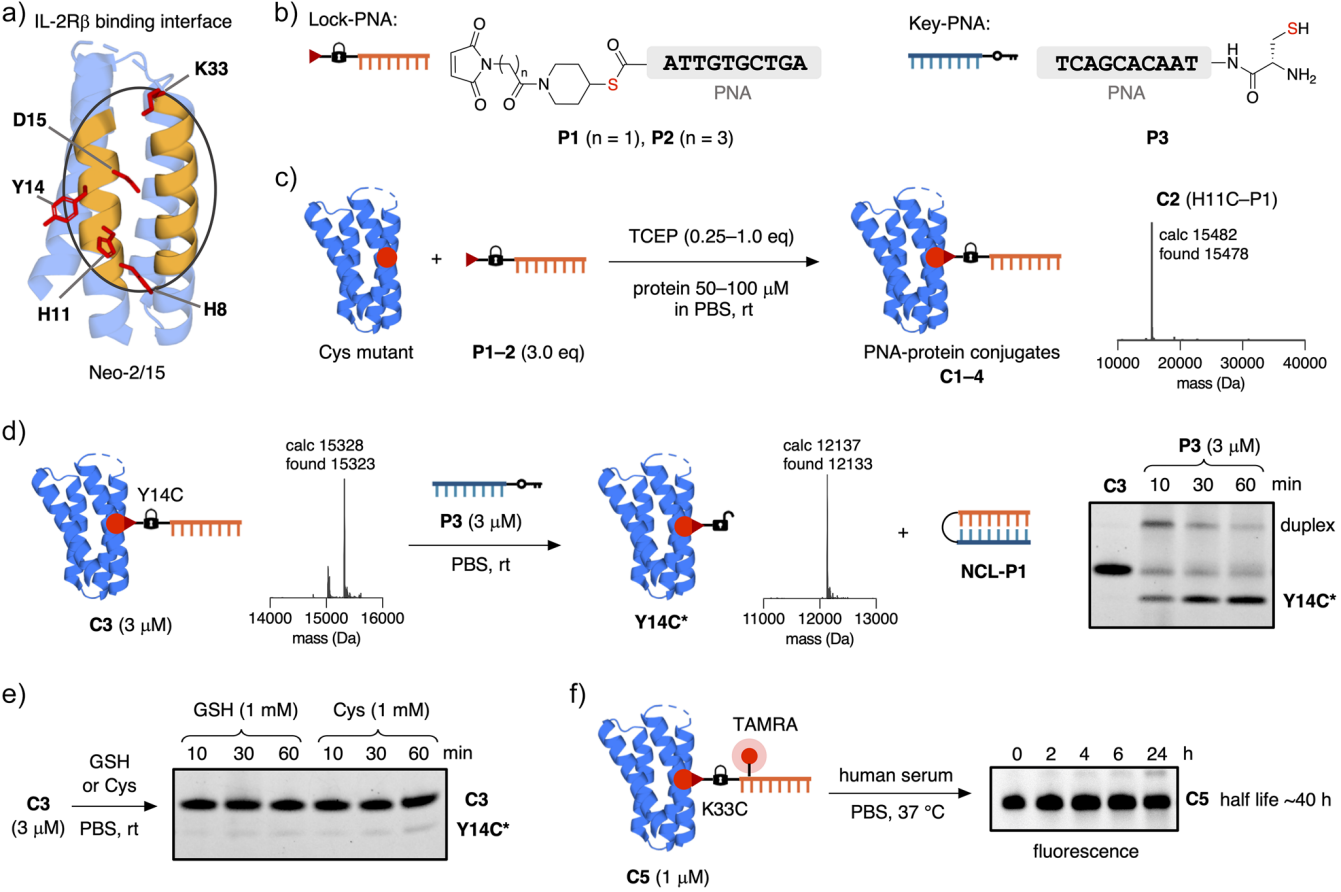
Single Cys PNA‐Neo‐2/15 conjugates. a) Expressed single Cys mutants of Neo‐2/15. b) Simplified structures of thioester‐containing Lock‐PNAs **P1** and **P2** and Cys‐containing Key‐PNA **P3**. c) Bioconjugation of Cys mutants of Neo‐2/15 and Lock‐PNAs (**P1**, **P2**) in the presence of tris(2‐carboxyethyl)phosphine (TCEP). Representative mass spectrum of purified **C2**. d) Templated unmasking reaction of conjugate **C3** with Key‐PNA **P3** released trace‐modified protein Y14C* and covalent PNA–PNA duplex **NCL‐P1**. e) Stability of conjugate **C3** in 1 mM solutions of GSH and Cys. f) Stability of TAMRA‐labelled conjugate **C5** in human serum–phosphate‐buffered saline (PBS) (1:1). All conjugates were purified by size exclusion chromatography (SEC). Sodium dodecyl sulfate polyacrylamide gel electrophoresis (SDS‐PAGE) was performed without preheating under non‐reducing conditions.

**Figure 3:**
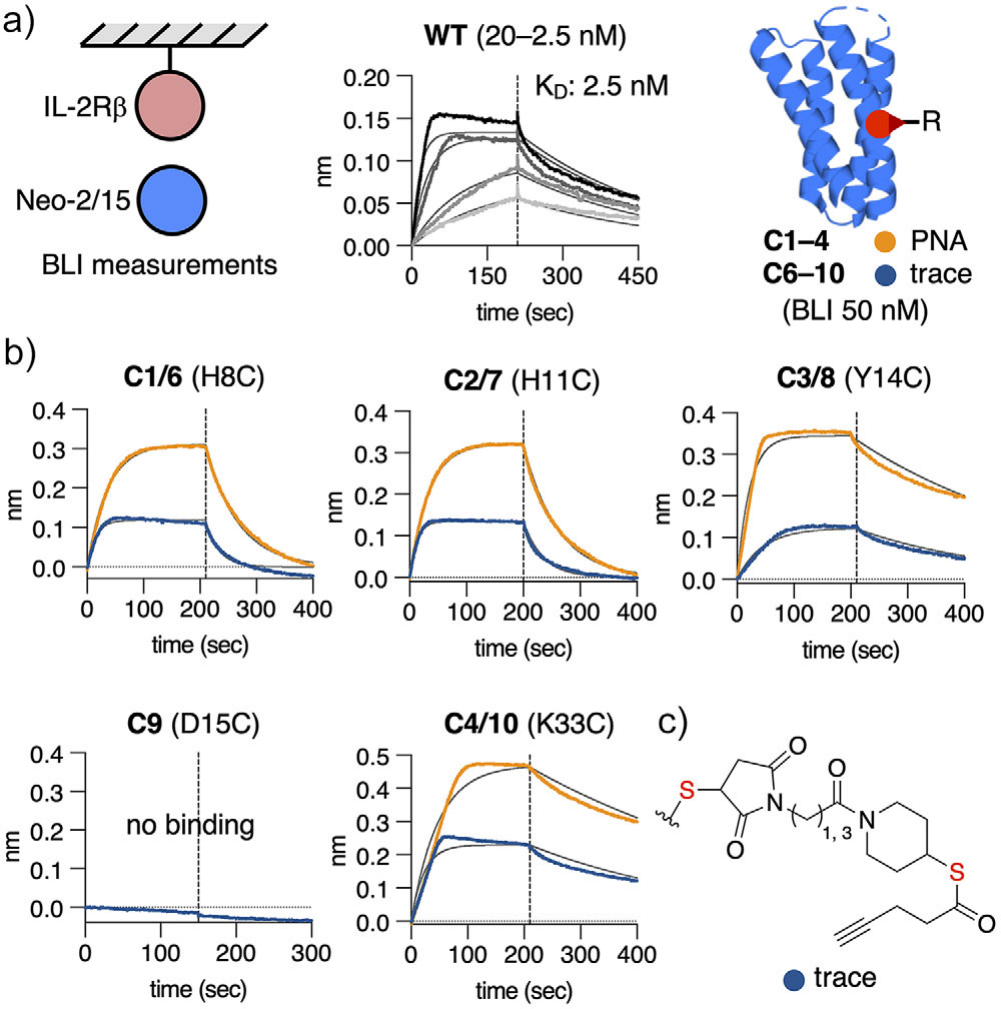
BLI measurements of PNA‐Neo‐2/15 conjugates. a) BLI measurement were performed with immobilized human IL‐2Rβ. Neo‐2/15 **WT** was tested at concentration range (20–2.5 nM) and conjugates of Neo‐2/15 were tested at 50 nM. b) Binding curves of PNA‐protein (**C1**–**C4**) and linker‐protein conjugates (**C6**–**C10**). c) Structure of attached maleimide thioester linker as mimic of remaining trace molecule after demasking.

As the first step, we examined the crystal structure of Neo‐2/15 in complex with mouse IL‐2Rβ/IL‐2Rγ and identified 4 residues (H8, H11, Y14, and K33) in the periphery of the IL‐2Rβ interface as well as the residue D15, which is equivalent to D20 in IL‐2,^[^ ^54^ ^]^ a residue that is essential for the IL‐2/IL‐2Rβ interaction (Figures 2a and S1). The corresponding single Cys mutants of Neo‐2/15 (**H8C**, **H11C**, **Y14C**, **D15C**, **K33C**) were expressed using a standard *E. coli* expression system and obtained in good yields. Next, we prepared Lock‐PNAs (**P1**, **P2**) with 10 nucleotides (nt) in length through on‐resin copper‐catalyzed azide–alkyne cycloaddition (CuAAC) using maleimide thioester linkers and a complementary Key‐PNA containing a Cys residue (**P3**) (Figures 2b and S2, for synthetic details see Schemes S1 and S2). With the PNAs and proteins in hand, we performed bioconjugation reactions under slightly reducing conditions and obtained the PNA‐protein conjugates **C1**–**C4** of the four Cys mutants peripheral to the binding site (Figure 2c). The bioconjugation reactions proceeded smoothly, and as expected, no transthioesterification between the thioester linker and the proteins was observed due to the difference in reaction rates of maleimides and alkyl thioesters toward Cys.^[^ ^42^ ^]^ As the next step, we attempted the templated unmasking reaction between conjugate **C3** (Y14C–P2) and Key‐PNA **P3** (Figure 2d). We were pleased to find that the unmasking NCL reaction afforded trace‐modified Y14C* within 10 min at room temperature using equimolar amounts of **P3** and showed nearly full release in 30 min (Figure 2d). In comparison, when we incubated **C3** in 1 mM solutions of glutathione (GSH) and Cys only minimal release was observed, highlighting the rate acceleration of PNA‐templated NCL (Figure 2e). Further unmasking experiments at 37 °C showed efficient release in 10 min and preincubating Key‐PNA **P3** with *N*‐methylmaleimide (NMM) fully prevented the unmasking step (Figure S2). Insufficient stability of PNA–thioester–protein conjugates in blood could represent an issue for their use in serum‐containing cell medium or in vivo. Therefore, we prepared fluorescently‐labelled conjugates that enabled us to test their serum stability (Figures 2f and S3, for synthetic details see Scheme S3). We incubated the fluorescent conjugate **C5**, which has tetramethylrhodamine (TAMRA) attached to the Lock‐PNA, in 50% human serum at 37 °C and estimated half‐life time of approx. 40 h under the tested condtions (Figure 2f). A conjugate where the fluorophore was linked directly to the protein via the thioester linker without the PNA resulted in a shorter half‐life time of approximately 8 h, indicating differences in enzymatic thioester cleavage rates dependant on its enviroment (Figure S3). The estimated half‐life of **C5** should provide sufficient stability to conduct cell‐based experiments and potential in vivo studies as it exceeds the renal clearance rate of small proteins in circulation.^[^ ^55^ ^]^ Having assessed the efficiency of the unmasking reaction and thioester stability, we determined the binding properties of the conjugates to IL‐2Rβ using biolayer interferometry (BLI) (Figures 3 and S4).

First, we measured the binding constant for expressed Neo‐2/15 **WT** (*K*_D_ = 2.5 nM), which was in good agreement with the reported value^[^ ^25^ ^]^ (Figure 3a) and we prepared conjugates of the all single Cys mutants, including **D15C**, with maleimide thioester linkers (**C6**–**C10**) to mimic the trace molecule that remains attached to the proteins after release (Figure 3c). In order to screen all conjugates for a binding/nonbinding assessment, we chose to perform single concentration measurements at 50 nM, approximately 20 times the *K*_D_ of **WT** (Figure 3b). The linker‐Neo‐2/15 conjugates of Cys mutations peripheral to the IL‐2Rβ interface displayed efficient binding to the receptor, with **C6** (H8C) and **C7** (H11C) having faster off‐rates than **WT**. **C9** (D15C) did not show any response, confirming the expected importance of D15 to the binding interaction with the receptor. Disappointingly, all PNA‐Neo‐2/15 conjugates **C1**–**C4** displayed rapid response curves representative of efficient binding to IL‐2Rβ only with higher absolute values (Figure 3b). In conclusion, discovering single residues suitable for Cys mutation and PNA masking proved to be challenging as the residue has to be close the binding interaction but cannot be essential to the binding.

### PNA‐Based Protein Stapling

**Figure 4:**
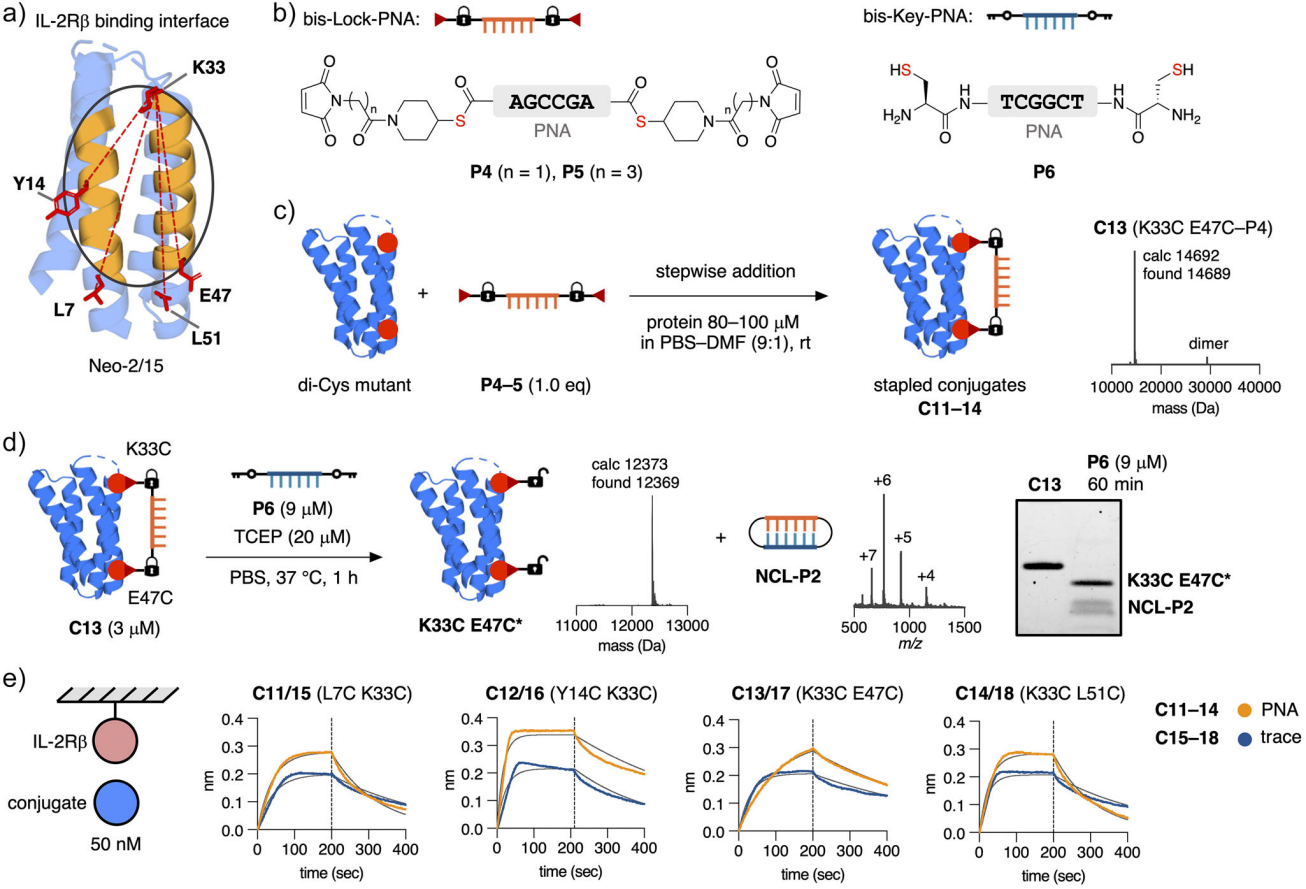
Double Cys PNA‐Neo‐2/15 conjugates. a) Expressed double Cys mutants of Neo‐2/15. b) Simplified structures of thioester‐containing bis‐Lock‐PNAs **P4** and **P5** and Cys‐containing bis‐Key‐PNA **P6**. c) Bioconjugation of double Cys mutants of Neo‐2/15 and bis‐Lock‐PNAs (**P4**, **P5**) via stepwise addition. Representative mass spectrum of purified **C13**. d) Templated unmasking reaction of conjugate **C13** with bis‐Key‐PNA **P6** released trace‐modified protein K33C E47C* and covalent PNA–PNA duplex **NCL‐P2**. e) BLI binding curves of PNA‐protein (**C11**–**C14**) and linker‐protein conjugates (**C15**–**C18**) at 50 nM. All conjugates were purified by SEC. SDS‐PAGE was performed without preheating under non‐reducing conditions.

Instead of testing further single Cys mutations of Neo‐2/15, we decided to modify our PNA–thioester strategy using a single Lock‐PNA with two maleimide thioester linkers to bridge two Cys residues that are placed diagonally across the IL‐2Rβ binding interface to cover a larger surface area. These Cys residues are not required to be in the periphery of the binding site, and we hoped to achieve a more general masking approach following this strategy. We examined the crystal structure again and picked four residues (L7, Y14, E47, and L51) to act as pairs with K33 as K33C modifications were fully tolerated (Figures 4a and S5).

Next, we expressed the four corresponding di‐Cys mutants of Neo‐2/15 (**L7C K33C**, **Y14C K33C**, **K33C E47C**, **K33C L51C**) in good yields. After comparing the distances between the two Cys residues in the mutant proteins (14–27 Å) and the head‐to‐tail distances of a PNA strand in duplex with another PNA,^[^ ^56^ ^]^ we decided to utilize PNAs with a length of 6 nt (head‐to‐tail ∼30 Å), which represents a compromise between sufficient binding affinity and flexibility (Figure S5). We anticipated that a shorter Lock–Key–PNA system would still provide a sufficient templating effect as templated NCL reactions have been demonstrated with PNAs as short as 4 nt.^[^ ^57^ ^]^

We then prepared bis‐Lock‐PNAs with two maleimide thioester linkers (**P4**, **P5**) as well as a complementary bis‐Key‐PNA with two Cys residues (**P6**) (Figures 4b and S5, for synthetic details see Schemes S1 and S2). Bioconjugations between di‐Cys mutants of Neo‐2/15 and bis‐Lock‐PNAs (**P4**, **P5**) were performed with fully reduced proteins to avoid the use of TCEP and its reaction with maleimides.^[^ ^58^ ^]^ We combined the bis‐maleimides **P5** or **P6** stepwise and simultaneously with the di‐Cys proteins in a solution of PBS/DMF (9:1) to achieve a temporary dilution effect in an attempt to reduce the formation of larger species (Figure 4c). We were able to obtain all four stapled PNA‐protein conjugates (**C11**–**C14**) using this strategy, albeit in moderate to low yields. As a next step, we performed the templated unmasking reaction between conjugate **C13** and Key‐PNA **P6** displaying a clean release of trace‐modified K33C E47C* and the covalent PNA‐duplex **NCL‐P2** after 1 h incubation (Figure 4d). Finally, we prepared the four maleimide thioester linker‐modified conjugates (**C15**–**C18**) of the di‐Cys mutants for BLI studies and tested all PNA‐protein and linker‐protein conjugates at 50 nM for IL‐2Rβ binding (Figure 4e). To our surprise, all tested conjugates exhibited fast binding kinetics to IL‐2Rβ and the main difference between the PNA‐stapled conjugates and their trace equivalents was the absolute response similar to the previously tested single Cys PNA‐protein conjugates (**C1**–**C4**).

After unsuccessful masking using a PNA‐stapling approach, we prepared homodimers of the four single Cys mutants of Neo‐2/15 (**H8C**, **H11C**, **Y14C**, **K33C**) with bis‐Lock‐PNA **P4** (Figure S6). The four homodimers could be effectively unmasked using Key‐PNA **P6** but similar to all other tested conjugates, apart from **C9** (D15C), no effecting masking was observed by BLI instead a dimer‐induced avidity effect could be observed (Figure S6).

### PNA‐Bridged Neo‐2/15 Dimers

**Figure 5:**
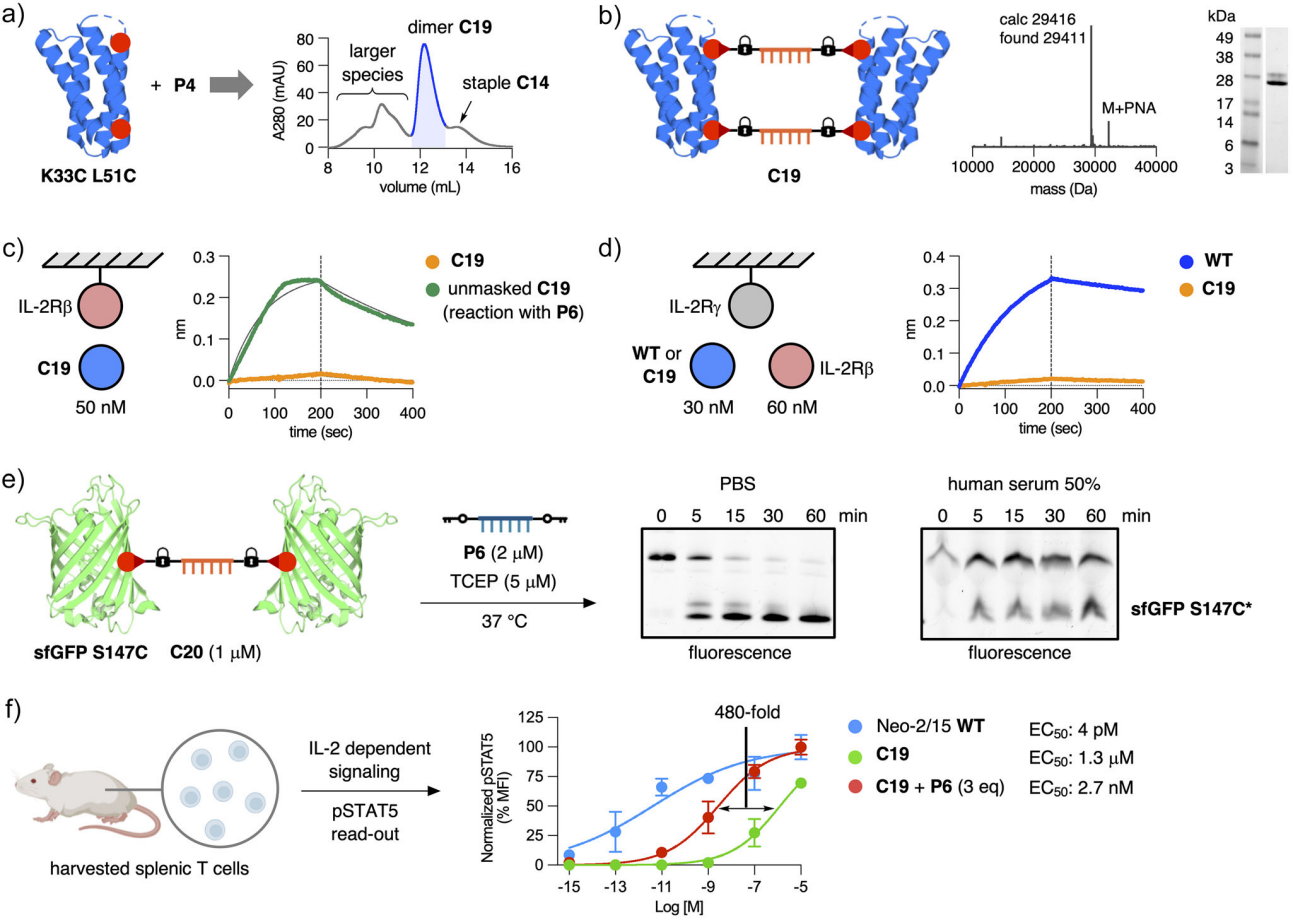
PNA‐bridged dimer of Neo‐2/15. a) SEC chromatogram of the reaction between **K33C L51C** and **P4**. b) Simplified structure and characterization of PNA‐bridged dimer **C19**. c) BLI binding curves of **C19** and **C19** treated with Key‐PNA **P6** for 45 min at 37 °C prior to BLI measurement at 50 nM. d) BLI curves with immobilized IL‐2Rγ and IL‐2Rβ in solution. Binding curves display the formation of the complex protein–IL‐2Rγ– IL‐2Rβ representative of the dimeric IL‐2R repector complex on T cells. e) Templated unmasking reaction of sfGFP homodimer **C20** with bis‐Key‐PNA **P6** in PBS and human serum–PBS (1:1). f) Dose–response curves for the determination of half‐maximal efficient concentrations (EC_50_) of **WT**, **C19,** and **C19** + **P6** (ratio 1:3) in an ex vivo T cell pSTAT5 signaling assay.

In the preparations of stapled conjugates **C13** (K33C E47C) and **C14** (K33C L51C), we observed a distinct major peak different from the stapled proteins (Figures 5a and S7). We isolated the peaks and identified them as homodimeric protein conjugates where both Cys residues of one monomer are linked to another monomer via bis‐Lock‐PNAs **P4** (Figure 5b). The dimer **C19** (K33C L51C) could be obtained in high purity, and we confirmed that the construct could be effectively unmasked using bis‐Key‐PNA **P6** (Figure S8). Unexpectedly, when we tested binding of **C19** by BLI, we found a flat response curve indicative of a weak binding interaction with IL‐2Rβ, and when the same sample was treated with bis‐Key‐PNA **P6** for 45 min, binding was restored to a similar level as linker modified conjugate **C18** (K33C L51C) (Figure 5c). We also examined the bridged dimer of **K33C E47C**, and it displayed effective masking of the binding to IL‐2Rβ in the same way as **C19**; however, we decided to move forward using **C19** (Figure S7). Next, we optimized the reaction and purification of dimer **C19** to obtain more material and conducted further binding studies by BLI (Figures 5d and S8). We immobilized IL‐2Rγ and tested binding of **C19** in the presence of IL‐2Rβ to determine if **C19** could form the dimeric receptor complex that is required for T cell signaling. We were excited to find that **C19** maintained a flat response curve in the three component BLI experiment (Figure 5d). Having a masked version of Neo‐2/15 at hand, we intended to test the conditional activation of **C19** on T cells using signal transducer and activator of transcription 5 (STAT5) phosphorylation as readout for IL‐2 like signaling.^[^ ^25^ ^]^ However, T cell experiments are generally performed in the presence of serum, and we therefore first assessed if the Key–Lock–PNA system functions under such conditions. We prepared a homodimeric conjugate **C20** of a Cys mutant of the superfolder green fluorescent protein (**sfGFP S147C**) with bis‐Lock PNA **P4** (Figures 5e and S9). Templated removal could be followed by the appearance of trace modified sfGFP S147C* as a lower molecular weight band. Intriguingly, effective release was visible after 5 min in serum applying **C20** at 1 µM (Figure 5e). In order to validate the masking effect of **C19** observed by BLI binding data, we conducted ex vivo T cell pSTAT5 signaling assays for Neo‐2/15 **WT** and **C19** (Figures 5f and S10). We purified splenic T cells of balb‐c mice and treated the cells with serial dilutions of **WT**, **C19,** or **C19** with Key‐PNA **P6**. For the unmasking reaction, a solution of **C19**/**P6** (1:3) in cell medium was incubated for 30 min prior to the assay. We were excited to see that the concentration required for full T cell activation measured by pSTAT5 was a 480‐fold higher for masked dimer **C19** (EC_50_ ∼1.3 µM) compared to **C19** treated with **P6** (EC_50_ ∼2.7 nM) (Figure 5f). The potency of **WT** (EC_50_ ∼4.1 pM) could not be reached by released trace‐modified K33C L51C* probably as a result of incomplete unmasking. We also confirmed successful unmasking of **C19** and increased STAT5 phosphorylation using a T lymphoblast cell line (HH) (Figure S10). Having successful applied our Key–Lock–PNA masking approach on Neo‐2/15, we were interested in utilizing the system for further protein‐based immuno‐oncology applications.

### PNA‐Masked Human CD40 DARPin

**Figure 6:**
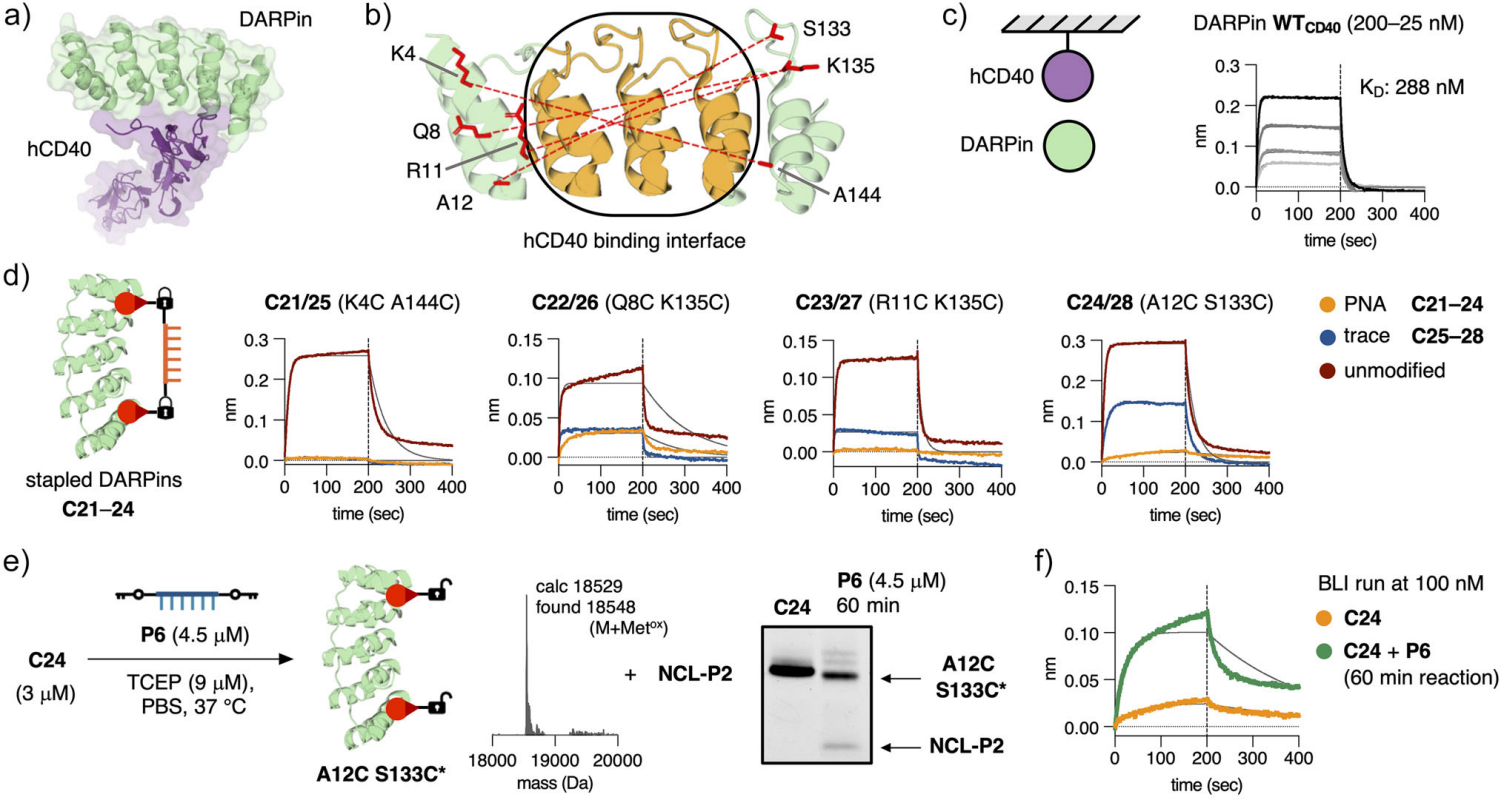
PNA‐masked hCD40 DARPin. a) Crystal structure of DARPin bound to hCD40 (PDB: 7P3I). b) Expressed double Cys mutants of hCD40 DARPin (**K4C A144C**, **Q8C K135C**, **R11C K135C**, **A12C S133C**). c) BLI binding curves of DARPin **WT_CD40_** to immobilized hCD40 at 200–25 nM. d) BLI binding curves at 100 nM of unmodified double Cys mutants of hCD40 DARPin, PNA‐stapled conjugates (**C21**–**C24**) and maleimide thioester linker conjugates (**C25**–**C28**) to hCD40. e) Unmasking reaction of conjugate **C24** with bis‐Key‐PNA **P6** released trace‐modified protein A12C S133C* and covalent PNA–PNA duplex **NCL‐P2**. f) BLI binding curves of PNA‐stapled conjugate **C24** before and after unmasking with **P6** at 100 nM. All conjugates were purified by SEC. SDS‐PAGE was performed without preheating under non‐reducing conditions.

DARPins are small, single domain proteins with high affinities for their targets that can be discovered through display techniques.^[^ ^59^, ^60^, ^61^ ^]^ The proteins are rigid, have no internal Cys residues, and the repeat unit structure places the variable residues during selection always on same face of the proteins. Further, the N‐ and C‐terminal repeat units remain nearly identical for all DARPins of one family, which could enable a more general masking strategy for these class of proteins if non‐binding, but conserved residues can be used to install Lock‐PNAs. We chose to exemplify our Key–Lock–PNA masking system on a recently reported DARPin (**WT_CD40_**) binding to the human CD40 receptor (Figures 6 and S11).^[^ ^62^ ^]^ CD40 activation on dendritic cells can evoke antitumoral activity, although therapies can suffer from systemic activation,^[^ ^63^ ^]^ making the reported DARPin (**WT_CD40_**) an ideal proof‐of‐concept target for the development a PNA‐based DARPin masking strategy.

We first examined the crystal structure of **WT_CD40_** bound to hCD40 (Figure 6a) and chose four pairs of residues as positions for Cys mutations on the conserved terminal repeat units that span over the hCD40 binding site (Figure 6b). The distances between the residues were 32–36.6 Å, which we expected to be compatible with the 6 nt bis‐Lock PNA **P4**. Next, we expressed **WT_CD40_** and the four double Cys mutants (**K4C A144C**, **Q8C K135C**, **R11C K135C**, **A12C S133C**) in good yields and confirmed effective binding of **WT_CD40_** to hCD40 by BLI (Figure 6c). Using the same procedures as for Neo‐2/15, we prepared the four PNA‐stapled conjugates (**C21**–**C24**) in moderate yields as well as the corresponding maleimide thioester linker‐modified conjugates (**C25**–**C28**) and conducted BLI binding assessments at 100 nM (Figure 6d).

All di‐Cys mutants of **WT_CD40_** gave strong response curves but in contrast to the Neo‐2/15 mutants, all linker‐protein conjugates, except **C28** (A12C S133C), abolished or significantly reduced the response to hCD40. Following the same trend, all PNA‐stapled conjugates prohibited effective binding to hCD40, making **A12C S133C** a promising mutant for PNA‐masking (Figure 6d). The unmasking reaction of **C24** with bis‐Key‐PNA **P6** proceed smoothly to release trace‐modified A12C S133C*, highlighting the general application of the Key–Lock–PNA release observed for all tested conjugates (Figure 6e). Finally, we conducted an BLI experiment with **C24** before and after 45 min incubation with **P6** and intriguingly found a significant change in binding response to hCD40 as a result of the unmasking step (Figure 6f).

## Conclusion

In conclusion, we developed a new strategy for the conditional activation of therapeutic proteins using a proximity‐induced removal step of PNA masking groups exemplified on the de novo cytokine Neo‐2/15 and a DARPin binding hCD40. We established an efficient method to prepare maleimide–thioester containing PNAs that enable fast and site‐specific maleimide bioconjugation to Cys residues, whereas at the same time having a cleavable linker for a templated NCL removal reaction. The thioester linkage maintained acceptable stability in human serum and could be cleaved within minutes at low micromolar concentrations and stochiometric amounts using a Cys‐containing complementary PNA. The chemistry was applied to several Cys mutants of Neo‐2/15 although binding to its target could not be prevented. Further, a double Cys mutation strategy was applied for the installation of PNA staples onto proteins using bis‐maleimide thioester PNAs. Four PNA‐stapled variants of Neo‐2/15 were produced as well as four PNA‐stapled variants of DARPin **WT_CD40_**, and in all tested cases selective removal at low concentrations furnished the unmasked proteins. Binding of Neo‐2/15 to IL‐2Rβ could also not be prevented using PNA stapling but in case of DARPin **WT_CD40_**, one double Cys mutant **A12 S133C** could be selectively masked and unmasked on‐demand. Finally, we discovered that a double bridged dimer **C19** of a double Cys mutant of Neo‐2/15 **K33C L51C** weakened the binding affinity of Neo‐2/15 to IL‐2Rβ significantly as well as to the complex of IL‐2Rβ–IL‐2Rγ. The masked construct could be cleanly unmasked to release the active mutant of Neo‐2/15, which was confirmed in a T cell pSTAT5 signaling assay. The successful conditional activation of a masked Neo‐2/15 therapeutic through templated NCL under physiological conditions (resulting in a 480‐fold in potency for T cell activation) could enable higher dosage and combining this with the targeted delivery of one of the two components **C19** or **P6** might help to mitigate toxicity. We expect that this system could be applied to more therapeutic proteins, for example, other DARPins, and potentially be used to address the problem of systemic immune cell activation in cytokine and other immuno‐oncology therapies.

## Supporting Information

Supporting Information file encompasses supporting figures and schemes depicting the synthesis of compounds used, experimental methods, chemical synthesis and compound characterization data, as well as copies of LCMS traces and ^1^H and ^13^C NMR spectra, copies of SDS‐PAGE gels, and deconvoluted mass spectra of proteins.

## Conflict of Interests

The authors declare no conflict of interest.

## Supporting information



Supporting Information

## Data Availability

The data that support the findings of this study are available from the corresponding authors upon reasonable request.
