# Supplementary material for: Conditional Activation of Protein Therapeutics by Templated Removal of Peptide Nucleic Acid Masking Groups
Source: Angew Chem Int Ed Engl. 2025 Mar 17;64(21):e202502268. doi: 10.1002/anie.202502268 (PMC12087824; doi:10.1002/anie.202502268)
Supplement: Supplementary file 1 — Supporting Information [file ANIE-64-e202502268-s001.pdf]

Supporting Information

Table of Contents

Supporting Figures 3

Supporting Schemes 14

Materials and Methods 17

General Information and Instrumentation 17

Chemical Synthesis of Thioester Linkers 20

Synthesis of Peptide Nucleic Acids (PNAs) 22

General Procedures for Solid-Phase Synthesis (SPS) of PNAs 22

Synthesized Lock-PNAs 23

Synthesized Key-PNAs 26

Fluorophore-Labelled Reagents 27

Protein Expression in *E. coli* 28

Characterization of Recombinant Proteins 30

Chemical Modifications of Proteins 38

General Bioconjugation Procedures 38

Neo-2/15 Linker Conjugates 39

Neo-2/15 PNA Conjugates 45

DARPin Linker Conjugates 54

DARPin PNA Conjugates 56

Fluorescent Conjugates for Serum Studies 59

Binding and Stability Assays of Bioconjugates 62

Biolayer Interferometry (BLI) Measurements 62

Serum Stability Determination 62

Templated Removal of PNA Masking Groups 62

Ex vivo T cell STAT5 Signaling Assay 63

NMR Spectra of Synthesized Compounds 64

Uncropped SDS-PAGE gels 67

References 72

Supporting Figures

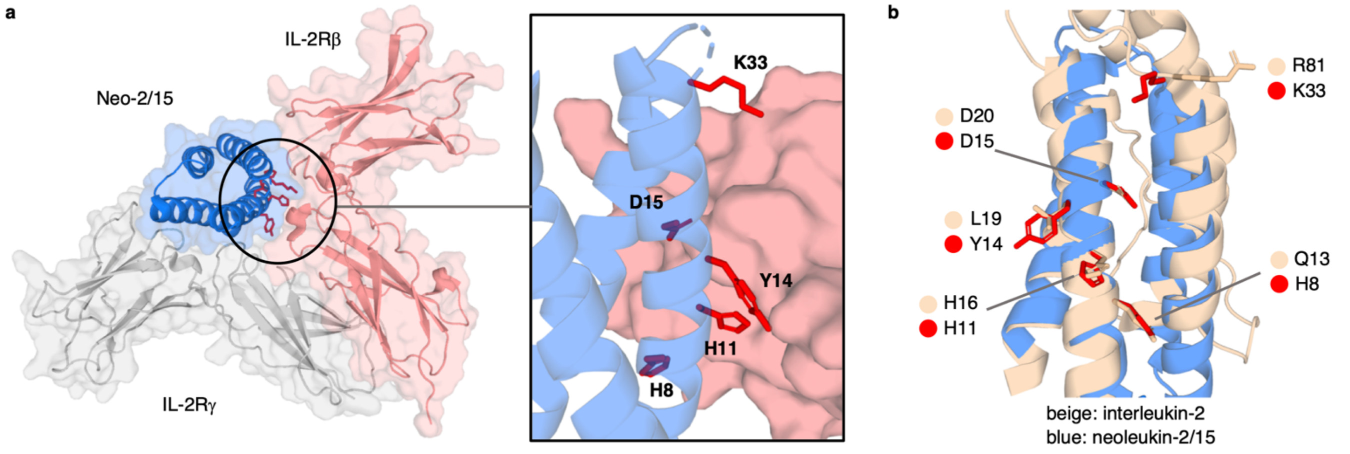

Supporting Figure S1. Crystal structure and mutated residues of neoleukin-2/15 (Neo-2/15). a, Crystal structure of Neo-2/15 bound to IL-2 receptor chain beta (IL-2Rβ, CD122) and chain gamma (IL-2Rγ, CD132) (PDB: 6DG5).[1] IL-2 signaling with Neo-2/15 is initiated stepwise by first forming the complex with IL-2Rβ, which then binds to IL-2Rγ leading to intracellular phosphorylation of signal transducer and activator of transcription 5 (STAT5) further downstream effect. Residues in red are located in the periphery of the IL-2Rβ binding interface and were chosen to as cysteine (Cys) mutations for the peptide nucleic acid (PNA) masking strategy. **b**, Overlay of crystal structures Neo-2/15 (PDB: 6DG5) and interleukin-2 (IL-2) (PDB: 2ERJ).

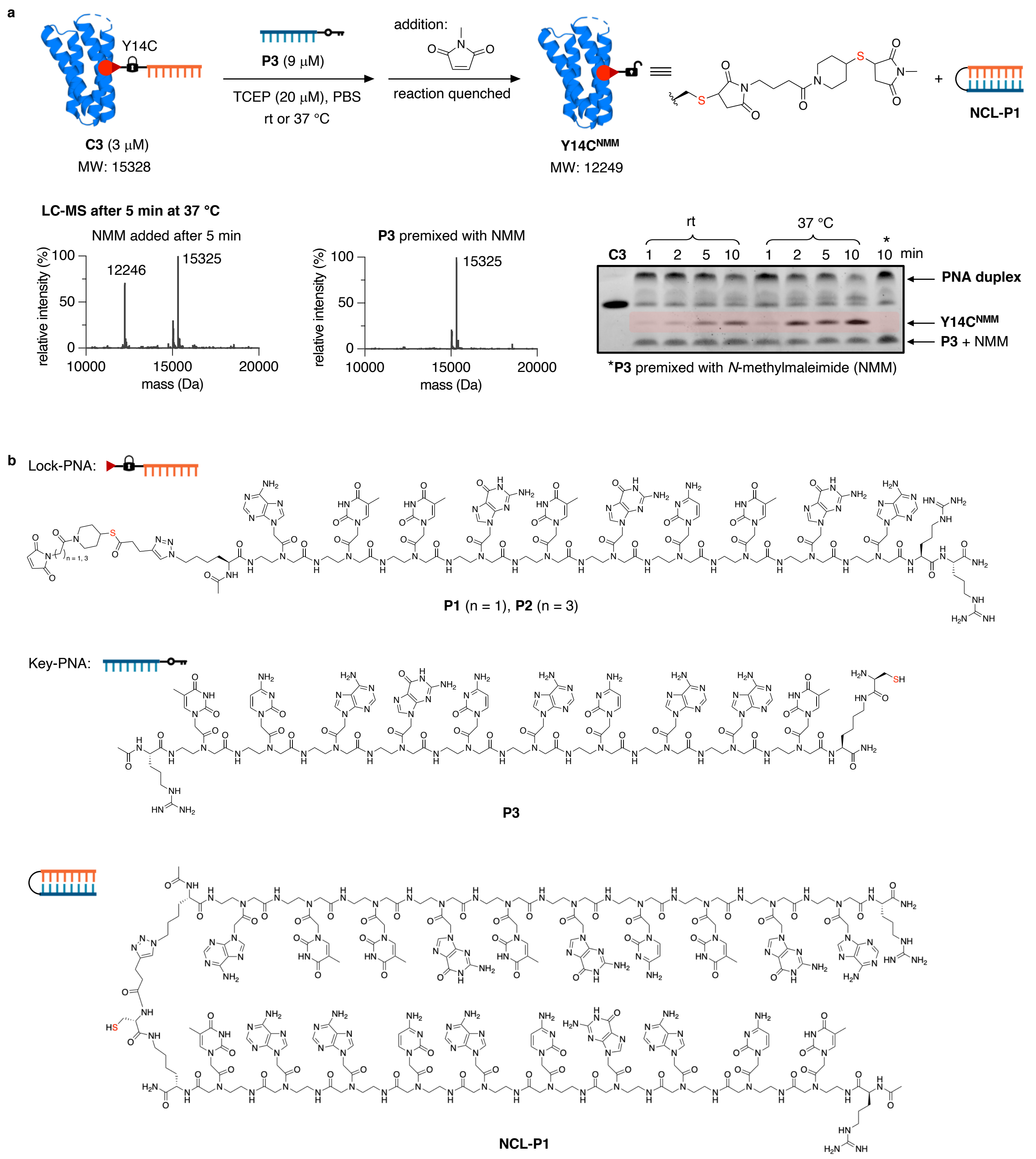

Supporting Figure S2. Templated native chemical ligation (NCL) for PNA removal. a, PNA-conjugate **C3** was incubated with Key-PNA **P3** in phosphate-buffered saline (PBS, pH 7.4) in the presence of tris(2-carboxyethyl)phosphine (TCEP) at room temperature (rt) or 37 °C. Reaction aliquots were treated with *N*-methylmaleimide (NMM) to block the Cys residue on **P3** and thereby quench the reaction. ***P3** was pre-mixed with NMM before the addition to **C3**. LC-MS deconvoluted mass spectra confirmed the mass of the released and NMM-modified protein **Y14C^{NMM}** as well as the requirement of a reactive cysteine residue on Key-PNA **P3**. **b**, Full structures of Lock-PNAs **P1** and **P2**, Key-PNA **P3** and the reaction product of the NCL reaction **NCL-P1**.

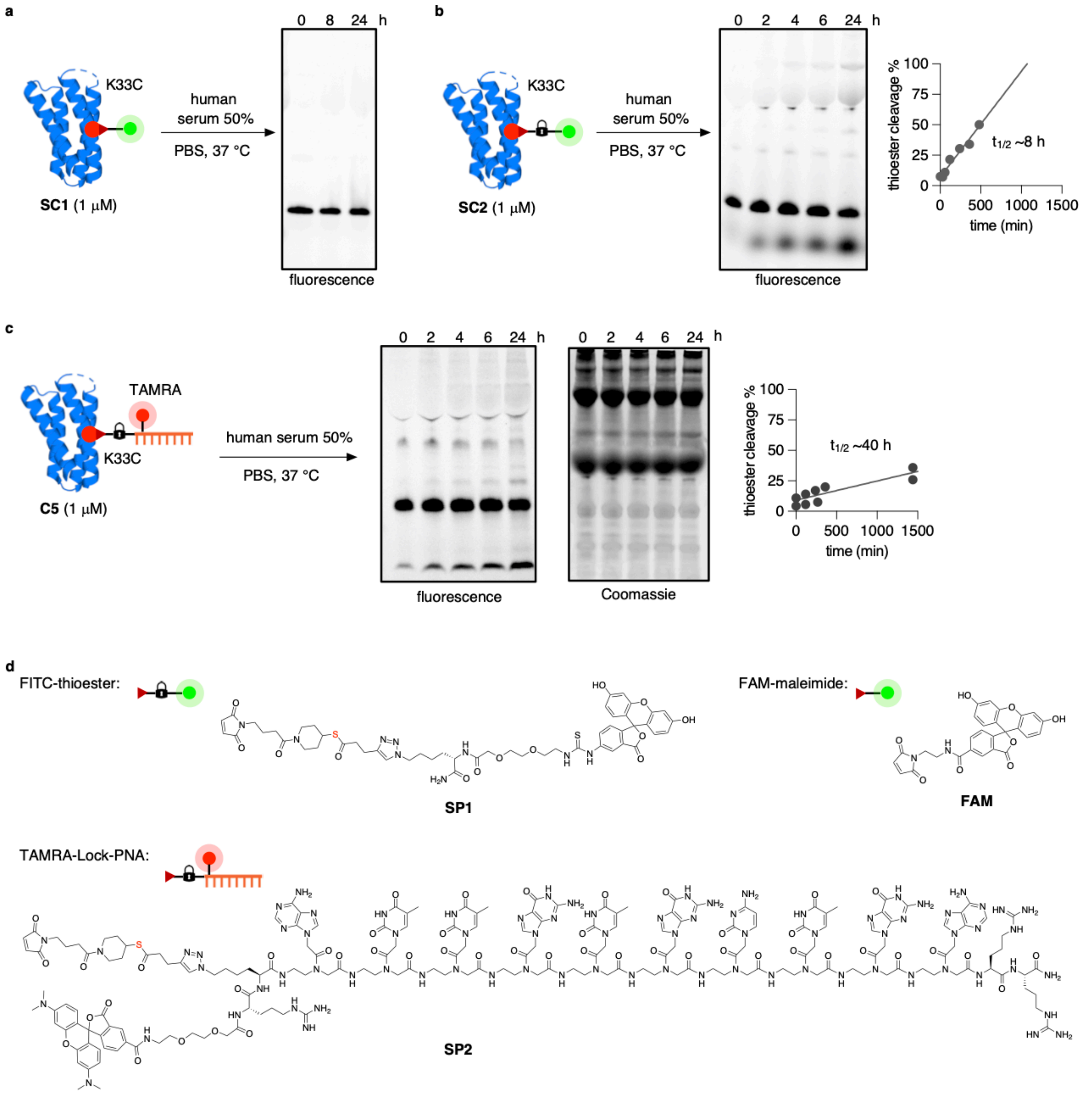

Supporting Figure S3. Serum stability of fluorescent Neo-2/15 conjugates. a, Incubation of control conjugate **SC1** (Neo-2/15 **K33C–FAM**) in human serum–PBS (1:1) at 37 °C to account for deconjugation by the retro-Michael reaction of maleimides. **b**, Incubation of conjugate **SC2** (Neo-2/15 **K33C–SP1**) in human serum–PBS (1:1) at 37 °C. A representative gel is shown, and the experiment was performed in duplicate with different time points. **c**, Incubation of conjugate **C5** (Neo-2/15 **K33C–SP2**) in human serum–PBS (1:1) at 37 °C. A representative gel is shown, and the experiment was performed in duplicate. **d**, Full structures of fluorescein isothiocyanate (FITC)-thioester **SP1**, fluorescein-maleimide (**FAM**) and tetramethylrhodamine (TAMRA)-labelled Lock-PNA (**SP2**). Half-life ($t_{1/2}$) was estimated by band intensity compared to t = 0 h using the software Image Lab. Conjugates were purified by size exclusion chromatography (SEC). Sodium dodecyl sulfate polyacrylamide gel electrophoresis (SDS-PAGE) was performed without preheating under non-reducing conditions.

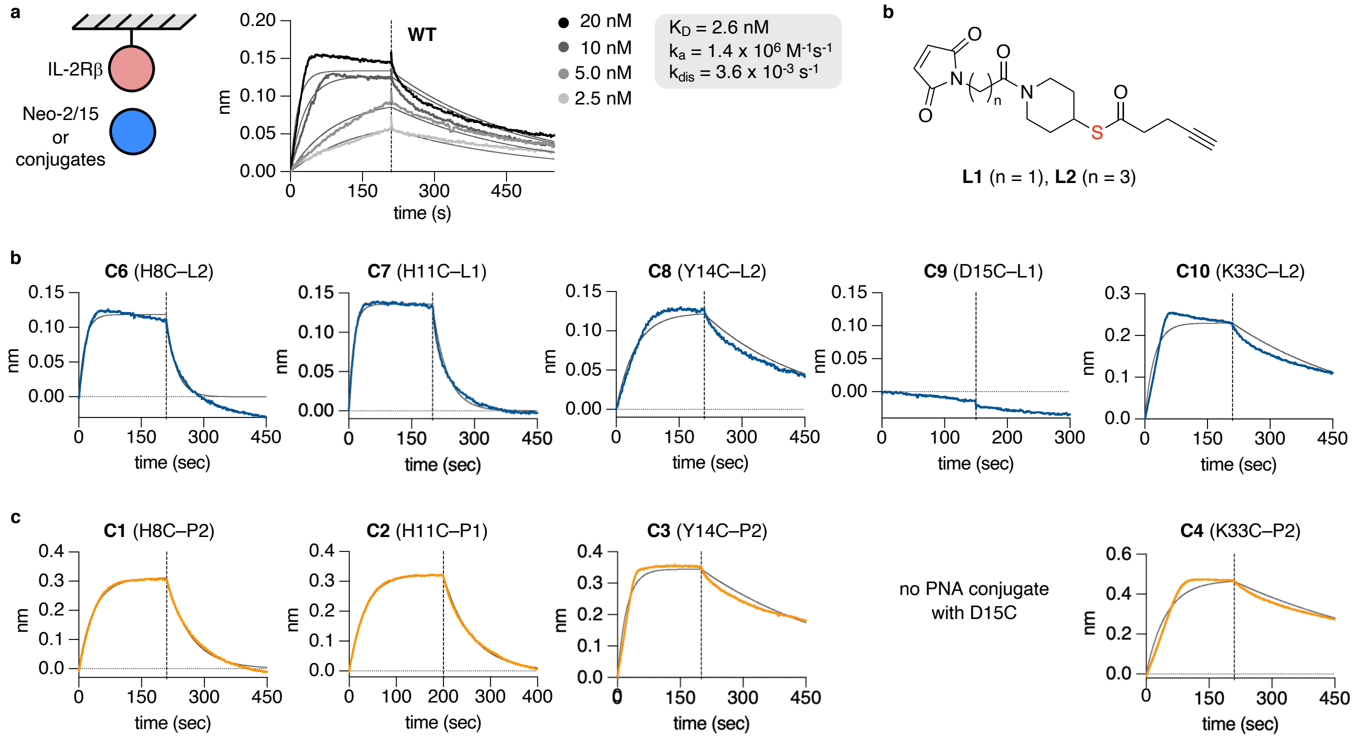

Supporting Figure S4. BLI measurements of Neo-2/15 conjugates. **a**, BLI measurement were performed with immobilized human IL-2Rβ. Neo-2/15 **WT** was tested at concentration range (20–2.5 nM). **b**, Structure of maleimide thioester linkers **L1** and **L2** used for the preparation of linker modified conjugates of Neo-2/15 (**C6**–**C10**). **c**, Binding curves of linker-protein conjugates (**C6**–**C10**) obtained with 50 nM of conjugate. **d**, Binding curves of PNA-protein conjugates (**C1**–**C4**) obtained with 50 nM of conjugate.

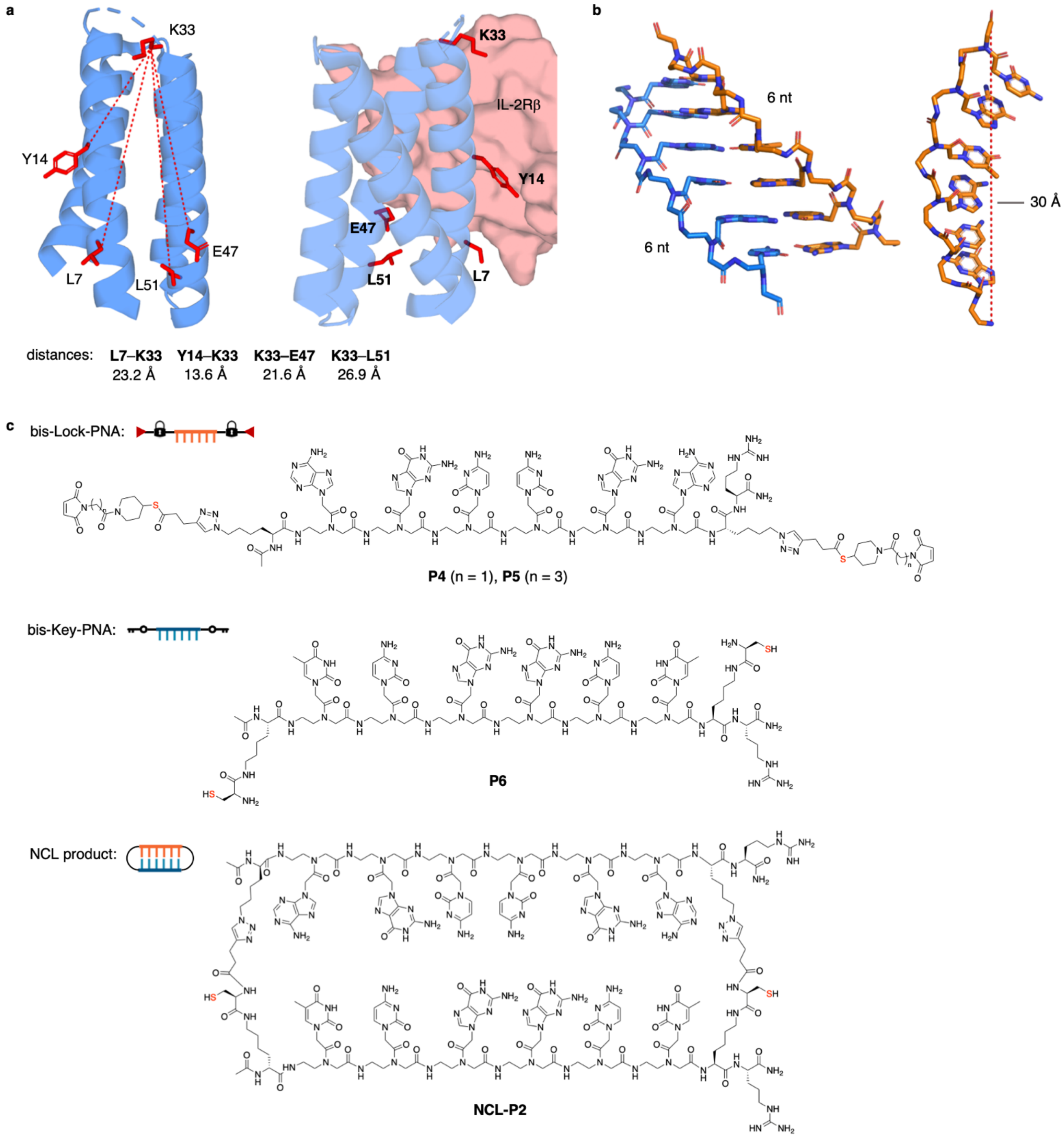

Supporting Figure S5. Double Cys approach for PNA stapling of Neo-2/15. a, Residues in red were chosen as double Cys mutations for a PNA stapling strategy using bis-maleimide thioester containing Lock-PNAs (**P4**, **P5**). Distances were measured between C_β of the amino acid residues. **b**, Crystal structure of a PNA-PNA complex (PDB: 3MBS).[2] The length of a 6 nt PNA (30 Å) annealed to another PNA was measured from the C- to the N-terminus. **c**, Full structures of bis-Lock-PNAs **P4** and **P5**, bis-Key-PNA **P6** and the reaction product of the NCL reaction **NCL-P2**.

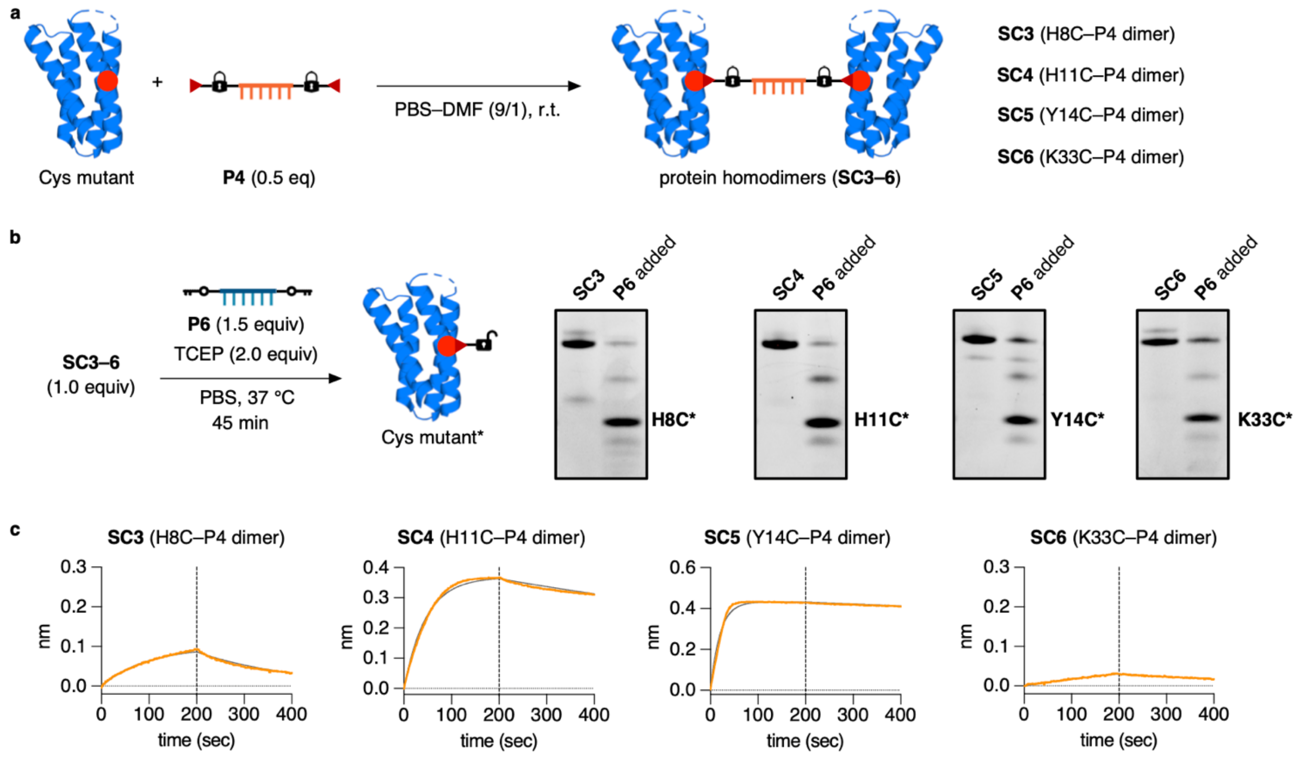

Supporting Figure S6. Single PNA-bridged homodimers of single Cys mutants of Neo-2/15. a, Dimer preparations of Cys mutants of Neo2/15 (**SC3**–**SC6**) were performed with fully reduced proteins in the absence of tris(2-carboxyethyl)phosphine (TCEP) through the slow addition of protein to a solution of bis-Lock-PNA **P4** (0.5 equivalents compared to protein) in PBS–DMF (9:1). **b**, Templated unmasking reaction of dimer conjugates **SC3**–**SC6** performed at concentrations of 2–4 μM with bis-Key-PNA (**P6**) released trace-modified proteins effectively in 45 min at 37 °C. **c**, BLI binding curves with immobilized human IL-2Rβ of dimer conjugates **SC3**–**SC6** performed with 50 nM of conjugate. Conjugates were purified by SEC. SDS-PAGE was performed without preheating under non-reducing conditions.

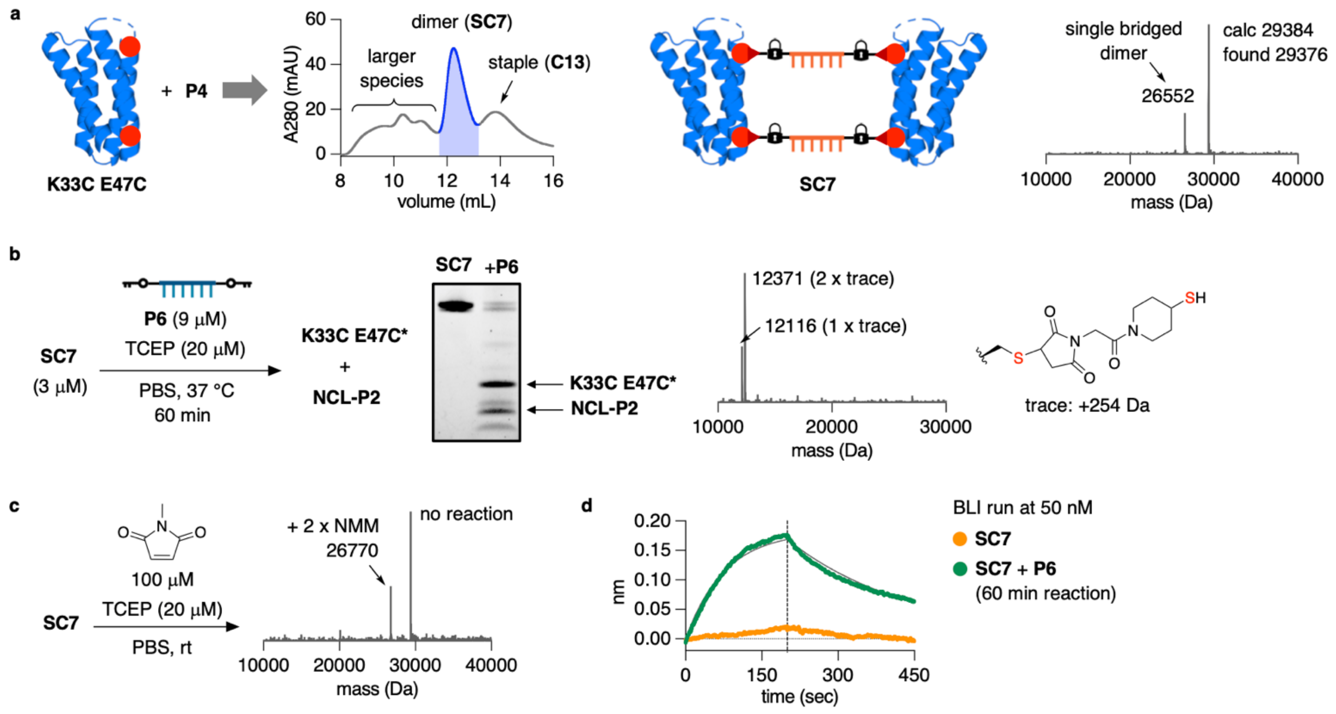

Supporting Figure S7. Double PNA-bridged homodimer of Neo-2/15 K33C E47C. a, SEC chromatogram of the reaction between **K33C E47C** and **P4** as well as the simplified structure and characterization of PNA-bridged dimer (**SC7**). The purified conjugate contained single-bridged dimer with two Cys being forming a disulfide bond. **b**, Templated unmasking reaction of conjugate **SC7** with bis-Key-PNA **P6** released protein **K33C E47C** modified with 1–2 trace molecules and covalent PNA-PNA duplex **NCL-P2**. **c**, PNA-bridged dimer **SC7** was treated with NMM and TCEP and the addition of 2 NMM molecules to the single-bridged dimer was observed while no reaction occurred the double-bridged dimer. **d**, BLI binding curves with immobilized human IL-2Rβ of **SC7** and **SC7** treated with Key-PNA **P6** for 60 min at 37 °C prior to BLI measurement at 50 nM. Conjugates were purified by SEC. SDS-PAGE was performed without preheating under non-reducing conditions.

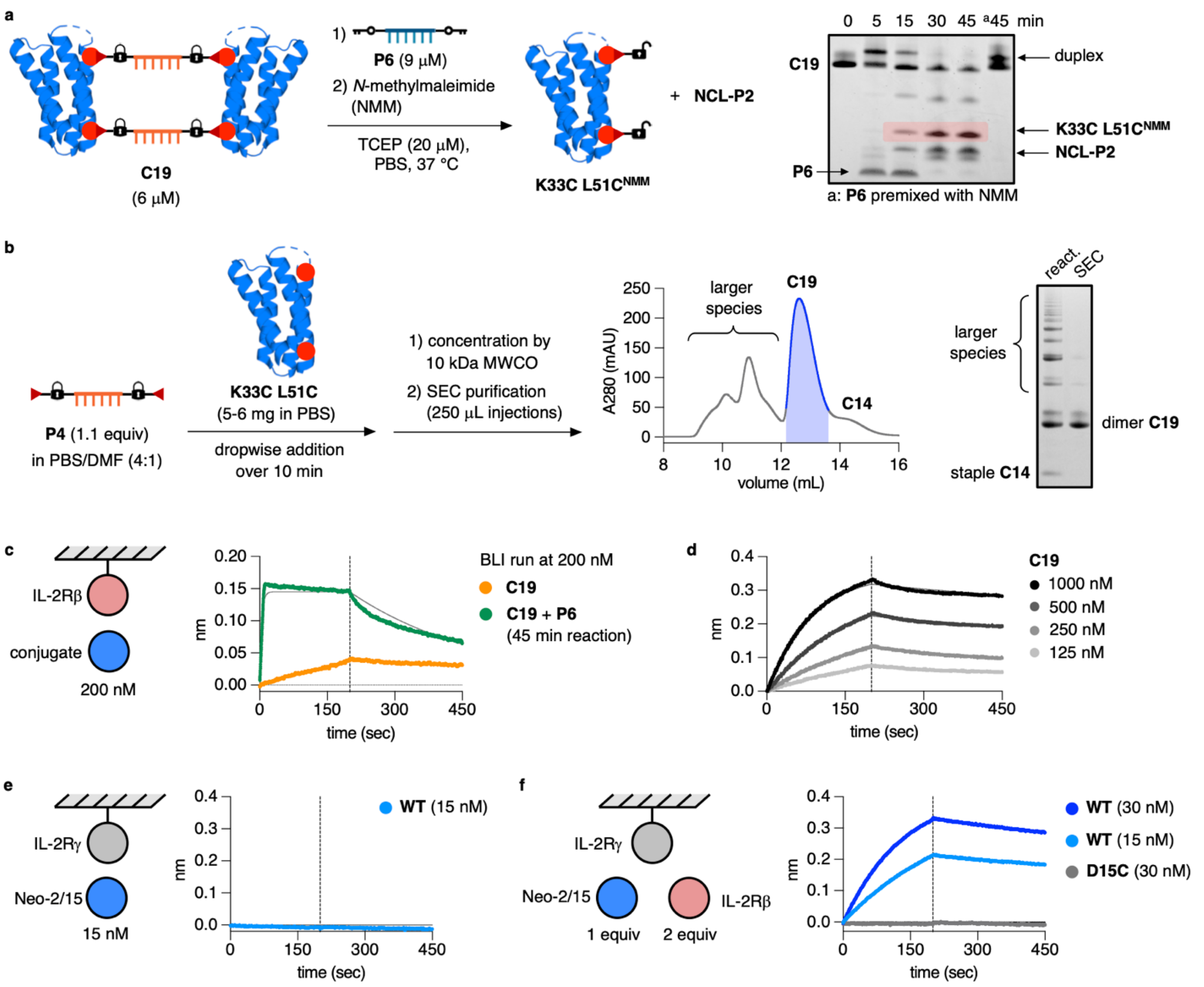

Supporting Figure S8. Double PNA-bridged homodimer of Neo-2/15 K33C L51C. a, Templated unmasking reaction of conjugate **C19** with bis-Key-PNA **P6**. Reaction aliquots were treated with NMM to block the Cys residues on **P6** and modify the released protein **K33C L51C**^{NMM}. ^{a}**P6** was pre-mixed with NMM before the addition to **C19**. **b**, Larger scale preparations of **C19** were performed by dropwise addition of fully reduced protein to a solution of **P4** in PBS–DMF (4:1) followed by a concentration step using 10 kDa molecular weight cut-off (MWCO) centrifugal filters. SEC purifications were performed with injection volumes of 0.25 mL to maintain sufficient resolution between peaks. **c**, BLI binding curves with immobilized human IL-2Rβ of **C19** and **C19** treated with Key-PNA **P6** for 45 min at 37 °C at 200 nM. **d**, BLI binding curves with immobilized human IL-2Rβ of **C19** at a concentration range (1000–125 nM). **e**, BLI binding curve with immobilized human IL-2Rγ of Neo2/15 **WT** at 15 nM. **f**, BLI binding curve with immobilized human IL-2Rγ of Neo2/15 **WT** and **D15C** in the presence of solubilized IL-2Rβ at 15 nM and 30 nM. Conjugates were purified by SEC. SDS-PAGE was performed without preheating under non-reducing conditions.

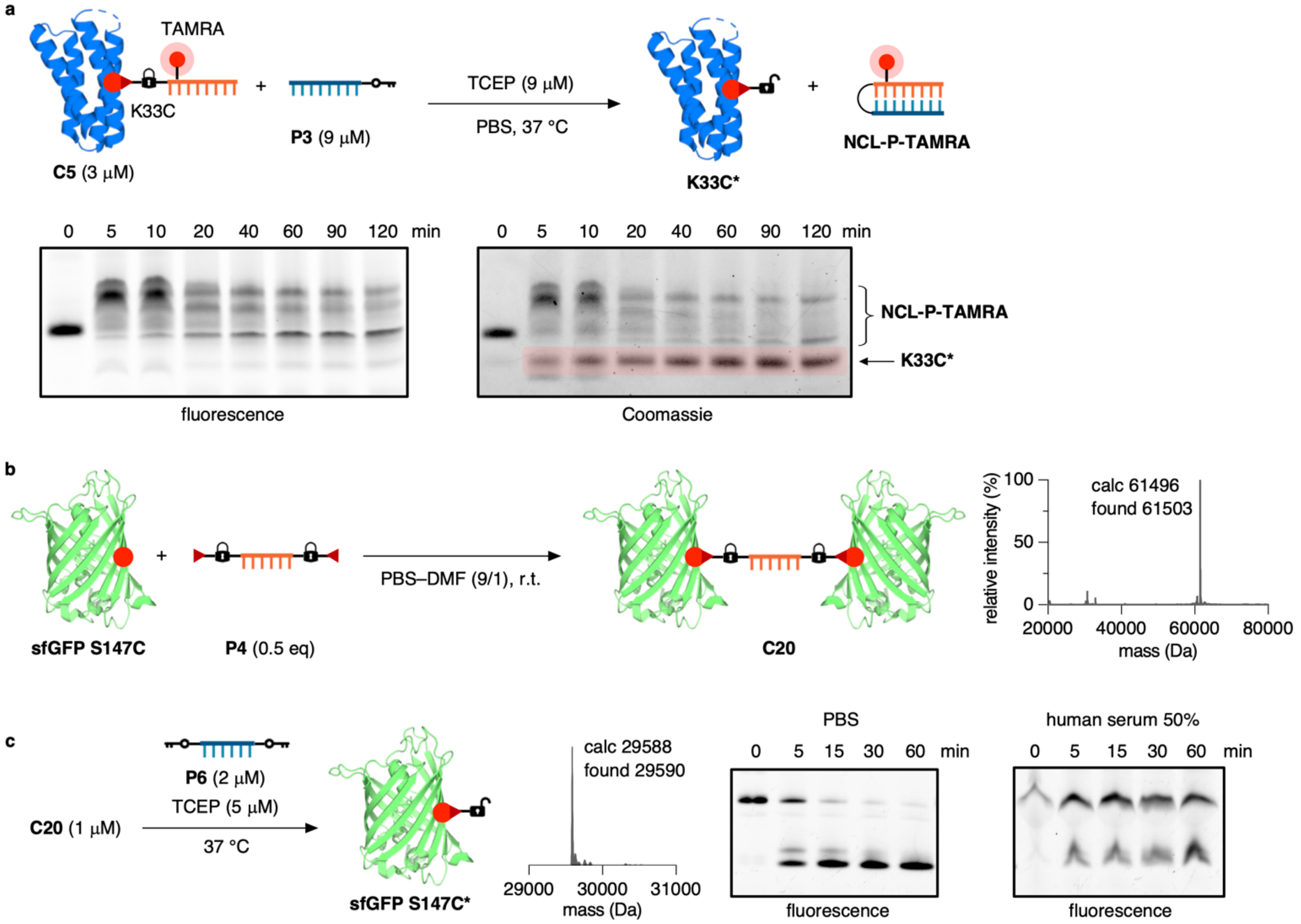

Supporting Figure S9. Templated unmasking reaction in human serum. a, Templated unmasking reaction of fluorescent conjugate **C5** with Key-PNA **P3** in PBS released trace-modified protein **K33C*** but the release could not be followed satisfactory by fluorescence due to the broad bands of the TAMRA-labeled NCL product **NCL-P-TAMRA**. **b**, Homodimer **C20** preparation of the Cys mutant S147C of the superfolder green fluorescent protein (**sfGFP S147C**) was performed with fully reduced protein in the absence of TCEP through the slow addition of protein to a solution of bis-Lock-PNA **P4** (0.5 equivalents compared to protein) in PBS–DMF (9:1). **c**, Templated unmasking reaction of sfGFP homodimer **C20** with bis-Key-PNA **P6** in PBS and human serum–PBS (1:1).

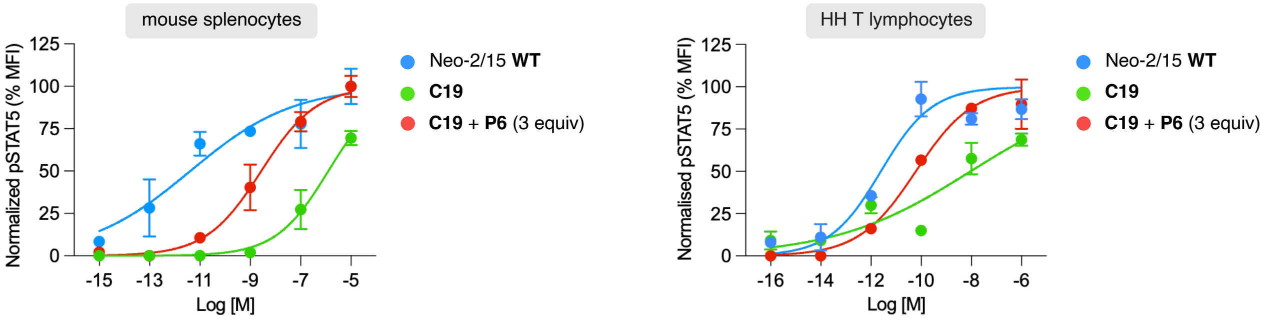

WT (EC_{50}): 4.1 pM (0.5–35 pM 95% CI)
C19 (EC_{50}): 1.3 µM (0.7–2.2 µM 95% CI)
C19 + **P6** (EC_{50}): 2.7 nM (1.3–5.7 nM 95% CI)

WT (EC_{50}): 2.7 pM (0.5–11 pM 95% CI)
C19 (EC_{50}): 9.3 nM (0.9–218 nM 95% CI)
C19 + **P6** (EC_{50}): 61 pM (26–143 pM 95% CI)

Supporting Figure S10. *Ex vivo* T cell pSTAT5 singaling assay. T cell assays were performed with purified mouse T splenocytes and T lyphmocytes (HH cell line). Cells were treated with Neo-2/15 **WT**, PNA-bridged dimer **C19** and **C19** treated with Key-PNA **P6** (3.0 equiv for 30 min in cell medium). Half-maximal efficient concentrations (EC_{50}) are reproted with their 95% confidence interval (95% CI). Error bar represent the standard diviation (SD) of two biological replicates.

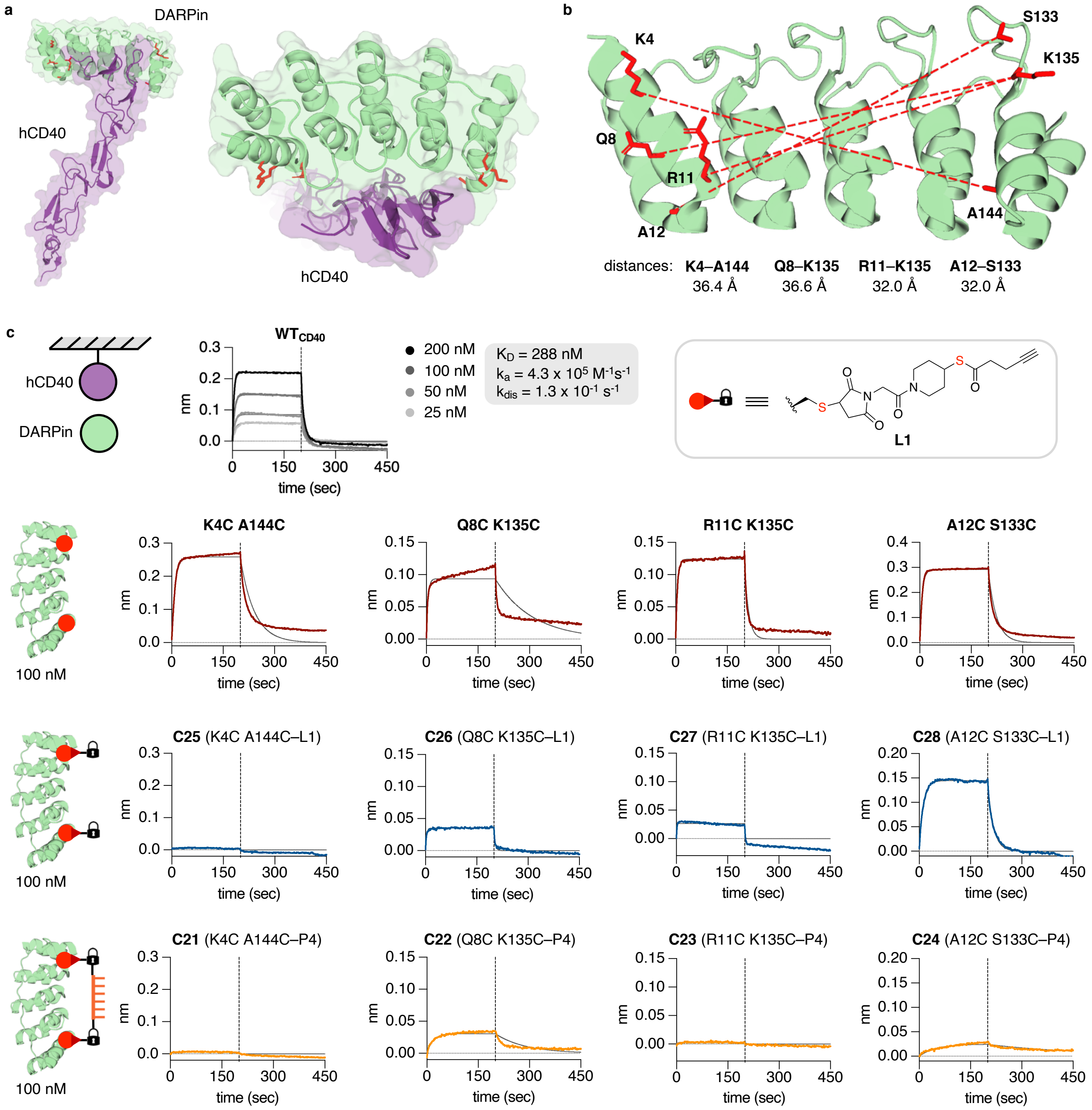

Supporting Figure S11. PNA-masking of human CD40 DARPin. a, Crystal structure of DARPin $\mathbf{WT_{CD40}}$ bound to human CD40 (PDB: 7P3I).[3] **b**, Residues in red were chosen as double Cys mutations for a PNA stapling strategy using bis-maleimide thioester-containing Lock-PNA **P4**. Distances were measured between C_β of the amino acid residues. **c**, BLI binding curves with immobilized human CD40 (Q21–R193) of unmodified double Cys mutants of DARPin $\mathbf{WT_{CD40}}$ (200–25 nM) as well as PNA-stapled conjugates (**C21**–**C24**) and maleimide thioester linker conjugates (**C25**–**C28**) at 100 nM. Conjugates were purified by SEC.

Supporting Schemes

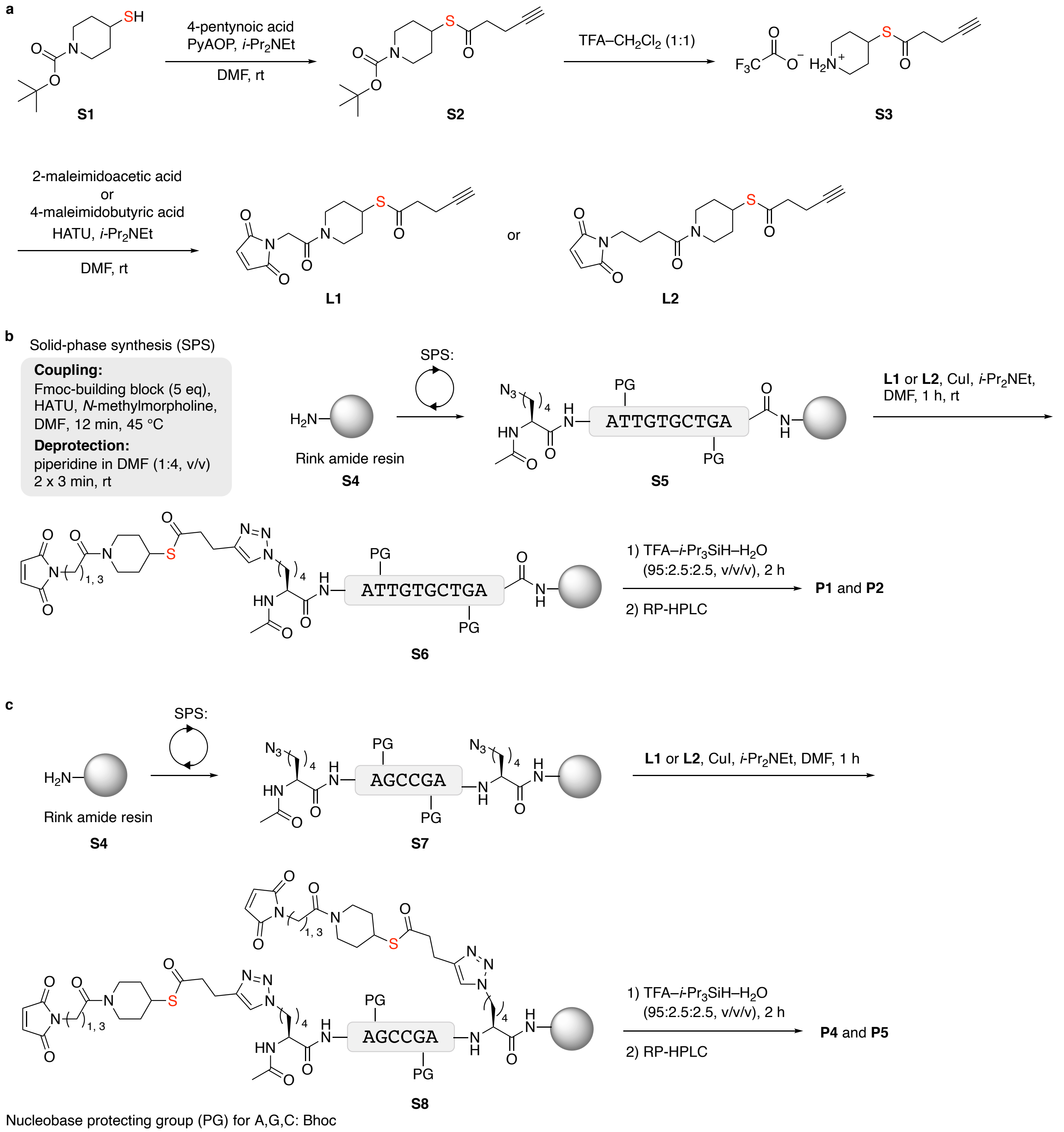

Supporting Scheme S1. Synthesis of Lock-PNAs. **a**, Synthesis of maleimide thioester linkers **L1** and **L2**. **b**, Solid-phase synthesis (SPS) of Lock-PNAs **P1** and **P2**. SPS was performed on TentaGel Rink amide resin **S4** to obtain resin-bound azide-containing oligomer **S5**, which was converted to resin-bound maleimide-thioester oligomers **S6** via copper-catalyzed azide–alkyne cycloaddition (CuAAC) with **L1** or **L2**. **c**, SPS of bis-Lock-PNAs **P4** and **P5**. SPS was performed on TentaGel Rink amide resin **S4** to obtain resin-bound di-azide-containing oligomer **S7**, which was converted to resin-bound bis-maleimide-thioester oligomers **S8** via CuAAC with **L1** or **L2**. Trifluoroacetic acid (TFA) cleavage and RP-HPLC purification afforded **P1**, **P2**, **P4** and **P5**.

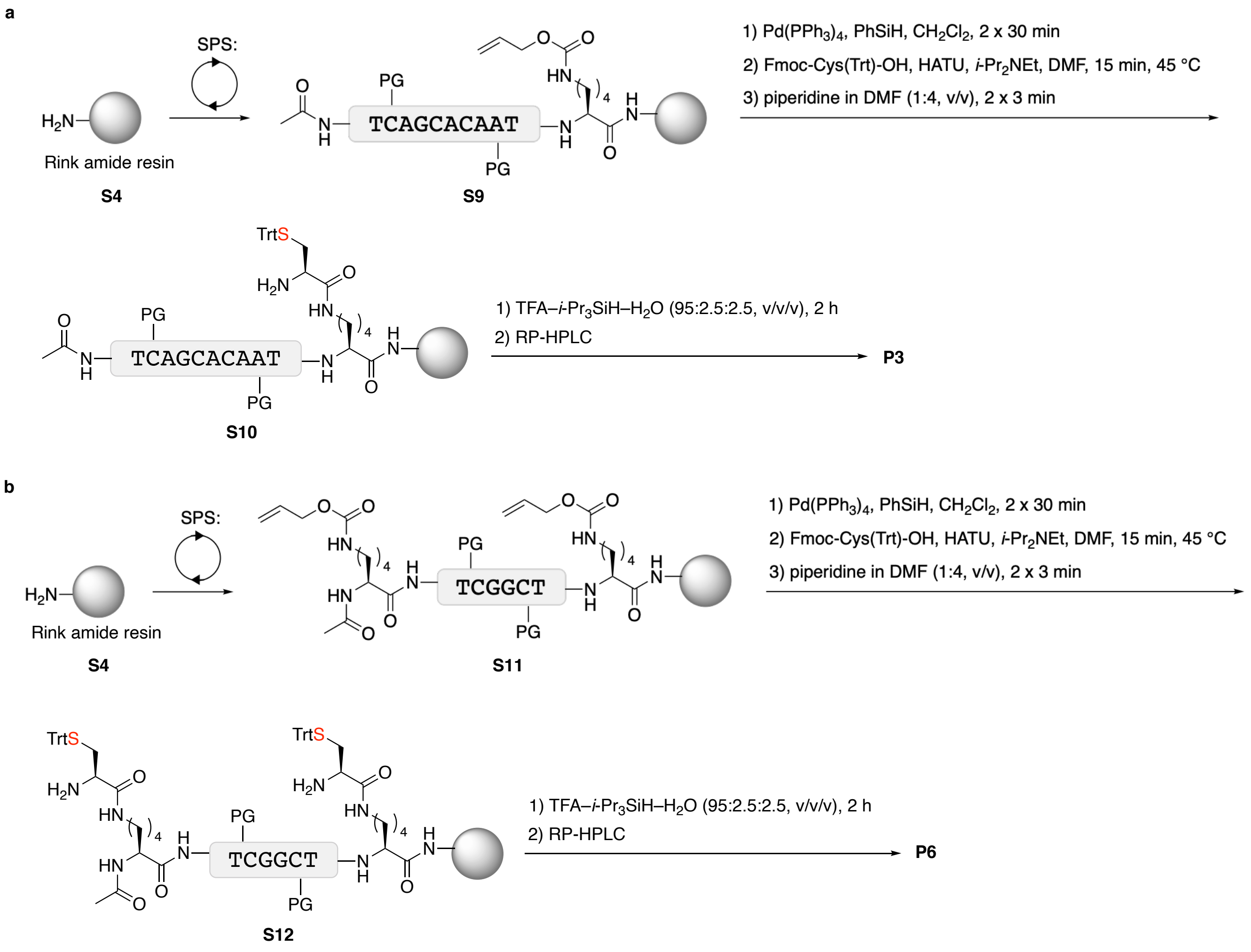

Nucleobase protecting group (PG) for A,G,C: Bhoc

Supporting Scheme S2. Synthesis of Key-PNAs. a, SPS of Key-PNA **P3**. SPS was performed on TentaGel Rink amide resin **S4** to obtain resin-bound allyloxycarbonyl (Alloc)-Lys oligomer **S9**, which was converted to resin-bound cysteine-containing oligomers **S10** via Alloc deprotection followed by coupling of Fmoc-Cys(Trt)-OH. **b**, SPS of bis-Key-PNA **P6**. SPS was performed on TentaGel Rink amide resin **S4** to obtain resin-bound bis-(Alloc)-Lys oligomer **S11**, which was converted to resin-bound bis-cysteine-containing oligomers **S12** via Alloc deprotection followed by coupling of Fmoc-Cys(Trt)-OH. TFA cleavage and RP-HPLC purification afforded **P3** and **P6**.

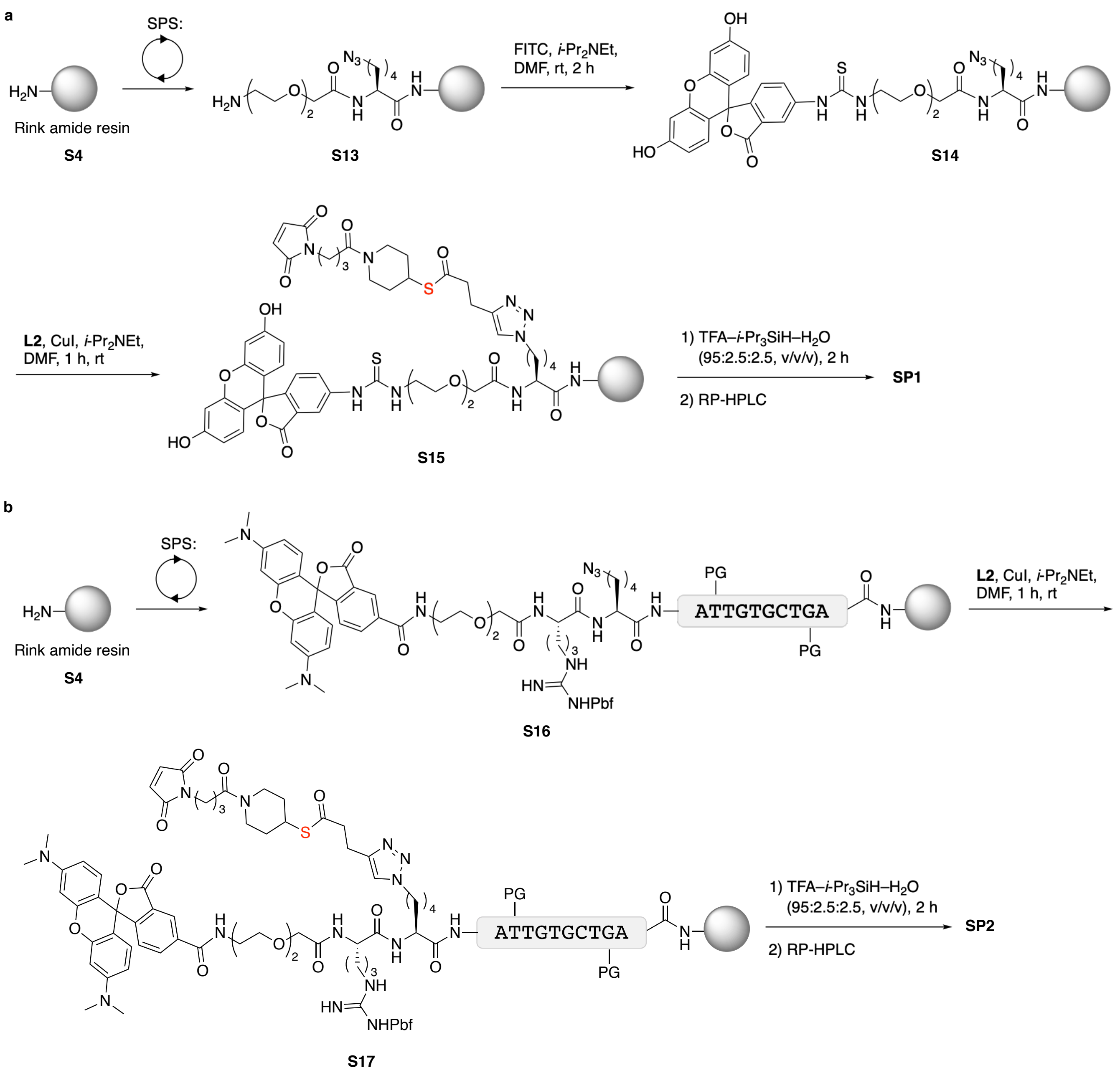

Nucleobase protecting group (PG) for A,G,C: Bhoc

Supporting Scheme S3. Synthesis of fluorescent linker and PNA. a, SPS of fluorescein 5-isothiocyanate (FITC)-labeled linker **SP1**. SPS was performed on TentaGel Rink amide resin **S4** and intermediate **S13** was reacted with FITC to obtain resin-bound azide-containing intermediate **S14**, which was converted to resin-bound maleimide-thioester linker **S15** via CuAAC with **L2**. **b**, SPS of 5-carboxytetramethylrhodamine (5-TAMRA)-labeled Lock-PNA **SP1**. SPS was performed on TentaGel Rink amide resin **S4** to obtain resin-bound azide-containing oligomer **S16**, which was converted to resin-bound maleimide-thioester oligomer **S17** via CuAAC with **L2**. TFA cleavage and RP-HPLC purification afforded **SP1** and **SP2**.

Materials and Methods

General Information and Instrumentation

Abbreviations of chemicals

1-[Bis(dimethylamino)methylene]-1H-1,2,3-triazolo[4,5-b]pyridinium 3-oxide hexafluorophosphate (HATU), (7-azabenzotriazol-1-yloxy)trispyrrolidinophosphonium hexafluorophosphate (PyAOP), *N*-methylmaleimide (NMM) trifluoroacetic acid (TFA), *N*,*N*-dimethylformamide (DMF), tris(2-carboxyethyl)phosphine hydrochloride (TCEP·HCl), dimethyl sulfoxide (DMSO), 1,4-dithiothreitol (DTT), imidazole (Imd), isopropyl-beta-D-thiogalactopyranoside (IPTG), ethylenediaminetetraacetic acid (EDTA), 4-(2-hydroxyethyl)-1-piperazineethanesulfonic acid (HEPES), 5-carboxytetramethylrhodamine (5-TAMRA), fluorescein 5-isothiocyanate (FITC), 2-(*N*-morpholino)ethanesulfonic acid (MES), lithium dodecyl sulfate (LDS), benzhydryloxycarbonyl (Bhoc), fluorenylmethoxycarbonyl (Fmoc), trityl (Trt), polyethylene glycol (PEG), phosphate-buffered saline (PBS).

Chemistry reagents and materials

Coupling reagents and amino acid building blocks for solid-phase synthesis (SPS) were purchased from Fluorochem. TentaGel® resin was obtained from Iris Biotech. PNA building blocks for SPS were purchased from PNABio. All other chemicals were obtained in the highest available purity from Merck and used without further purification. General solvents were of technical grade and distilled prior to use. Anhydrous solvents for reactions were obtained by distillation over drying agents or purchased from Merck (DMF). Manual solid-phase synthesis was performed in polypropylene syringes equipped with fritted disks purchased from Torviq. Flash column chromatography was performed on silica gel 60 (particle size 35–70 μm) purchased from Merck. Thin-layer chromatography (TLC) was performed on silica gel plates (TLC Silicagel 60, Merck). Visualization was achieved either under UV light (254 nm) and/or by staining using an KMnO4 stain followed by heating with a heat gun.

Liquid chromatography–mass spectrometry (LC-MS)

LC–MS analyses were performed on a Waters SQ Detector 2 mass spectrometer coupled to an Acquity ultra-performance liquid chromatography (UPLC) system using an Acquity UPLC UPLC BEH300 C4 column (130 Å, 1.7 μm, 2.1 × 50 mm) for protein samples or an Acquity UPLC BEH C18 column (130 Å, 1.7 μm, 2.1 mm × 50 mm) for PNA samples. Gradients of eluent A (0.1% formic acid in water) and eluent B (0.1% formic acid in MeCN) at a flow rate of 0.2 mL min^{-1} were used. The electrospray ionization (ESI) source was operated with a capillary voltage of 3.0 kV and a cone voltage of 30 V. Nitrogen was used as the desolvation gas at a total flow of 800 L h^{-1}. Deconvolution of ion series of protein samples were performed using MassLynx software (v. 4.1 from Waters).

Preparative high-performance liquid chromatography (HPLC)

Preparative HPLC purifications were performed on a YMC-Triart C18 column (S-5 μm, 250 × 10.0 mm, 12 nm) using an Agilent 1260 Infinity II LC system equipped with a diode array UV detector. Gradients with eluent A (0.1% TFA in water) and eluent B (0.1% TFA in MeCN) at a flow rate of 8 mL min^{-1} were applied for purification. Fractions containing the purified target compound were identified using LC-MS. Selected fractions were pooled and lyophilized. Used gradients for purification are stated in the given experiment.

Nuclear magnetic resonance (NMR) spectroscopy

NMR spectra were recorded at 298 K using a Bruker Avance III HD 400 MHz (^{1}H NMR and ^{13}C NMR recorded at 400 and 101 MHz, respectively). Chemical shifts are reported in parts per million (ppm) relative to the deuterated solvent peak of $CDCl_3$ (δ_H = 7.26 ppm; δ_C = 77.16 ppm) as internal standard.

High-resolution mass spectrometry (HRMS)

High-resolution mass spectra were recorded by the Mass Spectrometry Service, Yusuf Hamied Department of Chemistry, University of Cambridge using a Waters LCT Premier or a Waters Xevo G2-S spectrometer and ionized by ESI or ASAP.

Protein expression reagents and materials

Chemically-competent *Escherichia coli* (*E. coli*) BL21 Star™ (DE3)pLysS One Shot™, IPTG (dioxane-free), kanamycin, chloramphenicol, super optimal broth with catabolite repression (S.O.C.) medium, Pierce™ protease inhibitor tablets and DNase I were purchased from Thermo Fisher Scientific. Codon optimized plasmids were purchased from Genscript. Ni Sepharose™ Excel resin was purchased from Merck. Recombinant His_6-tagged Tobacco Etch Virus (TEV) enzyme was obtained from Genscript Biotech. Molecular weight cut-off (MWCO) Amicon™ ultra centrifugal filters were purchased from Merck. Autoclaved lysogeny broth (LB) medium and agar plates were prepared in-house.

Sodium dodecyl sulfate–polyacrylamide gel (SDS-PAGE) electrophoreses

Protein purities were determined by SDS-PAGE using NuPAGE™ 4 to 12%, Bis-Tris, 1.0–1.5 mm, mini protein gels. Protein samples were loaded using NuPAGE™ lithium dodecyl sulfate (LDS) sample buffer (4x) and gels electrophoresis was performed using NuPAGE™ 2-(*N*-morpholino)ethanesulfonic acid (MES) running buffer with a voltage of 200 V for 35 min. Gel electrophoresis with sfGFP was performed on ice with a voltage of 120 V for 90 min. Coomassie staining was performed using InstantBlue™ Coomassie protein stain. SeeBlue™ Plus2 Pre-stained Protein Standard as was used reference. All materials were obtained from Thermo Fisher Scientific. Gels were visualized using a Bio-Rad ChemiDoc MP imaging system.

Protein desalting

Expressed protein desalting purifications were performed on a Cytiva HiPrep™ 26/10 Desalting column using a Cytiva ÄKTA pure™ chromatography system with a flow rate of 15.0 mL min^{-1} at room temperature and were monitored at a wavelength of λ = 280 nm. The applied mobile-phase was PBS (pH 7.4) if not otherwise stated.

Size-exclusion chromatography (SEC)

Expressed protein and protein bioconjugate purifications were performed by SEC on a Cytiva Superdex™ 75 Increase 10/300 GL column using a Cytiva ÄKTA pure™ chromatography system with a flow rate of 0.8 mL min^{-1} at room temperature and were monitored at a wavelength of λ = 280 nm. The applied mobile-phase was phosphate-buffered saline (PBS) (pH 7.4) if not otherwise stated.

Biolayer interferometry (BLI)

BLI measurements were performed on an Octet® K2 system (Pall ForteBio) using Octet® streptavidin-coated (SA) biosensors purchased from Sartorius and black 96-well plates with flat bottom and medium binding coating obtained from Greiner Bio-One (cat. 655076). The following proteins for BLI measurement were purchased from ACROBiosystems: biotinylated human IL-2Rβ (CD122) protein, His, Avitag™; (cat. ILB-H82E3), His-tagged human IL-2Rβ (CD122) (cat. CD2-H5221), biotinylated human IL-2Rγ (CD132) protein, His, Avitag™; (cat. ILG-H85E8) and biotinylated human CD40 protein, His, Avitag™ (cat. CB0-H82E8).

Nanodrop concentration determination

Concentrations of proteins were determined on a NanoDrop™ One^{C} microvolume UV-spectrophotometer (ThermoFisher Scientific) through the absorbance at λ = 280 nm (A280) applying the extinction coefficient calculated based on the protein sequence using the ProtParam tool (ExPASy). Concentrations of PNAs were determined through the absorbance at λ = 260 nm (A260) applying the extinction coefficient calculated based on the nucleobase sequence using the OligoAnalyzer™ tool (Integrated DNA Technologies; IDT).

Data handling and visualization

All assay data were analyzed using GraphPad Prism 9.3.1.

Chemical Synthesis of Thioester Linkers

***tert*-Butyl 4-(pent-4-ynoylthio)piperidine-1-carboxylate (S2)**

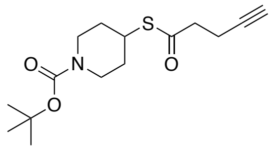

$C_{15}H_{23}NO_3S$
MW: 297.41

To a solution of pentynoic acid (339 mg, 3.45 mmol, 1.15 equiv), PyAOP (1.72 g, 3.30 mmol, 1.10 equiv) and anhydrous *i*-Pr_2NEt (1.57 mL, 9.00 mmol, 3.00 equiv) in anhydrous DMF (4.5 mL) was added a solution of 1-Boc-4-mercapto-piperidine **S1** (651 mg, 3.00 mmol, 1.00 equiv) in anhydrous DMF (1.5 mL). The reaction mixture was stirred for 60 min at room temperature and subsequently diluted with 90 mL EtOAc. The organic phase was washed with 1 M HCl (2 × 60 mL), saturated $NaHCO_3$ solution (2 × 60 mL) and brine (60 mL), filtered, dried over anhydrous Na_2SO_4 and concentrated to dryness under reduced pressure. The crude product was purified by flash column chromatography (SiO_2, EtOAc/petroleum ether = 1:4, R_f = 0.35) to afford the title compound **S2** as a white solid (819 mg, 2.75 mmol, 92%). **^{1}H NMR** (400 MHz, $CDCl_3$) δ = 3.93–3.77 (m, 2H), 3.76–3.57 (m, 1H), 3.23–2.96 (m, 2H), 2.76 (t, *J* = 7.3 Hz, 2H), 2.52 (td, *J* = 7.3, 2.7 Hz, 2H), 1.98 (t, *J* = 2.7 Hz, 1H), 1.95–1.81 (m, 2H), 1.65–1.47 (m, 2H), 1.44 (s, 9H). **^{13}C NMR** (101 MHz, $CDCl_3$) δ = 196.8, 154.7, 82.0, 79.8, 69.5, 42.6, 40.3, 32.0, 28.5, 14.7. **HRMS** (ESI) *m/z* calcd for $C_{15}H_{23}NO_3S$: $[M+H]^+$ 297.1393, found 297.1379.

4-(Pent-4-ynoylthio)piperidine-1-carboxylate 2,2,2-trifluoroacetate (S3)

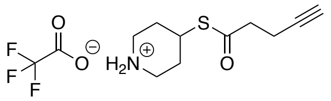

$C_{10}H_{15}NOS$
MW: 197.30

Boc-protected linker **S2** (297 mg, 1.00 mmol, 1.00 equiv) was dissolved in a mixture of CH_2Cl_2–TFA (2.50 mL, 4:1, v/v) and stirred at room temperature. After 1.0 h, the reaction mixture was concentrated under reduced pressure and remaining TFA was removed with repeated toluene co-evaporations under reduced pressure. The crude title compound **S3** (312 mg, 1.00 mmol, quant.) was used in the next step without further purification.

Thioester linker 1 (L1)

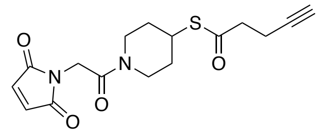

$C_{16}H_{18}N_2O_4S$
MW: 334.39

To a solution of 2-maleimidoacetic acid (155 mg, 1.00 mmol, 1.00 equiv), HATU (380 mg, 1.00 mmol, 1.00 equiv) and anhydrous *i*-Pr_2NEt (0.70 mL, 4.00 mmol, 4.00 equiv) in anhydrous DMF (2.0 mL) was added a solution of crude **S3** (312 mg, 1.00 mmol, 1.00 equiv) in anhydrous DMF (2.0 mL). The reaction mixture was stirred for 60 min at room temperature and subsequently diluted with 70 mL EtOAc. The organic phase was washed with 1 M HCl (2 × 50 mL), saturated $NaHCO_3$ solution (2 × 50 mL) and brine (50 mL), filtered, dried over anhydrous Na_2SO_4 and concentrated to dryness under reduced pressure. The crude product was purified by flash column chromatography (SiO_2, EtOAc/petroleum ether = 3:2, R_f = 0.4) to afford the title compound **L1** as a white solid (285 mg, 0.85 mmol, 85%). **^{1}H NMR** (400 MHz, $CDCl_3$) δ = 1.59 (qd, *J* = 10.1, 5.1 Hz, 1H), 1.67 (dtd, *J* = 13.8, 10.1, 3.9 Hz, 1H), 1.89–1.98 (m, 1H), 1.99 (t, *J* = 2.7 Hz, 1H), 2.02–2.14 (m, 1H), 2.46–2.61 (m, 2H), 2.77 (t, *J* = 7.2 Hz, 2H), 3.10 (ddd, *J* = 13.6, 10.1, 3.3 Hz, 1H), 3.31 (ddd, *J* = 13.6, 10.1, 3.1 Hz, 1H), 3.63–3.82 (m, 2H), 4.07–4.19 (m, 1H), 4.31 (s, 2H), 6.78 (s, 2H). **^{13}C NMR** (101 MHz, $CDCl_3$) δ = 196.5, 170.6, 163.8, 134.7, 81.9, 69.6, 44.4, 42.6, 42.1, 39.7, 39.0, 32.3, 31.3, 14.7. **HRMS** (ESI) *m/z* calcd for $C_{16}H_{19}N_2O_4S^+$: $[M+H]^+$ 335.1060, found 335.1048.

Thioester linker 2 (L2)

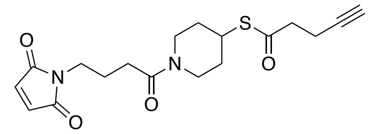

$C_{18}H_{22}N_2O_4S$
MW: 362.44

To a solution of 4-maleimidobutyric acid (80.5 mg, 0.44 mmol, 1.00 equiv), HATU (167 mg, 0.44 mmol, 1.00 equiv) and anhydrous *i*-Pr_2NEt (0.31 mL, 1.76 mmol, 4.00 equiv) in anhydrous DMF (1.0 mL) was added a solution of crude **S3** (135 mg, 0.44 mmol, 1.00 equiv) in anhydrous DMF (0.5 mL). The reaction mixture was stirred for 60 min at room temperature and subsequently diluted with 30 mL EtOAc. The organic phase was washed with 1 M HCl (2 × 20 mL), saturated $NaHCO_3$ solution (2 × 20 mL) and brine (20 mL), filtered, dried over anhydrous Na_2SO_4 and concentrated to dryness under reduced pressure. The crude product was purified by flash column chromatography (SiO_2, EtOAc/petroleum ether = 1:1, R_f = 0.35) to afford the title compound **L2** as a white solid (261 mg, 0.88 mmol, 88%). **^{1}H NMR** (400 MHz, $CDCl_3$) δ = 6.68 (s, 2H), 4.31–4.08 (m, 1H), 3.85–3.62 (m, 2H), 3.58 (t, *J* = 6.7 Hz, 2H), 3.37–2.99 (m, 2H), 2.76 (t, *J* = 7.3 Hz, 2H), 2.52 (td, *J* = 7.3, 2.6 Hz, 2H), 2.30 (t, *J* = 7.3 Hz, 2H), 1.99 (t, *J* = 2.6 Hz, 1H), 1.99–1.88 (m, 4H), 1.66–1.50 (m, 2H). **^{13}C NMR** (101 MHz, $CDCl_3$) δ = 196.7, 171.0, 170.0, 134.2, 81.9, 69.6, 42.6, 40.1, 37.6, 30.3, 24.2, 14.7. **HRMS** (ESI) *m/z* calcd for $C_{18}H_{23}N_2O_4S^+$: $[M+H]^+$ 363.1373, found 363.1359.

Synthesis of Peptide Nucleic Acids (PNAs)

General Procedures for Solid-Phase Synthesis (SPS) of PNAs

Building blocks used for Fmoc-based SPS

The following Fmoc-protected PNA building blocks (Fmoc-BB-OH) with acid-labile nucleobase protecting groups and Fmoc-protected amino acids were used for the synthesis of PNAs: Fmoc-PNA-A(Bhoc)-OH, Fmoc-PNA-C(Bhoc)-OH, Fmoc-PNA-G(Bhoc)-OH, Fmoc-PNA-T-OH, Fmoc-Arg(Pbf)-OH, Fmoc-Cys(Trt)-OH, Fmoc-Lys(N_3)-OH, Fmoc-Lys(Alloc)-OH, Fmoc-8-amino-3,6-dioxaoctanoic acid (Fmco-PEG2-OH).

General procedure for manual PNA SPS

Manual SPS was performed on 5.0 µmol or 10.0 µmol scale in polypropylene syringes equipped with fitted disks using Rink amide-loaded TentaGel® R resin **S4** (0.22 mmol/g). Resins were swelled for 15 min in DMF prior to SPS and PNAs were synthesized beginning from the C-terminal by the iteration of the following steps:

Coupling of a PNA or amino acid residue:

Fmoc-PNA-OH or Fmoc-AA-OH (5.00 equiv to the resin loading), HATU (4.90 equiv), and *N*-methylmorpholine (10.0 equiv) were pre-incubated in DMF (final concentration = 0.12 M for Fmoc-PNA-OH or Fmoc-AA-OH) for 2 min and then added to the resin. After 12 min under constant shaking at 45 °C, the coupling mixture was removed by suction and the resin was washed with DMF (3 × 45 s).

Fmoc deprotection:

The resin was treated with a solution of piperidine in DMF (1:4, v/v) (2 × 3 min) at room temperature and subsequently washed with DMF (5 × 45 s).

N-terminal acetylation:

The resin was treated with a solution of Ac_2O and *i*-Pr_2NEt in DMF (5:6:87, v/v/v) for 10 min at room temperature and subsequently washed with DMF (3 × 45 s) and CH_2Cl_2 (3 × 45 s), dried under vacuum.

N-terminal reaction with FITC:

FITC (5.00 equiv to the resin loading) was dissolved in anhydrous DMF (final concentration = 0.12 M) followed by the addition of anhydrous *i*-Pr_2NEt (10.0 equiv) and the mixture added to the resin. After 2 h under constant shaking at room temperature, the coupling mixture was removed by suction and the resin was washed with DMF (3 × 45 s) and CH_2Cl_2 (3 × 45 s), dried under vacuum.

General procedure for on-resin allyloxycarbonyl (Alloc) deprotection

After completed SPS elongation, PNA-peptidyl resin containing Lys(Alloc) residues was swelled in degassed CH_2Cl_2 (0.25 mL per 5.0 µmol of resin) under slight N_2 bubbling for 10 min at room temperature and PhSiH (24.0 equiv per Lys(Alloc) residue) was added to the resin followed by $Pd(PPh_3)_4$ (0.50 equiv per Lys(Alloc) residue). The reaction was allowed to proceed for 20 min under slight N_2 bubbling and the reaction mixture was subsequently removed by suction, the resin washed with CH_2Cl_2 (3 × 45 s) and the previous procedure repeated. After 20 min reaction time, the resin was washed with CH_2Cl_2 (3 × 45 s) and DMF (5 × 45 s) and the Lys *ε*-amines were available for subsequent coupling steps.

General procedure for on-resin copper-catalyzed azide–alkyne cycloadditions (CuAAC)

After completed SPS elongation, PNA-peptidyl resin containing Lys(N_3) residues was swelled in anhydrous DMF (0.25 mL per 5.0 μmol of resin) for 10 min under N_2 atmosphere and the solvent was subsequently removed. To a solution of CuI (2.00 equiv per azide) and anhydrous *i*-Pr_2NEt (25.0 equiv per azide) in anhydrous DMF (0.5 mL per 5.0 μmol of resin) was added thioester linker **L1** or **L2** (5.00 equiv per azide) and the reaction mixture was added to the resin under N_2 atmosphere. The resin was agitated for 60 min at room temperature and the reaction mixture was subsequently removed by suction, the resin was washed with DMF (5 × 45 s) and CH_2Cl_2 (3 × 45 s) and dried under vacuum.

General procedure for TFA-mediated cleavage of PNAs

TFA-mediated cleavage of PNAs from the solid support and global deprotection was performed in a polypropylene syringe equipped with a fritted disk by treating a vacuum-dried PNA-peptidyl-resin with a deprotection/cleavage cocktail [TFA–*i*-Pr_3SiH–water (95:2.5:2.5, v/v/v)] (1.0 mL per 5.0 μmol of resin) for 2 h. After complete cleavage, the PNA containing cleavage solution was collected in a centrifugal tube and the resin rinsed with neat TFA (0.5 mL per 5.0 μmol of resin). The combined TFA cleavage solutions were concentrated under a stream of N_2 to approx. 0.5 mL and cold Et_2O (–20 °C) (10 mL) was added to precipitate the crude PNAs. The crude PNAs were pelleted by centrifuging (2000 rpm) for 5 min and the Et_2O supernatant was decanted. The crude PNAs were dried under a stream of N_2 and purified by RP-HPLC.

Synthesized Lock-PNAs

Lock-PNA-L1 10nt (P1)

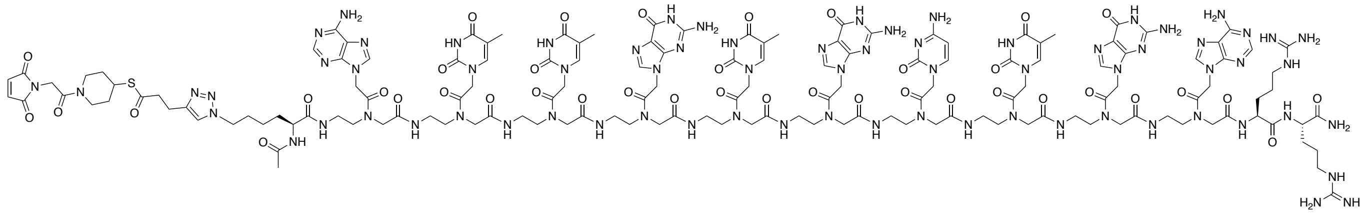

The thioester-containing Lock-PNA (**P1**) (SPS: Ac-Lys(N_3)-ATTGTGCTGA-Arg-Arg) was synthesized using the general procedures for manual SPS (10.0 μmol), on-resin CuAAC (2.5 μmol) using linker **L1** and TFA-mediated cleavage. Preparative RP-HPLC purification (0–30% B over 30 min) afforded PNA **P1** as a white solid (2.3 mg, 0.6 μmol, 24%). Purity >95% determined by LC-MS (λ = 260 nm). **LC-MS** (ESI) *m/z* calcd for $C_{145}H_{191}N_{71}O_{40}S$: $[M+2H]^{2+}$: 1801.3, found 1801.3; $[M+3H]^{3+}$: 1201.2, found 1201.3; $[M+4H]^{4+}$: 901.2, found 901.3; $[M+5H]^{5+}$: 721.1, found 721.2. **HRMS** (ESI) mass calcd for $C_{145}H_{191}N_{71}O_{40}S$: [M] 3598.4815, deconvoluted mass found 3598.5151.

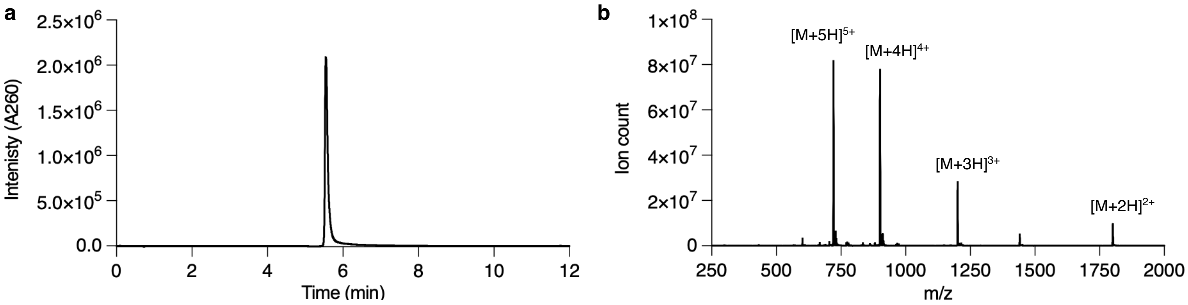

Supporting Figure S12. a, UV trace of purified PNA **P1** (λ = 260 nm). **b**, ESI mass spectrum of PNA **P1**.

Lock-PNA-L2 10nt (P2)

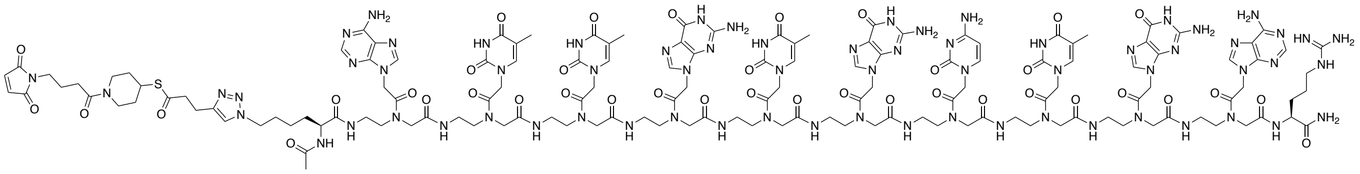

The thioester-containing Lock-PNA (**P2**) (SPS: Ac-Lys(N_3)-ATTGTGCTGA-Arg) was synthesized using the general procedures for manual SPS (10.0 μmol), on-resin CuAAC (10.0 μmol) using linker **L2** and TFA-mediated cleavage. Preparative RP-HPLC purification (0–30% B over 30 min) afforded PNA **P2** as a white solid (7.3 mg, 2.1 μmol, 21%). Purity >95% determined by LC-MS (λ = 260 nm). **LC-MS** (ESI) *m/z* calcd for $C_{141}H_{183}N_{67}O_{39}S$: $[M+2H]^{2+}$: 1737.3, found 1737.6; $[M+3H]^{3+}$: 1158.5, found 1158.6; $[M+4H]^{4+}$: 869.1, found 891.4; $[M+5H]^{5+}$: 695.5, found 695.7. **HRMS** (ESI) mass calcd for $C_{141}H_{183}N_{67}O_{39}S$: [M] 3470.4117, deconvoluted mass found 3470.9329.

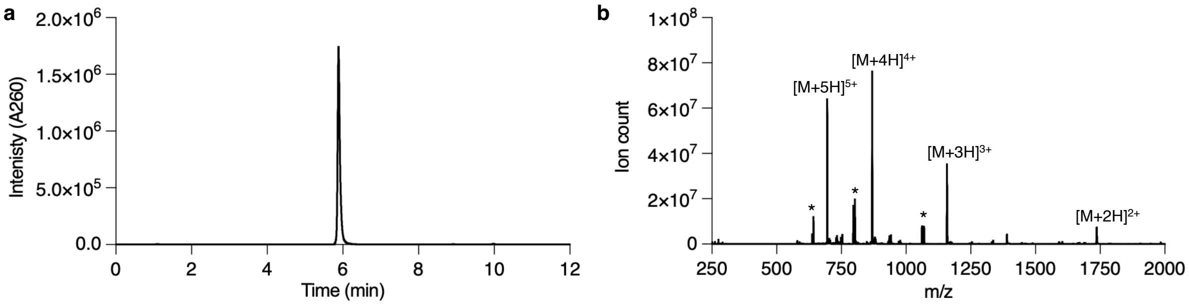

Supporting Figure S13. a, UV trace of purified PNA **P2** (λ = 260 nm). **b**, ESI mass spectrum of PNA **P2**. *Guanine deletion (M–291 Da).

Bis-Lock-PNA-L1 6nt (P4)

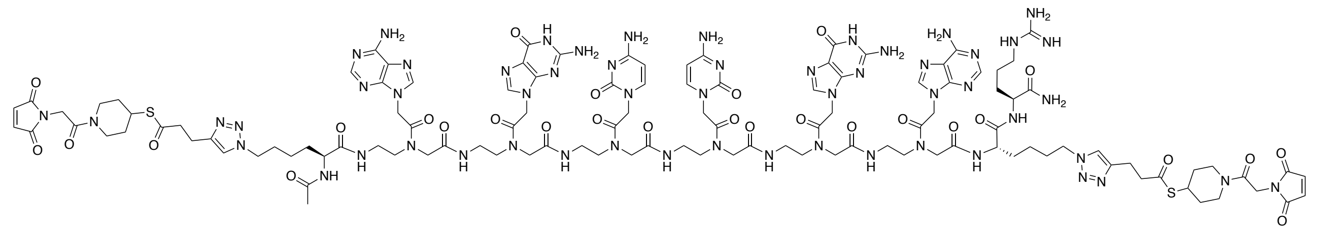

The thioester-containing bis-Lock-PNA (**P4**) (SPS: Ac-Lys(N_3)-AGCCGA-Lys(N_3)-Arg) was synthesized using the general procedures for manual SPS (20.0 μmol), on-resin CuAAC (20.0 μmol) using linker **L1** and TFA-mediated cleavage. Preparative RP-HPLC purification (0–30% B over 30 min) afforded PNA **P4** as a white solid (17.8 mg,

6.3 µmol, 31%). Purity >95% determined by LC-MS (λ = 260 nm). **LC-MS** (ESI) *m/z* calcd for $C_{116}H_{151}N_{55}O_{28}S_2$: $[M+2H]^{2+}$: 1415.0, found 1415.0; $[M+3H]^{3+}$: 943.7, found 943.7; $[M+4H]^{4+}$: 708.0, found 708.1; $[M+5H]^{5+}$: 566.6, found 566.7. **HRMS** (ESI) mass calcd for $C_{116}H_{151}N_{55}O_{28}S_2$: [M] 2826.1524, deconvoluted mass found 2826.6794.

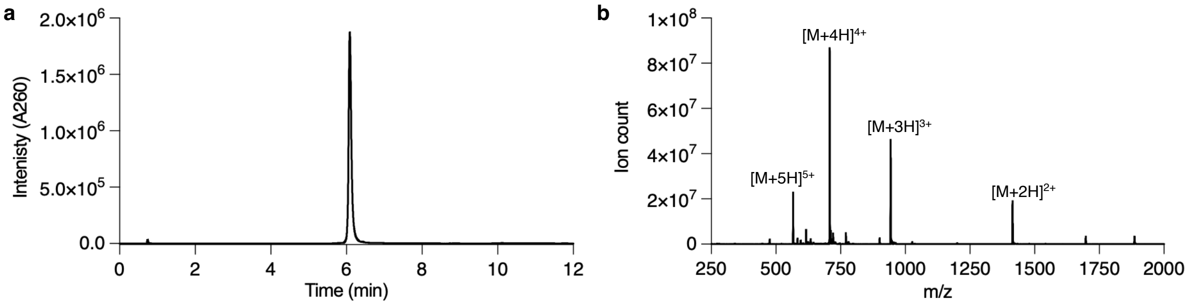

Supporting Figure S14. **a**, UV trace of purified PNA **P4** (λ = 260 nm). **b**, ESI mass spectrum of PNA **P4**.

Bis-Lock-PNA-L2 6nt (P5)

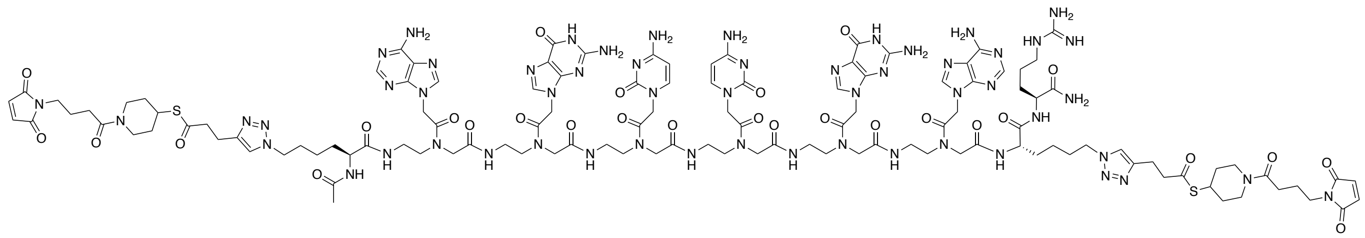

The thioester-containing bis-Lock-PNA (**P5**) (SPS: Ac-Lys(N_3)-AGCCGA-Lys(N_3)-Arg) was synthesized using the general procedures for manual SPS (10.0 µmol), on-resin CuAAC (5.0 µmol) using linker **L2** and TFA-mediated cleavage. Preparative RP-HPLC purification (0–30% B over 30 min) afforded PNA **P5** as a white solid (2.8 mg, 1.0 µmol, 20%). Purity >95% determined by LC-MS (λ = 260 nm). **LC-MS** (ESI) *m/z* calcd for $C_{120}H_{159}N_{55}O_{28}S_2$: $[M+2H]^{2+}$: 1443.0, found 1443.2; $[M+3H]^{3+}$: 962.4, found 962.5; $[M+4H]^{4+}$: 722.0, found 722.3; $[M+5H]^{5+}$: 577.8, found 580.0. **HRMS** (ESI) mass calcd for $C_{120}H_{159}N_{55}O_{28}S_2$: [M] 2882.2150, deconvoluted mass found 2882.7605.

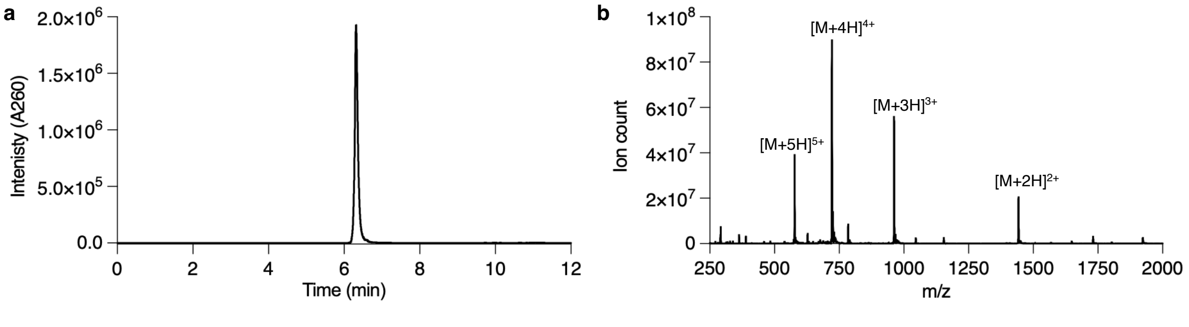

Supporting Figure S15. **a**, UV trace of purified PNA **P5** (λ = 260 nm). **b**, ESI mass spectrum of PNA **P5**.

Synthesized Key-PNAs

Key-PNA 10nt (P3)

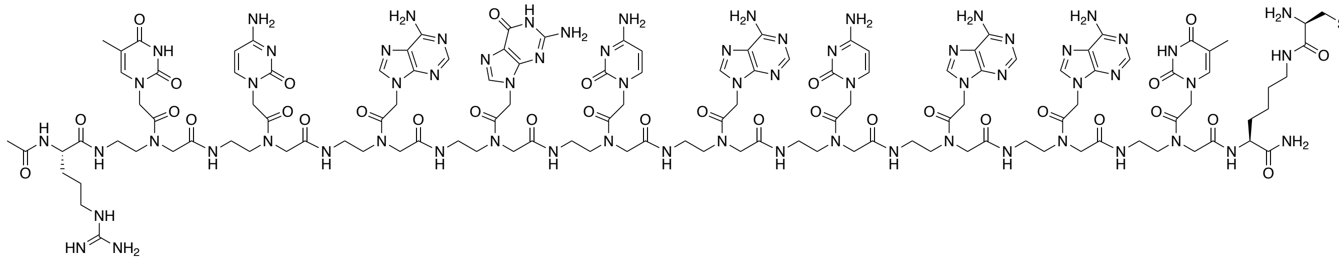

The cysteine-containing Key-PNA (**P3**) (SPS: Ac-Arg-TCAGCACAAT-Lys(Alloc)) was synthesized using the general procedures for manual SPS (10.0 µmol), on-resin Alloc deprotection (10.0 µmol) with subsequent coupling of Fmoc-Cys(Trt)-OH and TFA-mediated cleavage. Preparative RP-HPLC purification (0–20% B over 30 min) afforded PNA **P3** as a white solid (8.3 mg, 2.7 µmol, 27%). Purity 83% determined by LC-MS (λ = 260 nm). **LC-MS** (ESI) *m/z* calcd for $C_{124}H_{166}N_{66}O_{32}S$: $[M+2H]^{2+}$: 1563.6, found 1564.3; $[M+3H]^{3+}$: 1042.7, found 1043.2; $[M+4H]^{4+}$: 782.3, found 782.4; $[M+5H]^{5+}$: 626.0, found 626.2; $[M+6H]^{6+}$: 521.9, found 522.2; $[M+7H]^{7+}$: 447.5, found 447.7. **HRMS** (ESI) mass calcd for $C_{124}H_{166}N_{66}O_{32}S$: [M] 3123.3112, deconvoluted mass found 3123.8230.

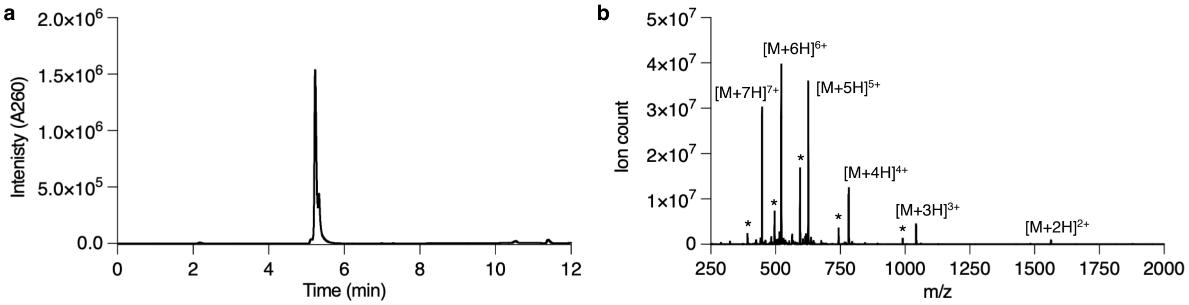

Supporting Figure S16. a, UV trace of purified PNA **P3** (λ = 260 nm). **b**, ESI mass spectrum of PNA **P3**. *Arginine deletion (M–156 Da)

Bis-Key-PNA 6nt (P6)

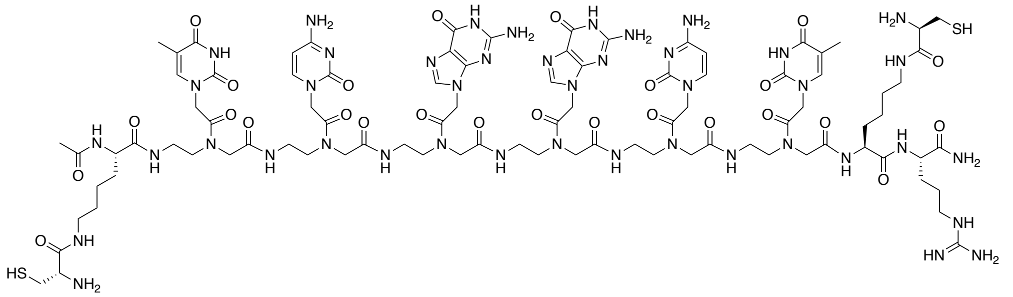

The cysteine-containing bis-Key-PNA (**P6**) (SPS: Ac-Lys(Alloc)-TCGGCT-Lys(Alloc)-Arg) was synthesized using the general procedures for manual SPS (10.0 µmol), on-resin Alloc deprotection (10.0 µmol) with subsequent coupling of Fmoc-Cys(Trt)-OH and TFA-mediated cleavage. Preparative RP-HPLC purification (0–20% B over 30 min) afforded PNA **P6** as a white solid (7.4 mg, 3.2 µmol, 32%). Purity >95% determined by LC-MS (λ = 260 nm). **LC-MS** (ESI) *m/z* calcd for $C_{90}H_{131}N_{43}O_{26}S_2$: $[M+2H]^{2+}$: 1148.7, found 1149.0; $[M+3H]^{3+}$: 766.2,

found 766.4; $[M+4H]^{4+}$: 574.9, found 575.2. **HRMS** (ESI) mass calcd for $C_{90}H_{131}N_{43}O_{26}S_2$: [M] 2293.9692, deconvoluted mass found 2292.9636.

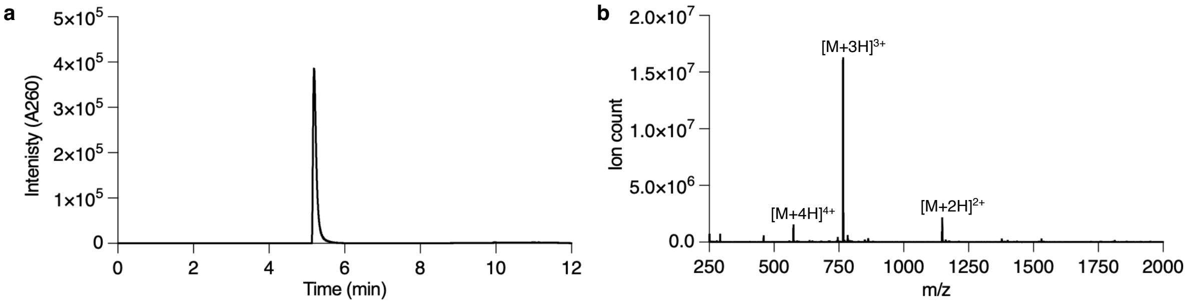

Supporting Figure S17. a, UV trace of purified PNA **P6** (λ = 260 nm). **b**, ESI mass spectrum of PNA **P6**.

Fluorophore-Labelled Reagents

FITC-PEG-Linker L2 (SP1)

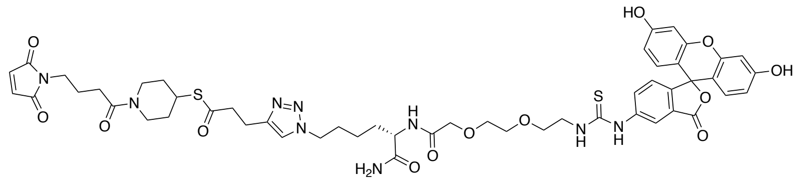

The FITC-labelled linker (**SP1**) (SPS: FITC-PEG2-Lys(N_3)) was synthesized using the general procedures for manual SPS (10.0 µmol), on-resin CuAAC (10.0 µmol) using linker **L2** and TFA-mediated cleavage. Preparative RP-HPLC purification (0–30% B over 30 min) afforded linker **SP1** as a yellow solid (3.2 mg, 3.0 µmol, 30%). Purity >95% determined by LC-MS (λ = 215 nm). **LC-MS** (ESI) *m/z* calcd for $C_{51}H_{57}N_9O_{13}S_2$: $[M+H]^+$: 1069.2, found 1068.6; $[M+2H]^{2+}$: 535.1, found 535.2. **HRMS** (ESI) *m/z* calcd for $C_{16}H_{19}NaN_2O_4S^+$: $[M+Na]^+$ 1090.3409, found 1090.3418.

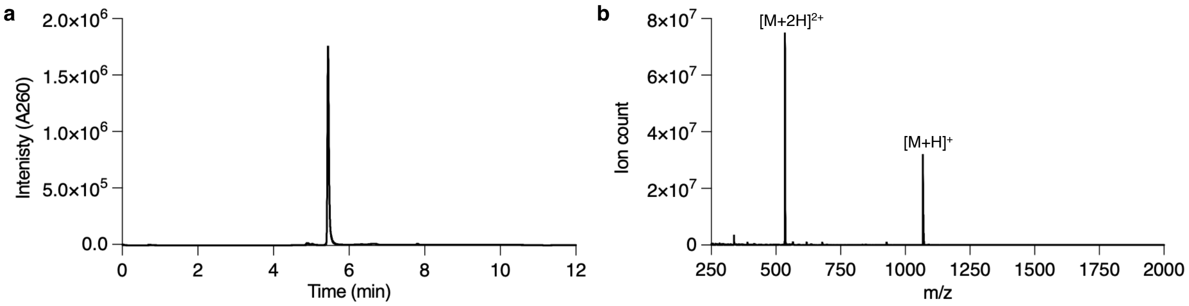

Supporting Figure S18. a, UV trace of purified linker **SP1** (λ = 215 nm). **b**, ESI mass spectrum of linker **SP1**.

TAMRA-Lock-PNA 10nt (SP2)

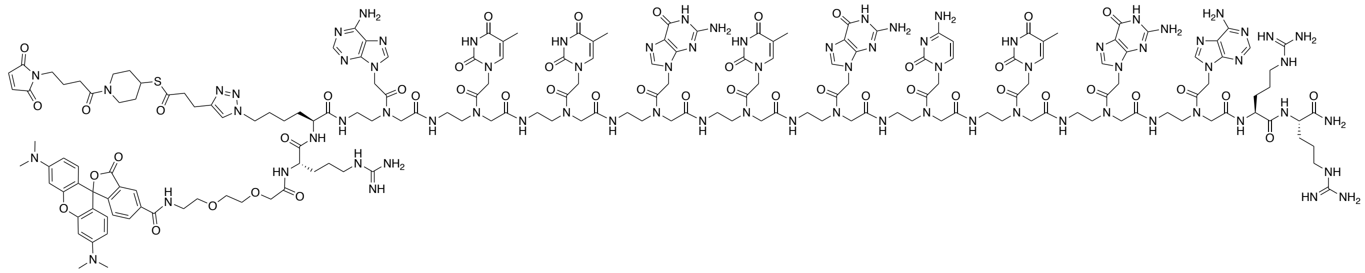

The thioester-containing Lock-PNA (**SP2**) (SPS: TAMRA-PEG2-Arg-Lys(N_3)-<u>ATTGTGCTGA</u>-Arg-Arg) was synthesized using the general procedures for manual SPS (10.0 µmol) with exception of the last coupling step, which was performed on 2.5 µmol resin with 2.0 equiv of 5-TAMRA, on-resin CuAAC (2.5 µmol) using linker **L2** and TFA-mediated cleavage. Preparative RP-HPLC purification (0–30% B over 30 min) afforded PNA **SP2** as a purple solid (1.2 mg, 0.3 µmol, 11%). Purity >95% determined by LC-MS (λ = 260 nm). **LC-MS** (ESI) *m/z* calcd for $C_{182}H_{236}N_{78}O_{47}S$: $[M+4H]^{4+}$: 1076.1, found 1076.7; $[M+5H]^{5+}$: 861.1, found 861.4; $[M+6H]^{6+}$: 717.8, found 717.9; $[M+7H]^{7+}$: 615.4, found 615.6. **HRMS** (ESI) mass calcd for $C_{182}H_{236}N_{78}O_{47}S$: [M] 4297.8195, deconvoluted mass found 4298.8555.

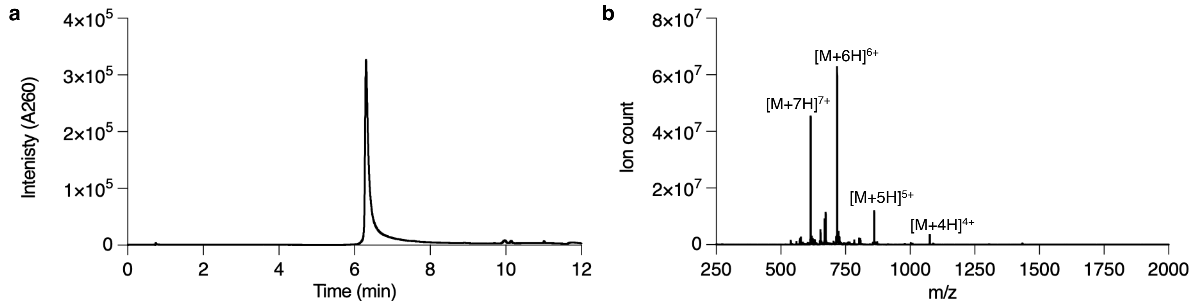

Supporting Figure S19. a, UPLC trace of purified PNA **SP2** (λ = 260 nm). **b**, ESI mass spectrum of PNA **SP2**.

Protein Expression in *E. coli*

Transformation of *E. coli*

Plasmids vectors (pET-30a(+)) containing the desired proteins sequences including a N-terminal His_6-tag, and a TEV cleavage site for neoleukin-2/15 (Neo-2/15) proteins, were used to transform chemically competent *E. coli* BL21 (DE3)pLysS. Plasmids solutions (1.0 µL, concentration 10 ng/µL) were added to freshly thawed bacterial aliquots (50 µL) on ice and after 5 min, the bacterial aliquots were placed in a water bath at 42 °C. After 30 s, the aliquots were placed on ice for 2 min followed by the addition of 0.25 mL of room temperature S.O.C. medium. The bacterial cultures were then grown in a shaking incubator at 37 °C and 200 rpm for 1 h and subsequently spread on LB agar plates containing kanamycin (50 µg/mL) and chloramphenicol (34 µg/mL) and incubated at 37 °C overnight. Overnight cultures from single colonies were grown in LB medium containing kanamycin (50 µg/mL) and chloramphenicol (34 µg/mL) and used to prepare bacterial stocks in 50% glycerol, which were stored at –80 °C.

Cell growth and purification

Overnight cultures of transformed *E. coli* grown at 37 °C in LB medium containing kanamycin (50 µg/mL) and chloramphenicol (34 µg/mL) were added to LB medium (25 mL of ON culture per 1.0 L) containing kanamycin (50 µg/mL) and the bacterial cultures were grown in a shaking incubator at 200 rpm and 37 °C. Once the optical dentistry at 600 nm (OD600) of the bacterial cultures reached ~0.6, a solution of IPTG in sterile water (1.0 M, 1.0 mL per 1.0 L of medium, final concentration: 1.0 mM) was added to induce protein expression and the induced cultures were incubated overnight at 200 rpm and 20 °C. The next day, the bacterial cells were pelleted by centrifugation at 8000 rpm for 30 °min and the pellets resuspended in lysis buffer (20 mL per 1.0 L of medium) (PBS buffer containing 500 mM NaCl, Pierce™ protease inhibitor tablet, 1 mg DNase and 1.0 mM DTT). Cells were lysed by sonication for 5 min (60% power amplitude, 20 s on/40 s off cycles) and the lysate clarified by centrifugation at 18000 rpm for 30 min. The supernatant was loaded onto Ni Sepharose™ Excel resin (2.0 mL resin per 1.0 L of culture) pre-equilibrated with PBS (pH 7.4) at room temperature on a rolling shaker. After 60 min, the resin was separated from the clarified lysate and washed with 20 mM imidazole in PBS (pH 7.4) (10 mL per 1.0 L of culture) and 40 mM imidazole in PBS (pH 7.4) (10 mL per 1.0 L of culture) and the protein eluted with 500 mM imidazole in PBS (pH 7.4) (10 mL per 1.0 L of culture). The eluted protein fraction was desalted using a Cytiva HiPrep™ 26/10 Desalting column on a Cytiva ÄKTA pure™ chromatography system with PBS (pH 7.4) as mobile phase.

TEV cleavage and SEC purification of proteins

His_6-tagged proteins were diluted to 1.5 mg/mL with PBS (7.4) and solutions of DTT in water (100 mM) and EDTA in water (50 mM) were added to reach final concentrations of 1.0 mM DTT and 0.5 mM EDTA. His_6-tagged TEV protease (1 IU per 10 µg of His_6-tagged protein) was added and the enzymatic reaction was allowed to proceed at 30 C for 2.5 h without shaking. Reaction progress was controlled by LC-MS analysis and if full conversion of the His_6-tagged protein was observed, the solution was loaded onto Ni Sepharose™ Excel resin (2.0 mL resin) pre-equilibrated with PBS (pH 7.4) at room temperature for 60 min. The resin was subsequently separated from the protein solution, washed with 10 mL PBS (pH 7.4), the washing was combined with protein solution and the combined solution was concentrated using Amicon™ ultra centrifugal filters (MWCO: 3 kDa) to ~6.0 mL. The concentrated protein solution was purified by SEC chromatography using a Cytiva Superdex™ 75 Increase 10/300 GL column on a Cytiva ÄKTA pure™ chromatography system and purified protein aliquots were immediately frozen in liquid N_2 and stored at –80 °C to avoid oxidation of cysteine residues.

Characterization of Recombinant Proteins

Neoleukin-2/15 (Neo-2/15) (WT)

Sequence:	MGHHHHHHGSS**ENLYFQ***GSSPKKKIQLHAEHALYDALMILNIVKTNSPPAEEKLEDYAFNFE LILEEIARLFESGDQKDEAEKAKRMKEWMKRIKTTASEDEQEEMANAIITILQSWIFS
MW (Da):	13952 (120 AA, with His_6-tag)<br>11915 (103 AA, after *TEV site cleavage)
Yield:	16 mg/L after TEV treatment and SEC purification

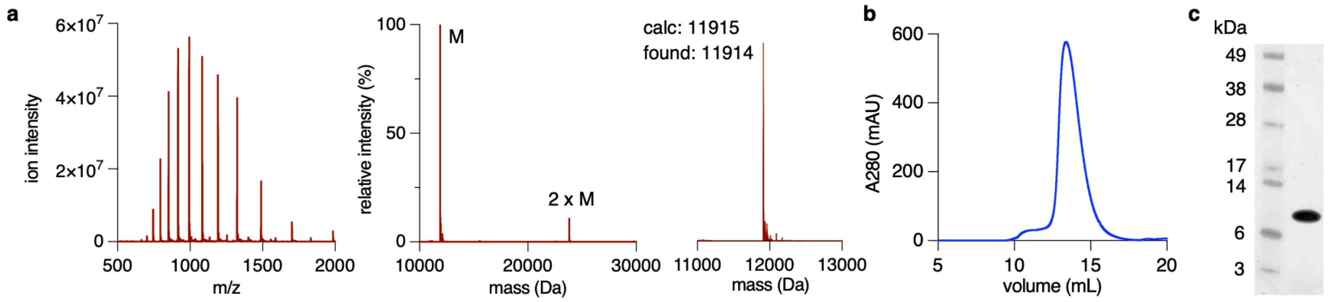

Supporting Figure S20. a, Combined ion series and deconvoluted mass spectra of **WT**. **b**, Chromatogram of SEC purification of **WT** after TEV treatment. **c**, SDS-PAGE gel of purified **WT** stained with Coomassie Blue.

Neo-2/15 H8C mutant (H8C)

Sequence:	MGHHHHHHGSS**ENLYFQ***GSSPKKKIQLCAEHALYDALMILNIVKTNSPPAEEKLEDYAFNFE LILEEIARLFESGDQKDEAEKAKRMKEWMKRIKTTASEDEQEEMANAIITILQSWIFS
MW (Da):	13918 (120 AA, with His_6-tag)<br>11881 (103 AA, after *TEV cleavage site)
Yield:	13 mg/L after TEV treatment and SEC purification

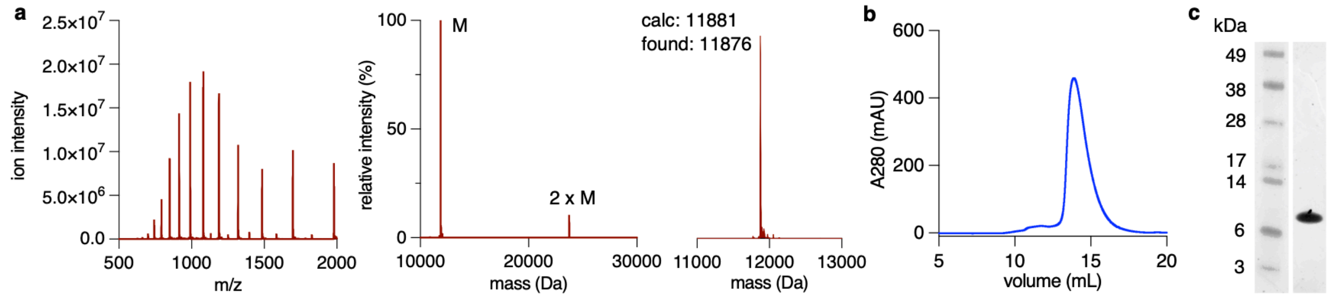

Supporting Figure S21. a, Combined ion series and deconvoluted mass spectra of **H8C**. **b**, Chromatogram of SEC purification of **H8C** after TEV treatment. **c**, SDS-PAGE gel of purified **H8C** stained with Coomassie Blue.

Neo-2/15 H11C mutant (H11C)

Sequence:	MGHHHHHHGSS**ENLYFQ***GSSPKKKIQLHAECALYDALMILNIVKTNSPPAEEKLEDYAFNFE LILEEIARLFESGDQKDEAEKAKRMKEWMKRIKTTASEDEQEEMANAIITILQSWIFS
MW (Da):	13918 (120 AA, with His_6-tag)<br>11881 (103 AA, after *TEV cleavage site)
Yield:	16 mg/L after TEV treatment and SEC purification

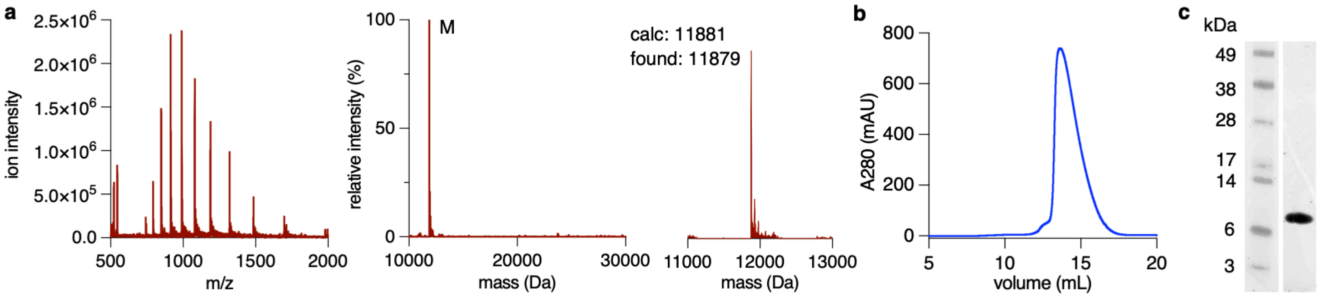

Supporting Figure S22. a, Combined ion series and deconvoluted mass spectra of **H11C**. **b**, Chromatogram of SEC purification of **H11C** after TEV treatment. **c**, SDS-PAGE gel of purified **H11C** stained with Coomassie Blue.

Neo-2/15 Y14C mutant (Y14C)

Sequence:	MGHHHHHHGSS**ENLYFQ***GSSPKKKIQLHAEHALCDALMILNIVKTNSPPAEEKLEDYAFNFE LILEEIARLFESGDQKDEAEKAKRMKEWMKRIKTTASEDEQEEMANAIITILQSWIFS
MW (Da):	13892 (120 AA, with His_6-tag)<br>11855 (103 AA, after *TEV cleavage site)
Yield:	16 mg/L after TEV treatment and SEC purification

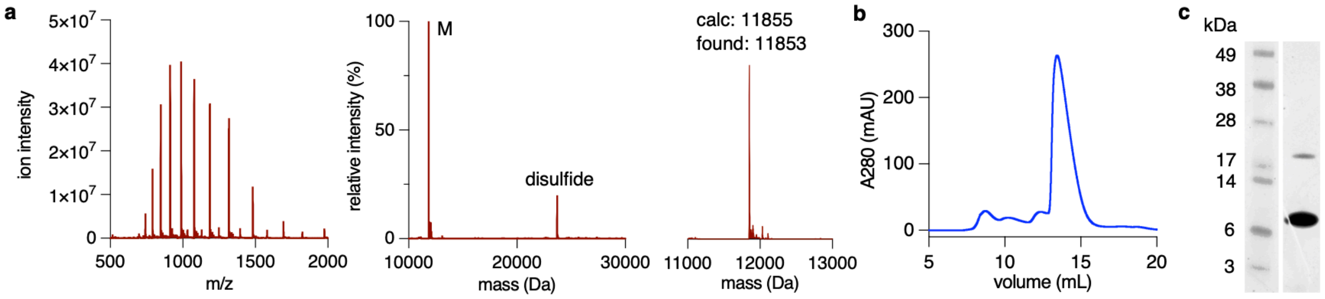

Supporting Figure S23. a, Combined ion series and deconvoluted mass spectra of **Y14C**. **b**, Chromatogram of SEC purification of **Y14C** after TEV treatment. **c**, SDS-PAGE gel of purified **Y14C** stained with Coomassie Blue.

Neo-2/15 D15C mutant (D15C)

Sequence:	MGHHHHHHGSS**ENLYFQ***GSSPKKKIQLHAEHALYCALMILNIVKTNSPPAEEKLEDYAFNFE LILEEIARLFESGDQKDEAEKAKRMKEWMKRIKTTASEDEQEEMANAIITILQSWIFS
MW (Da):	13940 (120 AA, with His$_6$-tag)<br>11903 (103 AA, after *TEV cleavage site)
Yield:	15 mg/L after TEV treatment and SEC purification

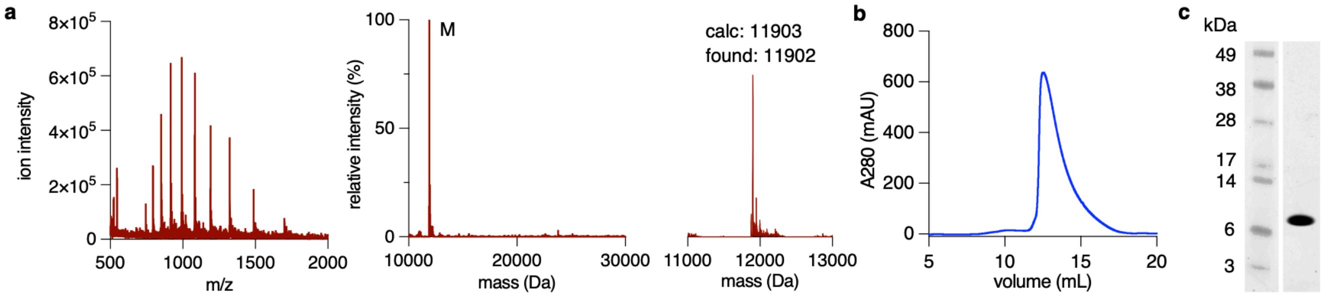

Supporting Figure S24. a, Combined ion series and deconvoluted mass spectra of **D15C**. **b**, Chromatogram of SEC purification of **D15C** after TEV treatment. **c**, SDS-PAGE gel of purified **D15C** stained with Coomassie Blue.

Neo-2/15 K33C mutant (K33C)

Sequence:	MGHHHHHHGSS**ENLYFQ***GSSPKKKIQLHAEHALYDALMILNIVKTNSPPAEECLEDYAFNFE LILEEIARLFESGDQKDEAEKAKRMKEWMKRIKTTASEDEQEEMANAIITILQSWIFS
MW (Da):	13927 (120 AA, with His$_6$-tag)<br>11890 (103 AA, after *TEV cleavage site)
Yield:	12 mg/L after TEV treatment and SEC purification

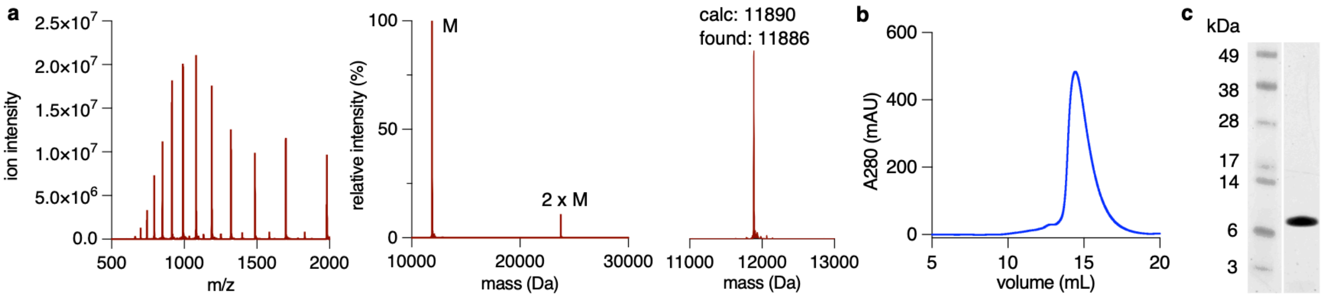

Supporting Figure S25. a, Combined ion series and deconvoluted mass spectra of **K33C**. **b**, Chromatogram of SEC purification of **K33C** after TEV treatment. **c**, SDS-PAGE gel of purified **K33C** stained with Coomassie Blue.

Neo-2/15 L7C K33C double mutant (L7C K33C)

Sequence:	MGHHHHHHGSS**ENLYFQ***GSSPKKKIQCHAEHALYDALMILNIVKTNSPPAEECLEDYAFNFE LILEEIARLFESGDQKDEAEKAKRMKEWMKRIKTTASEDEQEEMANAIITILQSWIFS
MW (Da):	13917 (120 AA, with His_6-tag)<br>11880 (103 AA, after *TEV cleavage site)
Yield:	14 mg/L after TEV treatment and SEC purification

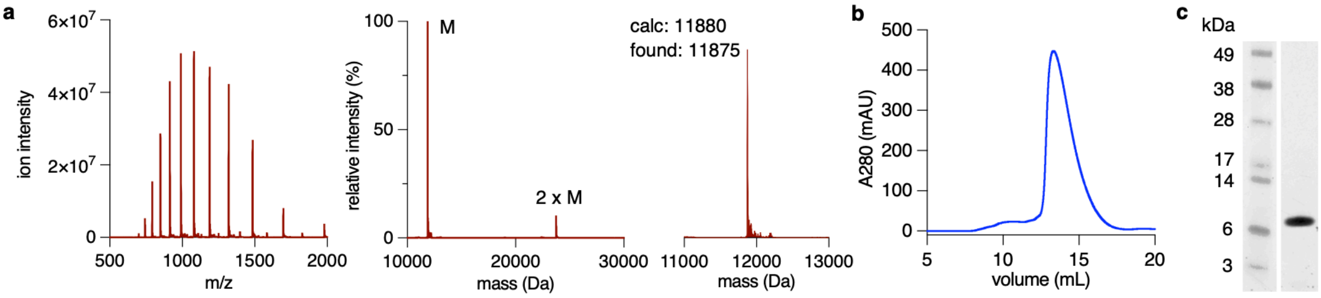

Supporting Figure S26. a, Combined ion series and deconvoluted mass spectra of **L7C K33C**. **b**, Chromatogram of SEC purification of **L7C K33C** after TEV treatment. **c**, SDS-PAGE gel of purified **L7C K33C** stained with Coomassie Blue.

Neo-2/15 Y14C K33C double mutant (Y14C K33C)

Sequence:	MGHHHHHHGSS**ENLYFQ***GSSPKKKIQLHAEHALCDALMILNIVKTNSPPAEECLEDYAFNFE LILEEIARLFESGDQKDEAEKAKRMKEWMKRIKTTASEDEQEEMANAIITILQSWIFS
MW (Da):	13867 (120 AA, with His_6-tag)<br>11830 (103 AA, after *TEV cleavage site)
Yield:	19 mg/L after TEV treatment and SEC purification

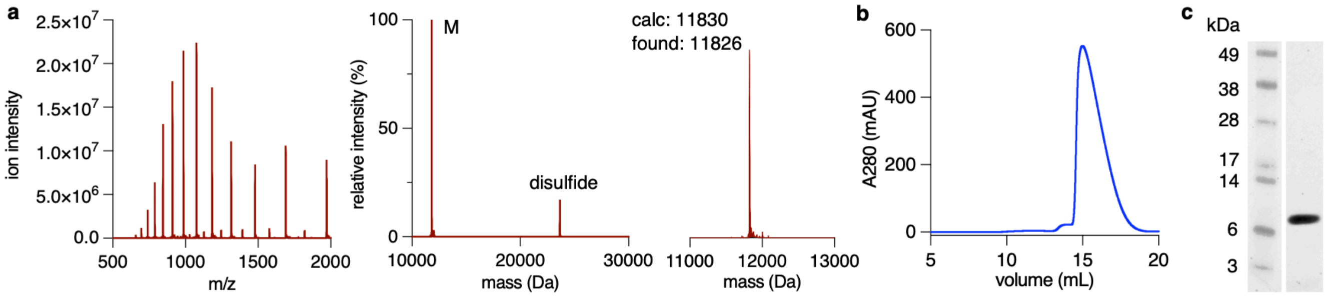

Supporting Figure S27. a, Combined ion series and deconvoluted mass spectra of **Y14C K33C**. **b**, Chromatogram of SEC purification of **Y14C K33C** after TEV treatment. **c**, SDS-PAGE gel of purified **Y14C K33C** stained with Coomassie Blue.

Neo-2/15 K33C E47C double mutant (K33C E47C)

Sequence: MGHHHHHHGSS**ENLYFQ***GSSPKKKIQLHAEHALYDALMILNIVKTNSPPAEECLEDYAFNFE
LILECIARLFESGDQKDEAEKAKRMKEWMKRIKTTASEDEQEEMANAIITILQSWIFS

MW (Da): 13901 (120 AA, with His_6-tag)
11864 (103 AA, after *TEV cleavage site)

Yield: 9 mg/L after TEV treatment and SEC purification

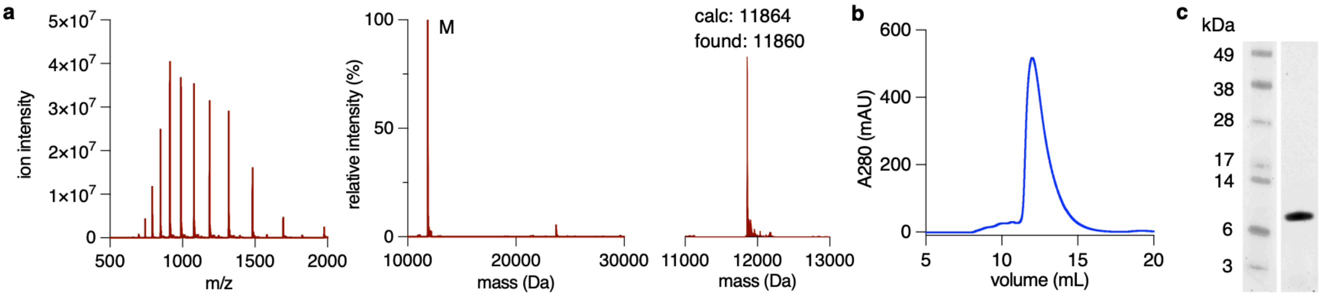

Supporting Figure S28. **a**, Combined ion series and deconvoluted mass spectra of **K33C E47C**. **b**, Chromatogram of SEC purification of **K33C E47C** after TEV treatment. **c**, SDS-PAGE gel of purified **K33C E47C** stained with Coomassie Blue.

Neo-2/15 K33C L51C double mutant (K33C L51C)

Sequence: MGHHHHHHGSS**ENLYFQ***GSSPKKKIQLHAEHALYDALMILNIVKTNSPPAEECLEDYAFNFE
LILEEIARCFESGDQKDEAEKAKRMKEWMKRIKTTASEDEQEEMANAIITILQSWIFS

MW (Da): 13917 (120 AA, with His_6-tag)
11880 (103 AA, after *TEV cleavage site)

Yield: 19 mg/L after TEV treatment and SEC purification

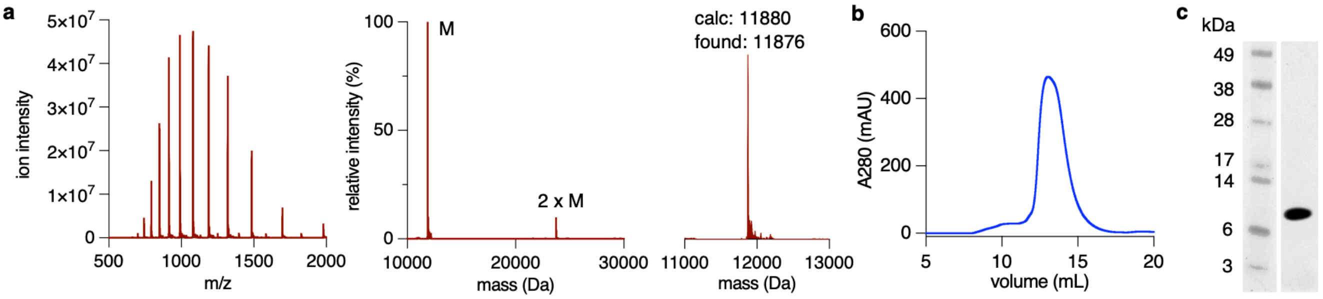

Supporting Figure S29. **a**, Combined ion series and deconvoluted mass spectra of **K33C L51C**. **b**, Chromatogram of SEC purification of **K33C L51C** after TEV treatment. **c**, SDS-PAGE gel of purified **K33C L51C** stained with Coomassie Blue.

Superfolder green fluorescent protein S147C mutant (sfGFP S147C)

Sequence:	MRSGHHHHHHGSS**ENLYFQ***GSSMRKGEELFTGVVPILVELDGDVNGHKFSVRGEGEGDATNG<br>KLTLKFICTTGKLPVPWPTLVTTLTYGVQCFARYPDHMKQHDFFKSAMPEGYVQERTISFKDD<br>GTYKTRAEVKFEGDTLVNRIELKGIDFKEDGNILGHKLEYNFNCHNVYITADKQKNGIKANFK<br>IRHNVEDGSVQLADHYQQNTPIGDGPVLLPDNHYLSTQSVLSKDPNEKRDHMVLLEFVTAAGI<br>THGMDELYK
MW (Da):	29334 (260 AA, with His_6-tag)<br>27054 (241 AA, after *TEV cleavage site)
Yield:	29 mg/L after SEC purification

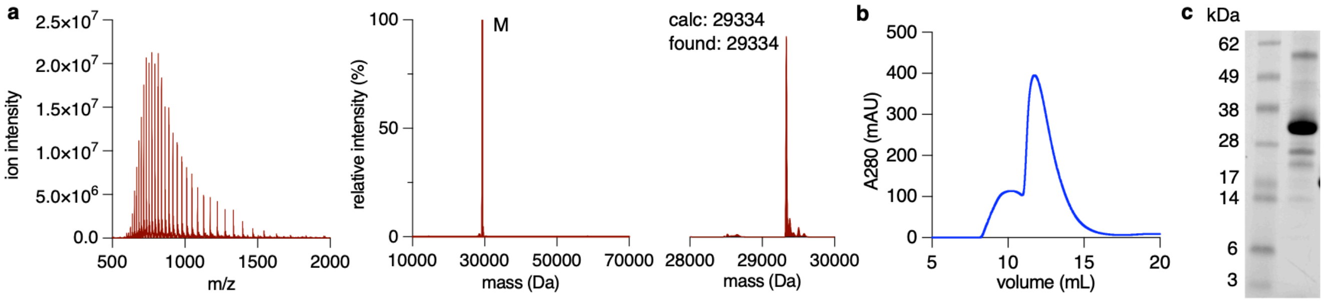

Supporting Figure S30. a, Combined ion series and deconvoluted mass spectra of **sfGFP S147C**. **b**, Chromatogram of SEC purification of **sfGFP S147C**. **c**, SDS-PAGE gel of purified **sfGFP S147C** stained with Coomassie Blue.

Human CD40 DARPin WT (WT_{CD40})

Sequence:	MRGSHHHHHHGSDLGKKLLQAARAGQLDEVRELLKAGADVNAKDTWGFTPLHIAAESGHLEIV<br>EVLLKAGADVNAKDVQGRTPLHIAAHSGHLEIVEVLLKAGADVNAKDFRGWTPLHLAAWSGHL<br>EIVEILLKAGADVNAQDKSGKTPADLAARAGHQDIAEVLQKAA
MW (Da):	17972 (169 AA, with His_6-tag)
Yield:	50 mg/L after SEC purification

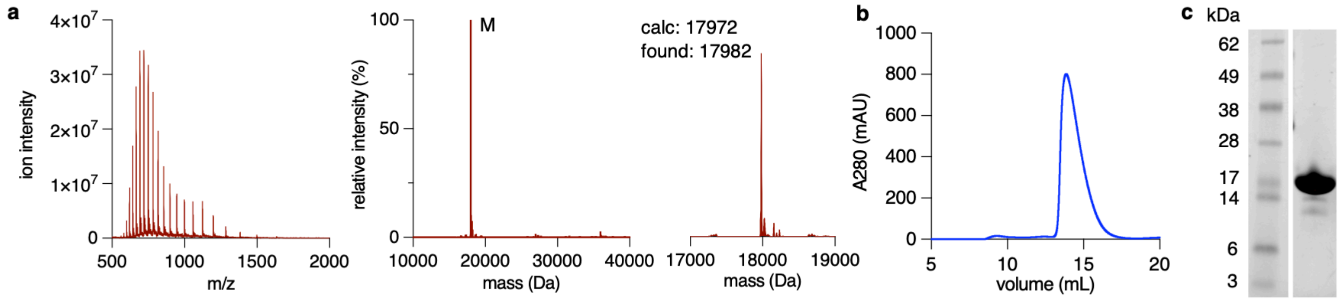

Supporting Figure S31. a, Combined ion series and deconvoluted mass spectra of $\mathbf{WT_{CD40}}$. **b**, Chromatogram of SEC purification of $\mathbf{WT_{CD40}}$. **c**, SDS-PAGE gel of purified $\mathbf{WT_{CD40}}$ stained with Coomassie Blue.

Human CD40 DARPin K4C A144C double mutant (K4C A144C)

Sequence:	MRGSHHHHHHGSDLGCKLLQAARAGQLDEVRELLKAGADVNAKDTWGFTPLHIAAESGHLEIV EVLLKAGADVNAKDVQGRTPLHIAAHSGHLEIVEVLLKAGADVNAKDFRGWTPLHLAAWSGHL EIVEILLKAGADVNAQDKSGKTPADLAARCGHQDIAEVLQKAA
MW (Da):	17979 (169 AA, with His_6-tag)
Yield:	25 mg/L after SEC purification

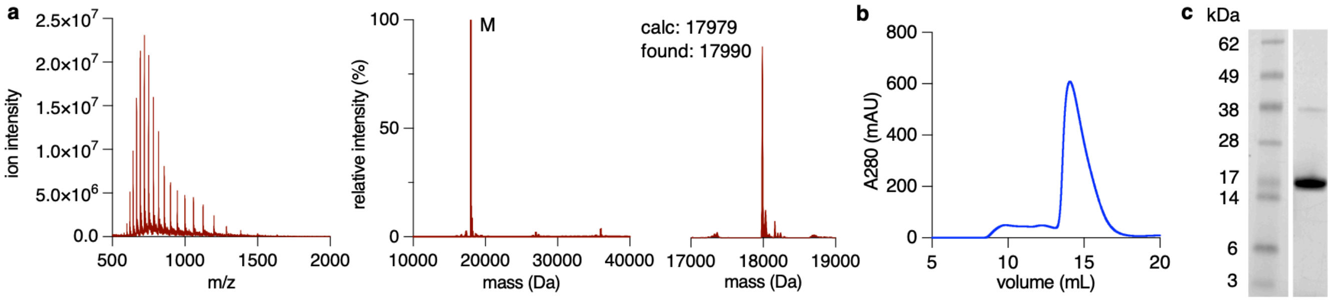

Supporting Figure S32. a, Combined ion series and deconvoluted mass spectra of **K4C A144C**. **b**, Chromatogram of SEC purification of **K4C A144C**. **c**, SDS-PAGE gel of purified **K4C A144C** stained with Coomassie Blue.

Human CD40 DARPin Q8C K135C double mutant (Q8C K135C)

Sequence:	MRGSHHHHHHGSDLGKKLLCAARAGQLDEVRELLKAGADVNAKDTWGFTPLHIAAESGHLEIV EVLLKAGADVNAKDVQGRTPLHIAAHSGHLEIVEVLLKAGADVNAKDFRGWTPLHLAAWSGHL EIVEILLKAGADVNAQDKSGCTPADLAARAGHQDIAEVLQKAA
MW (Da):	17922 (169 AA, with His_6-tag)
Yield:	34 mg/L after SEC purification

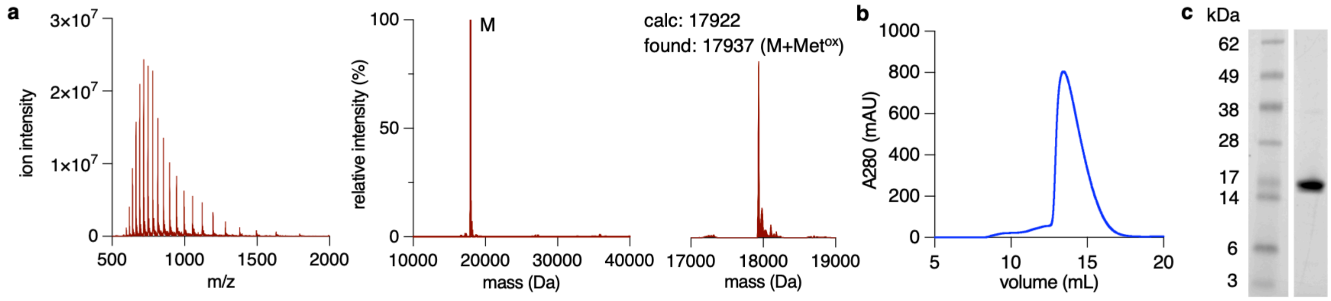

Supporting Figure S33. a, Combined ion series and deconvoluted mass spectra of **Q8C K135C**. **b**, Chromatogram of SEC purification of **Q8C K135C**. **c**, SDS-PAGE gel of purified **Q8C K135C** stained with Coomassie Blue.

Human CD40 DARPin R11C K135C double mutant (R11C K135C)

Sequence:	MRGSHHHHHHGSDLGKKLLQAACAGQLDEVRELLKAGADVNAKDTWGFTPLHIAAESGHLEIV EVLLKAGADVNAKDVQGRTPLHIAAHSGHLEIVEVLLKAGADVNAKDFRGWTPLHLAAWSGHL EIVEILLKAGADVNAQDKSGCTPADLAARAGHQDIAEVLQKAA
MW (Da):	17894 (169 AA, with His$_6$-tag)
Yield:	39 mg/L after SEC purification

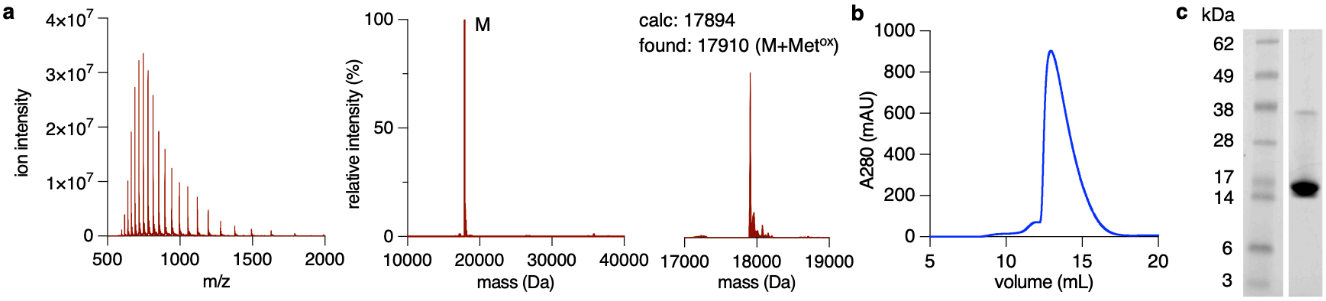

Supporting Figure S34. a, Combined ion series and deconvoluted mass spectra of **R11C K135C**. **b**, Chromatogram of SEC purification of **R11C K135C**. **c**, SDS-PAGE gel of purified **R11C K135C** stained with Coomassie Blue.

Human CD40 DARPin A12C S133C double mutant (A12C S133C)

Sequence:	MRGSHHHHHHGSDLGKKLLQAARCGQLDEVRELLKAGADVNAKDTWGFTPLHIAAESGHLEIV EVLLKAGADVNAKDVQGRTPLHIAAHSGHLEIVEVLLKAGADVNAKDFRGWTPLHLAAWSGHL EIVEILLKAGADVNAQDKCGKTPADLAARAGHQDIAEVLQKAA
MW (Da):	18020 (169 AA, with His$_6$-tag)
Yield:	23 mg/L after SEC purification

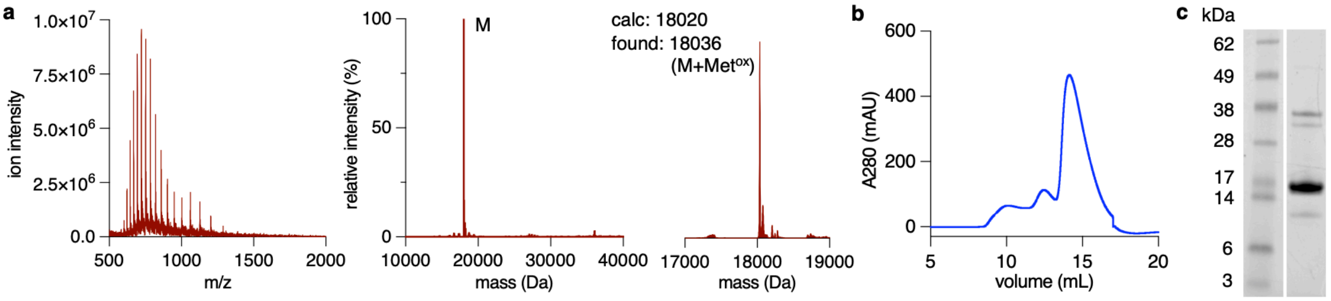

Supporting Figure S35. a, Combined ion series and deconvoluted mass spectra of **A12C S133C**. **b**, Chromatogram of SEC purification of **A12C S133C**. **c**, SDS-PAGE gel of purified **A12C S133C** stained with Coomassie Blue.

Chemical Modifications of Proteins

General Bioconjugation Procedures

General procedure for cysteine modification with maleimides

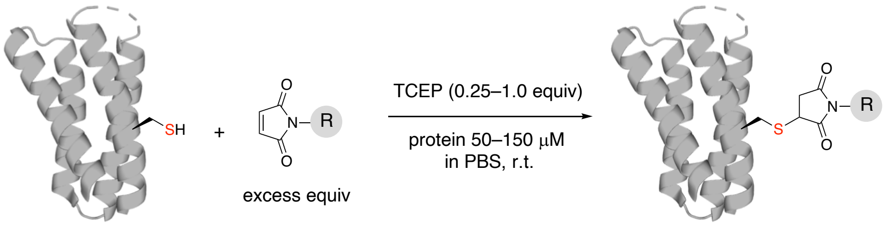

A freshly thawed SEC-purified protein solution (50–150 µM, 1.00 equiv) was placed on ice and a solution of TCEP HCl in water (1.00 mM) was added (0.25–1.00 equiv). From a maleimide stock solution in water (for PNAs, conc: 0.70–2.00 mM) or DMF (for small molecules; conc: 10 mM) was added a volume equivalent to 2.00–3.00 equiv (for PNAs) or 2.00–10.0 equiv (for small molecules) of reactant. The conjugation reaction was mixed using a pipette and allowed to proceed at room temperature without shaking. After 0.5–2.0 h, the reaction was analyzed by LC-MS to confirm full conversion of the protein. In case the reaction was not completed, additional equivalents of maleimide (1.00 equiv for PNAs, 3.00 equiv for small molecules) were added. After full conversion, the reaction mixture was centrifuged at 9000 g for 90 s to remove precipitates formed during the reaction and the conjugate was purified by SEC chromatography. Purified protein conjugates were frozen in liquid nitrogen and stored at -80 °C.

General procedure for homo-dimerization of single cysteine proteins with bis-maleimide PNAs

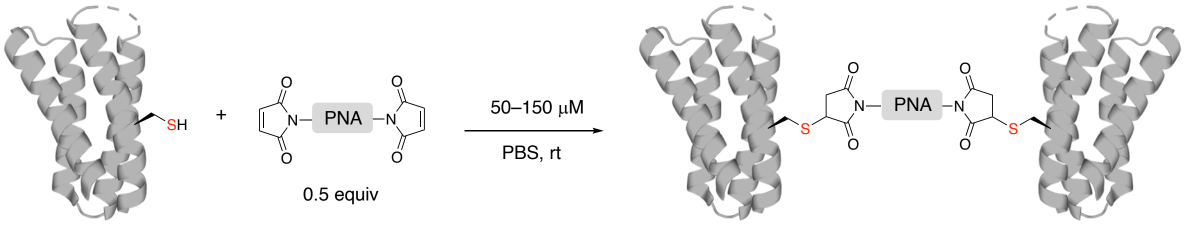

A freshly thawed SEC-purified protein solution in PBS (50–150 µM, 1.00 equiv) was added to a solution of bis-maleimide PNA in PBS–DMF (4:1, v/v) (50–150 µM, 0.50 equiv). The conjugation reaction was mixed using a pipette and allowed to proceed at room temperature without shaking. After 15–30 min, the reaction was analyzed by LC-MS to confirm full conversion of the protein and the reaction mixture was purified by SEC chromatography. Purified protein conjugates were frozen in liquid nitrogen and stored at -80 °C.

General procedure for stapling double cysteine proteins with bis-maleimide PNAs

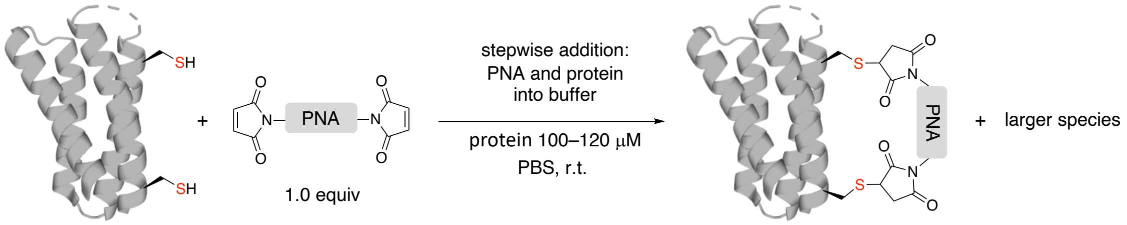

A freshly thawed SEC-purified protein solution in PBS (300 μL, 90–150 μM, 1.00 equiv) and a solution of bis-maleimide PNA in PBS–DMF (4:1, v/v) (300 μL, 90–150 μM, 1.00 equiv) were added simultaneously in 50 μL increments to 400 μL PBS, mixed with a pipette and allowed to react for 2 min before the next addition. After the final addition the reaction was allowed to proceed at room temperature without shaking. After 15 min, the reaction was analyzed by LC-MS to confirm full conversion of the protein and the reaction mixture was concentrated by spin column (3 kDa MWCO) to 0.2–0.3 mL and purified by SEC chromatography. Purified protein conjugates were frozen in liquid nitrogen and stored at -80 °C.

Neo-2/15 Linker Conjugates

Neo-2/15 H8C–L2 (C6)

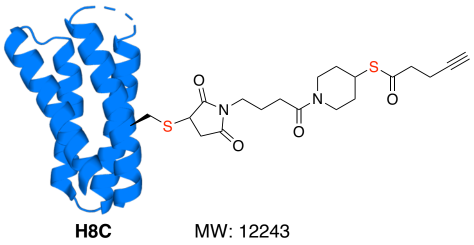

Conjugate **C6** was prepared following the general procedure for cysteine modification with maleimides using protein **H8C** (250 μL, 87 μM, 1.00 equiv), TCEP HCl (21.8 μL, 1.00 mM, 1.00 equiv) and maleimide **L2** in DMF (21.8 μL, 10.0 mM, 10.0 equiv). SEC chromatography afforded the conjugate **C6** (0.5 mL, 8.6 μM, 20%).

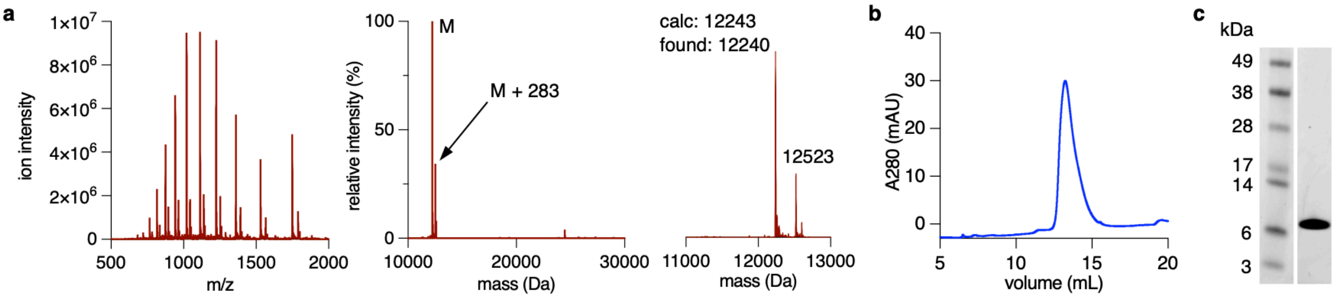

Supporting Figure S36. a, Combined ion series and deconvoluted mass spectra of **C6**. The observed mass of M + 283 Da arises from the hydrolysis of **L2** and the corresponding thiol with another maleimide linker [**L2** (362.4 Da) – pentynoic acid (98.1 Da) + water (18 Da) = 282.3 Da]. **b**, Chromatogram of SEC purification of **C6**. **c**, SDS-PAGE gel of purified **C6** stained with Coomassie Blue.

Neo-2/15 H11C–L1 (C7)

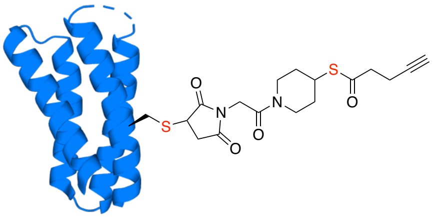

Conjugate **C7** was prepared following the general procedure for cysteine modification with maleimides using protein **H11C** (250 µL, 150 µM, 1.00 equiv), TCEP HCl (37.5 µL, 1.00 mM, 1.00 equiv) and maleimide **L1** in DMF (37.5 µL, 10.0 mM, 10.0 equiv). SEC chromatography afforded the conjugate **C7** (1.0 mL, 14.1 µM, 38%).

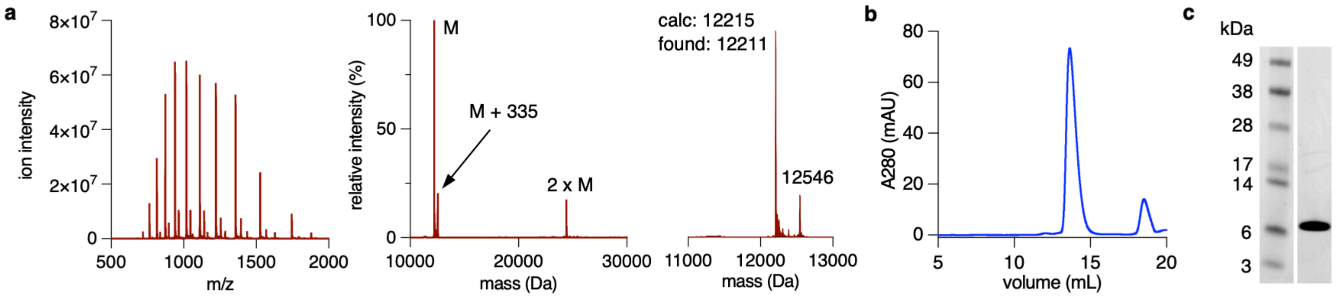

Supporting Figure S37. a, Combined ion series and deconvoluted mass spectra of **C7**. The observed mass of M + 335 Da arises from a second addition of **L1** (334.4 Da). **b**, Chromatogram of SEC purification of **C7**. **c**, SDS-PAGE gel of purified **C7** stained with Coomassie Blue.

Neo-2/15 Y14C–L1 (C8)

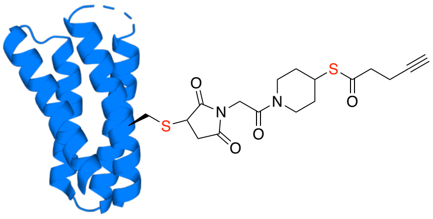

Conjugate **C8** was prepared following the general procedure for cysteine modification with maleimides using protein **Y14C** (500 µL, 53 µM, 1.00 equiv), TCEP HCl (26.5 µL, 1.00 mM, 1.00 equiv) and maleimide **L1** in DMF (15.9 µL, 10.0 mM, 6.00 equiv). SEC chromatography afforded the conjugate **C8** (1.0 mL, 6.1 µM, 23%).

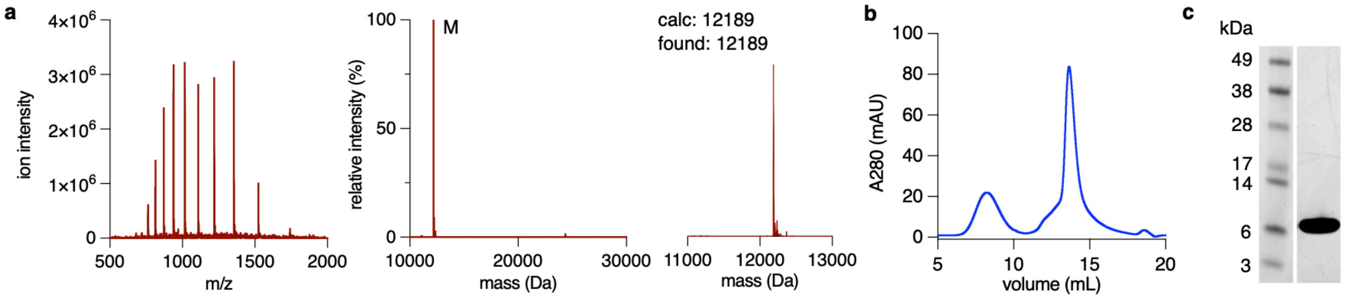

Supporting Figure S38. **a**, Combined ion series and deconvoluted mass spectra of **C8**. **b**, Chromatogram of SEC purification of **C8**. **c**, SDS-PAGE gel of purified **C8** stained with Coomassie Blue.

Neo-2/15 D15C–L1 (C9)

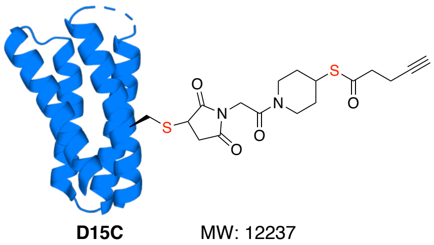

Conjugate **C9** was prepared following the general procedure for cysteine modification with maleimides using protein **D15C** (250 µL, 115 µM, 1.00 equiv), TCEP HCl (28.8 µL, 1.00 mM, 1.00 equiv) and maleimide **L1** in DMF (28.8 µL, 10.0 mM, 10.0 equiv). SEC chromatography afforded the conjugate **C9** (1.5 mL, 5.9 µM, 31%).

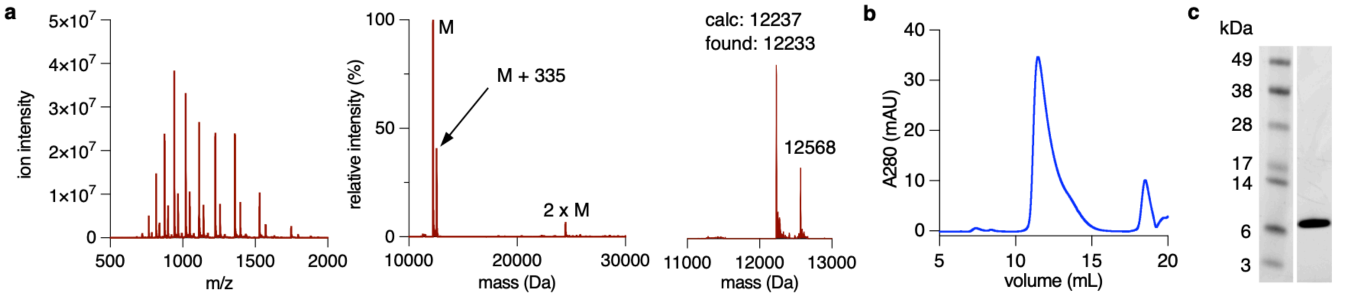

Supporting Figure S39. **a**, Combined ion series and deconvoluted mass spectra of **C9**. The observed mass of M + 335 Da arises from a second addition of **L1** (334.4 Da). **b**, Chromatogram of SEC purification of **C9**. **c**, SDS-PAGE gel of purified **C9** stained with Coomassie Blue.

Neo-2/15 K33C–L2 (C10)

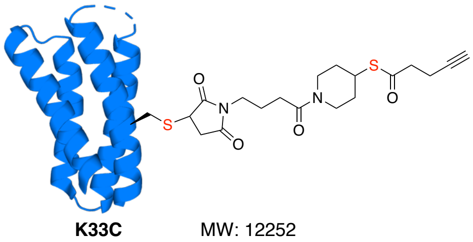

Conjugate **C10** was prepared following the general procedure for cysteine modification with maleimides using protein **K33C** (250 µL, 104 µM, 1.00 equiv), TCEP HCl (26.0 µL, 1.00 mM, 1.00 equiv) and maleimide **L2** in DMF (26.0 µL, 10.0 mM, 10.0 equiv). SEC chromatography afforded the conjugate **C10** (1.2 mL, 6.6 µM, 31%).

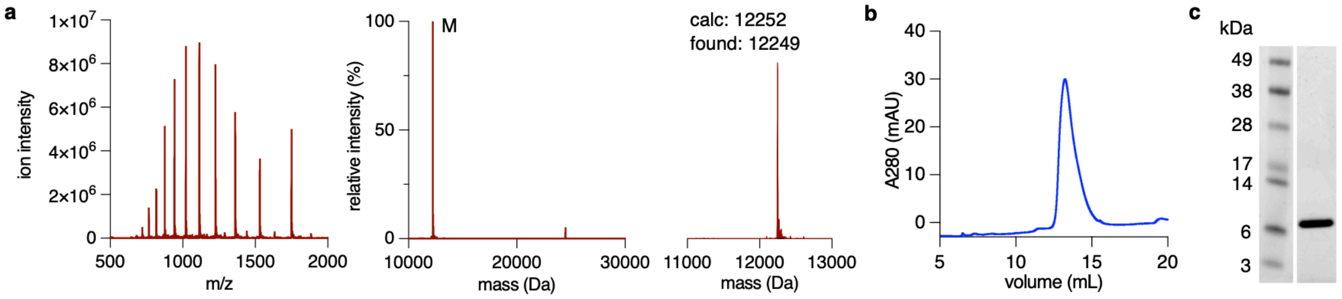

Supporting Figure S40. a, Combined ion series and deconvoluted mass spectra of **C10**. **b**, Chromatogram of SEC purification of **C10**. **c**, SDS-PAGE gel of purified **C10** stained with Coomassie Blue.

Neo-2/15 L7C K33C–L1 (C15)

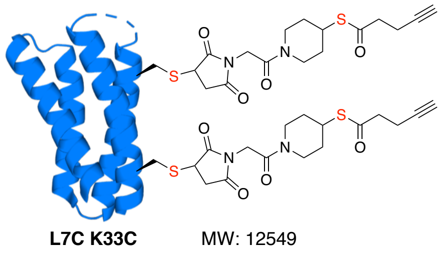

Conjugate **C15** was prepared following the general procedure for cysteine modification with maleimides using protein **L7C K33C** (350 µL, 101 µM, 1.00 equiv), TCEP HCl (35.4 µL, 1.00 mM, 1.00 equiv) and maleimide **L1** in DMF (21.2 µL, 10.0 mM, 6.00 equiv). SEC chromatography afforded the conjugate **C15** (1.5 mL, 2.4 µM, 10%).

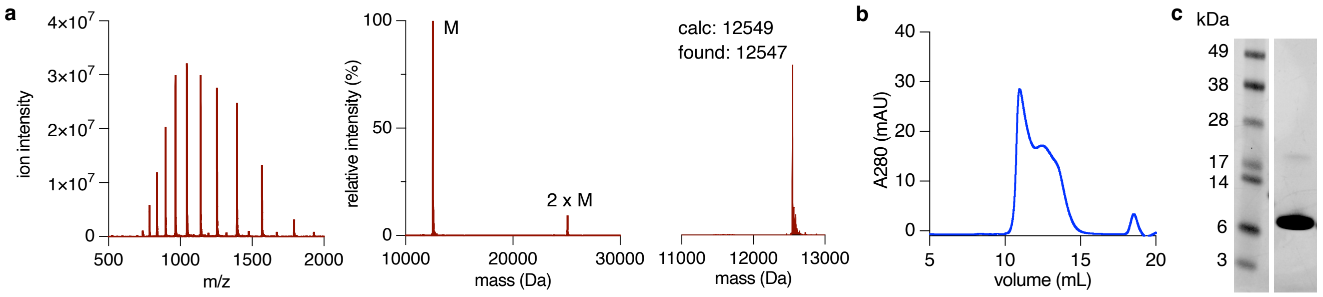

Supporting Figure S41. a, Combined ion series and deconvoluted mass spectra of **C15**. **b**, Chromatogram of SEC purification of **C15**. **c**, SDS-PAGE gel of purified **C15** stained with Coomassie Blue.

Neo-2/15 Y14C K33C–L2 (C16)

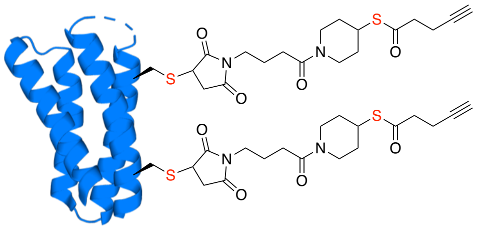

Conjugate **C16** was prepared following the general procedure for cysteine modification with maleimides using protein **Y14C K33C** (250 µL, 118 µM, 1.00 equiv), TCEP HCl (29.3 µL, 1.00 mM, 1.00 equiv) and maleimide **L2** in DMF (29.3 µL, 10.0 mM, 10.0 equiv). SEC chromatography afforded the conjugate **C16** (1.0 mL, 8.4 µM, 28%).

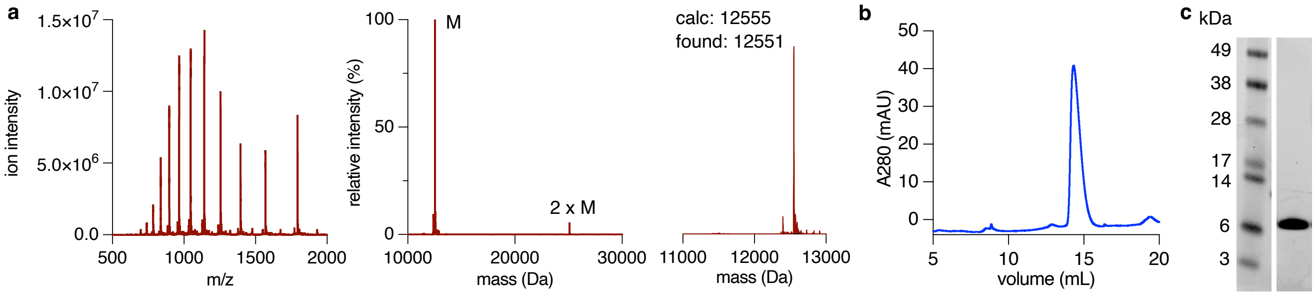

Supporting Figure S42. a, Combined ion series and deconvoluted mass spectra of **C16**. **b**, Chromatogram of SEC purification of **C16**. **c**, SDS-PAGE gel of purified **C16** stained with Coomassie Blue.

Neo-2/15 K33C E47C–L1 (C17)

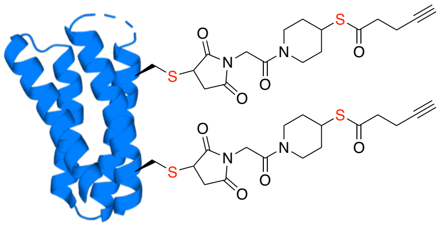

Conjugate **C17** was prepared following the general procedure for cysteine modification with maleimides using protein **K33C E47C** (350 µL, 114 µM, 1.00 equiv), TCEP HCl (39.9 µL, 1.00 mM, 1.00 equiv) and maleimide **L1** in DMF (23.9 µL, 10.0 mM, 6.00 equiv). SEC chromatography afforded the conjugate **C17** (1.0 mL, 19.0 µM, 48%).

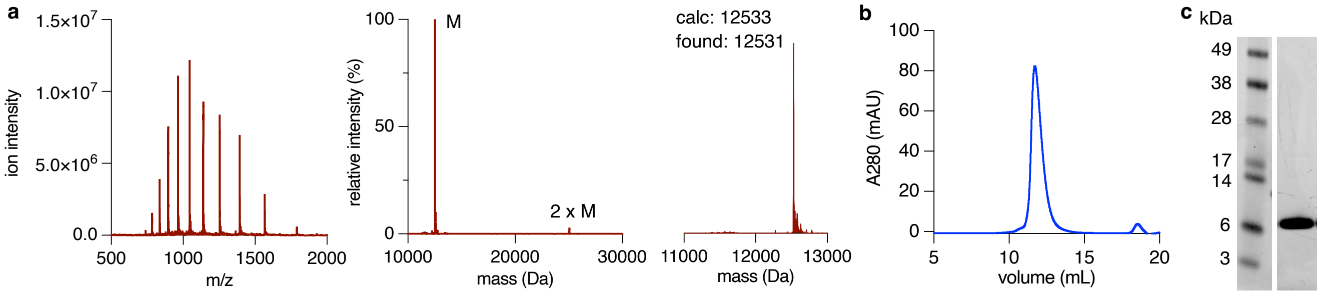

Supporting Figure S43. **a**, Combined ion series and deconvoluted mass spectra of **C17**. **b**, Chromatogram of SEC purification of **C17**. **c**, SDS-PAGE gel of purified **C17** stained with Coomassie Blue.

Neo-2/15 K33C L51C–L1 (C18)

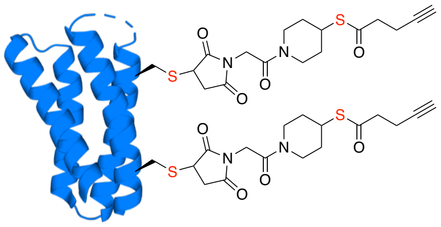

Conjugate **C18** was prepared following the general procedure for cysteine modification with maleimides using protein **K33C L51C** (350 μL, 119 μM, 1.00 equiv), TCEP HCl (41.7 μL, 1.00 mM, 1.00 equiv) and maleimide **L1** in DMF (25.0 μL, 10.0 mM, 6.00 equiv). SEC chromatography afforded the conjugate **C18** (1.5 mL, 8.9 μM, 32%).

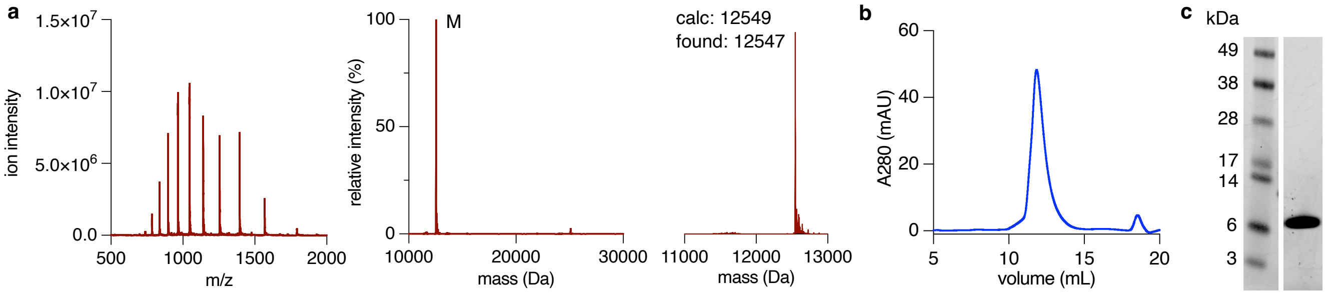

Supporting Figure S44. **a**, Combined ion series and deconvoluted mass spectra of **C18**. **b**, Chromatogram of SEC purification of **C18**. **c**, SDS-PAGE gel of purified **C18** stained with Coomassie Blue.

Neo-2/15 PNA Conjugates

Neo-2/15 H8C–P2 (C1)

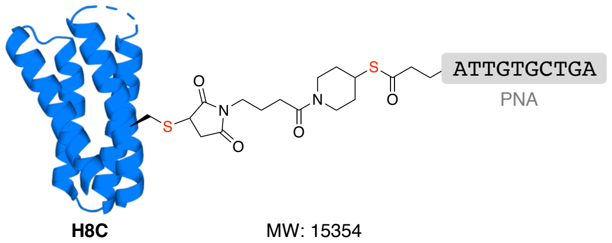

Conjugate **C1** was prepared following the general procedure for cysteine modification with maleimides using protein **H8C** (250 µL, 83 µM, 1.00 equiv), TCEP HCl (6.50 µL, 1.00 mM, 0.30 equiv) and maleimide–PNA **P2** in water (60.8 µL, 0.72 mM, 2.00 equiv). After 2 h, additional 2.00 equiv of maleimide–PNA **P2** were added to reach full conversion. SEC chromatography afforded the conjugate **C1** (1.0 mL, 3.4 µM, 16%).

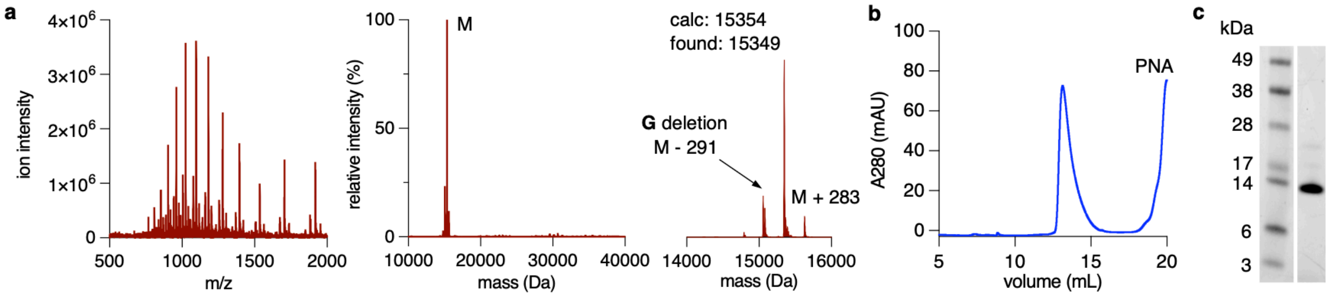

Supporting Figure S45. a, Combined ion series and deconvoluted mass spectra of **C1**. The observed mass of M + 283 Da arises from the hydrolysis of **L2** and the corresponding thiol with another maleimide linker [**L2** (362.4 Da) – pentynoic acid (98.1 Da) + water (18 Da) = 282.3 Da]. **b**, Chromatogram of SEC purification of **C1**. **c**, SDS-PAGE gel of purified **C1** stained with Coomassie Blue.

Neo-2/15 H11C–P1 (C2)

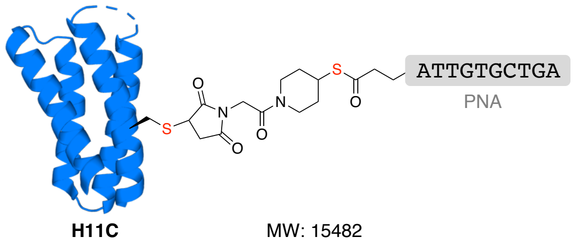

Conjugate **C2** was prepared following the general procedure for cysteine modification with maleimides using protein **H11C** (400 µL, 94 µM, 1.00 equiv), TCEP HCl (19.0 µL, 1.00 mM, 0.50 equiv) and maleimide–PNA **P1** in water (36.2 µL, 2.08 mM, 2.00 equiv). SEC chromatography afforded the conjugate **C2** (2.0 mL, 2.9 µM, 15%).

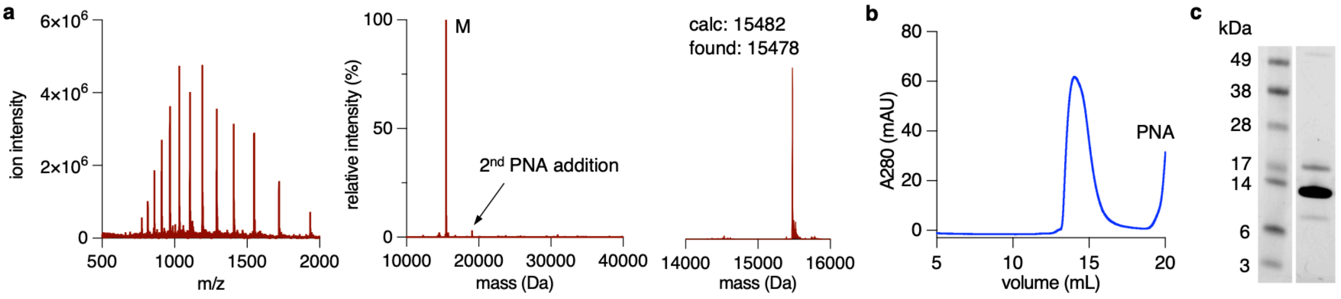

Supporting Figure S46. a, Combined ion series and deconvoluted mass spectra of **C2**. **b**, Chromatogram of SEC purification of **C2**. **c**, SDS-PAGE gel of purified **C2** stained with Coomassie Blue.

Neo-2/15 Y14C–P2 (C3)

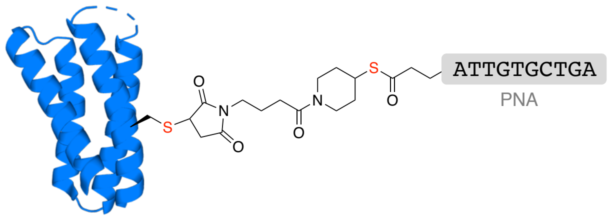

Y14C MW: 15328

Conjugate **C3** was prepared following the general procedure for cysteine modification with maleimides using protein **Y14C** (250 µL, 53 µM, 1.00 equiv), TCEP HCl (13.0 µL, 1.00 mM, 1.00 equiv) and maleimide–PNA **P2** in water (55.5 µL, 0.72 mM, 3.00 equiv). SEC chromatography afforded the conjugate **C3** (1.5 mL, 3.3 µM, 37%).

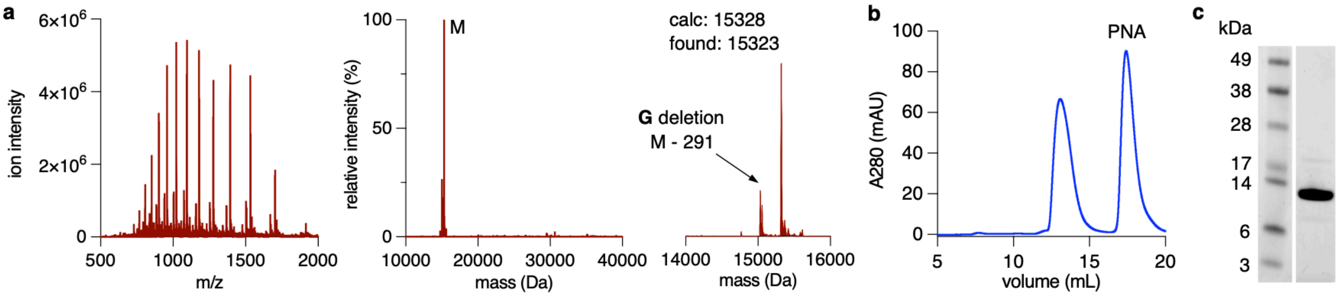

Supporting Figure S47. a, Combined ion series and deconvoluted mass spectra of **C3**. **b**, Chromatogram of SEC purification of **C3**. **c**, SDS-PAGE gel of purified **C3** stained with Coomassie Blue.

Neo-2/15 K33C–P2 (C4)

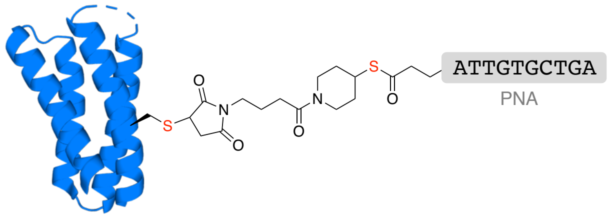

K33C MW: 15363

Conjugate **C4** was prepared following the general procedure for cysteine modification with maleimide using protein **K33C** (250 µL, 104 µM, 1.00 equiv), TCEP HCl (7.80 µL, 1.00 mM, 0.30 equiv) and maleimide–PNA **P2** in water (72.6 µL, 0.72 mM, 2.00 equiv). SEC chromatography afforded the conjugate **C4** (1.0 mL, 9.9 µM, 38%).

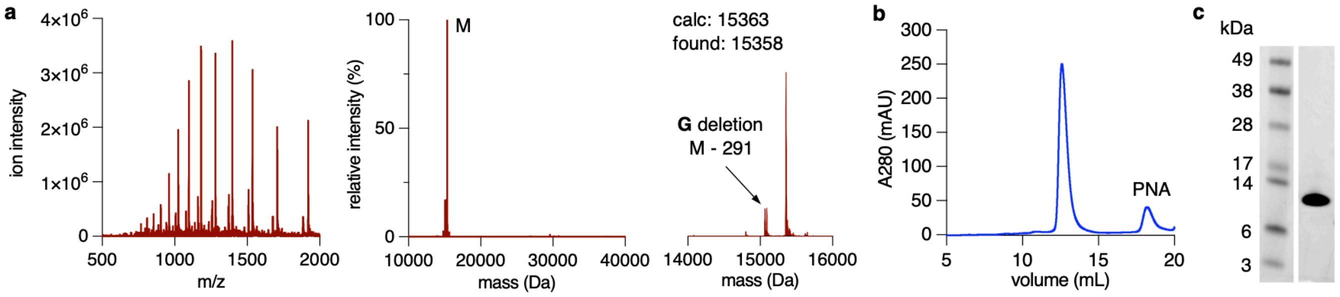

Supporting Figure S48. a, Combined ion series and deconvoluted mass spectra of **C4**. **b**, Chromatogram of SEC purification of **C4**. **c**, SDS-PAGE gel of purified **C4** stained with Coomassie Blue.

Neo-2/15 L7C K33C–P4 staple (C11)

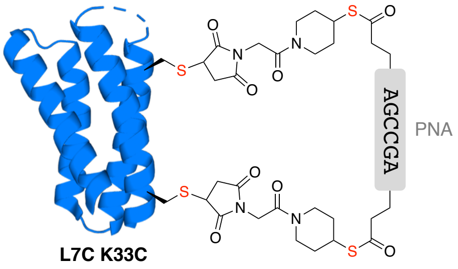

MW: 14708

Conjugate **C11** was prepared following the general procedure for stapling of double cysteine proteins with bis-maleimide PNAs by combining protein **L7C K33C** (200 µL, 101 µM, 1.00 equiv) and bis-maleimide–PNA **P4** in PBS–DMF (4:1, v/v) (200 µL, 101 µM, 1.00 equiv) batch-wise in PBS (100 µL). SEC chromatography afforded the conjugate **C11** (1.0 mL, 2.3 µM, 11%).

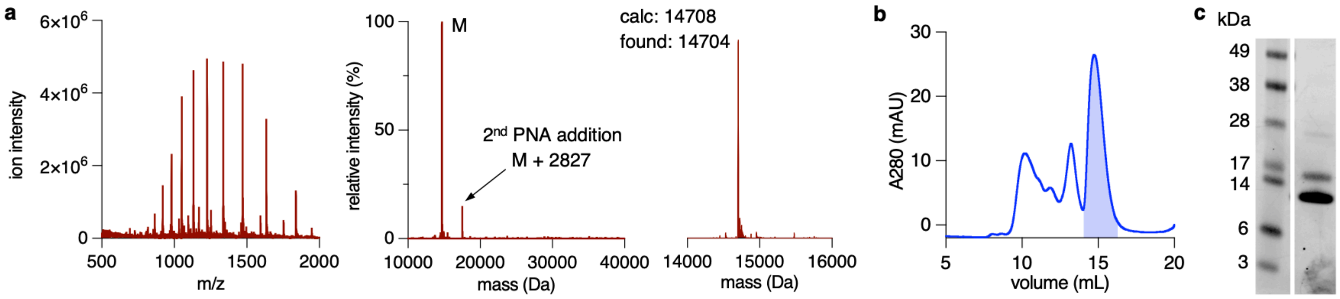

Supporting Figure S49. a, Combined ion series and deconvoluted mass spectra of **C11**. **b**, Chromatogram of SEC purification of **C11**. **c**, SDS-PAGE gel of purified **C11** stained with Coomassie Blue.

Neo-2/15 Y14C K33C–P5 staple (C12)

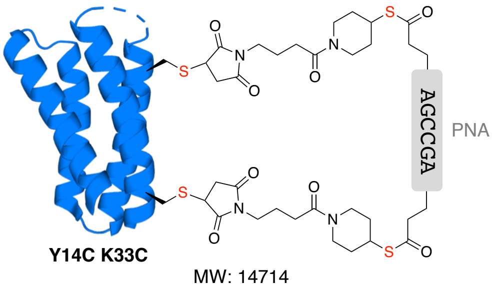

Conjugate **C12** was prepared following the general procedure for stapling of double cysteine proteins with bis-maleimide PNAs by combining protein **Y14C K33C** (200 µL, 117 µM, 1.00 equiv) and bis-maleimide–PNA **P5** in PBS–DMF (4:1, v/v) (200 µL, 117 µM, 1.00 equiv) batch-wise in PBS (100 µL). SEC chromatography afforded the conjugate **C12** (1.5 mL, 2.5 µM, 16%).

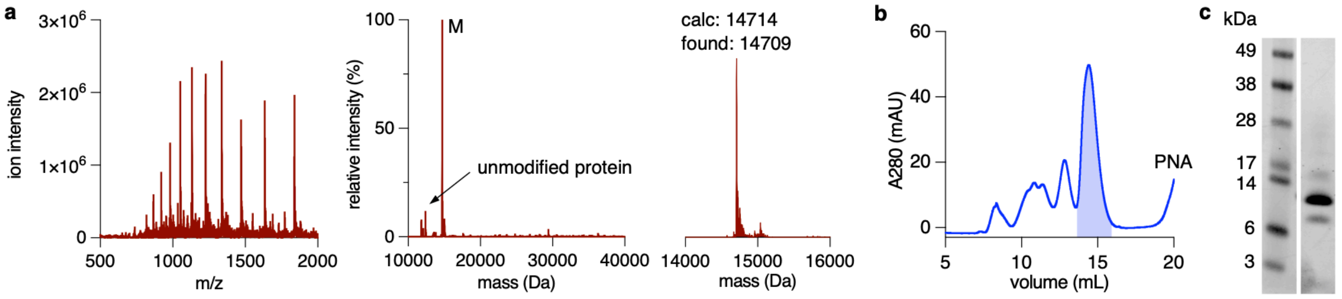

Supporting Figure S50. **a**, Combined ion series and deconvoluted mass spectra of **C12**. **b**, Chromatogram of SEC purification of **C12**. **c**, SDS-PAGE gel of purified **C12** stained with Coomassie Blue.

Neo-2/15 K33C E47C–P4 staple (C13)

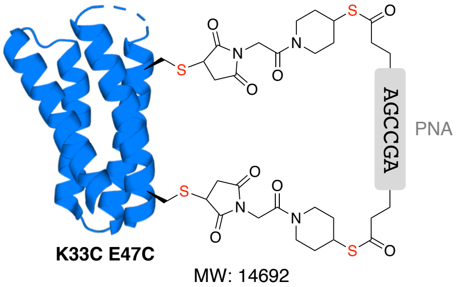

Conjugate **C13** was prepared following the general procedure for stapling of double cysteine proteins with bis-maleimide PNAs by combining protein **K33C E47C** (200 µL, 114 µM, 1.00 equiv) and bis-maleimide–PNA **P4** in PBS–DMF (4:1, v/v) (200 µL, 114 µM, 1.00 equiv) batch wise in PBS (100 µL). SEC chromatography afforded the conjugate **C13** (1.0 mL, 1.5 µM, 11%).

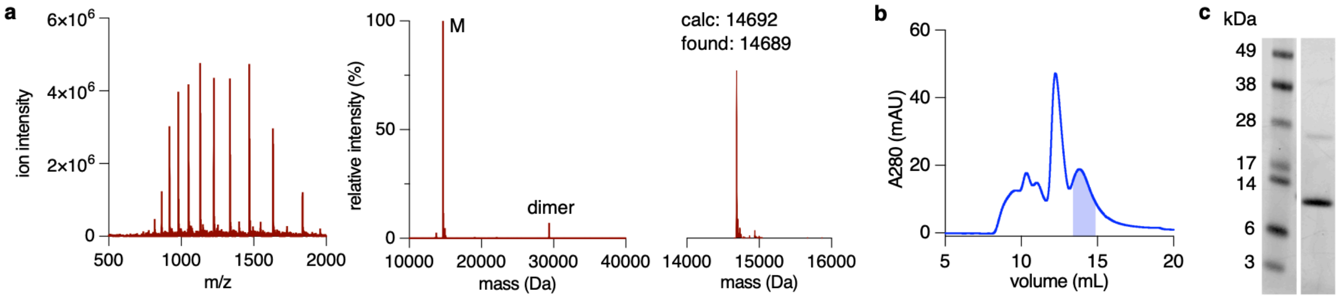

Supporting Figure S51. a, Combined ion series and deconvoluted mass spectra of **C13**. **b**, Chromatogram of SEC purification of **C13**. **c**, SDS-PAGE gel of purified **C13** stained with Coomassie Blue.

Neo-2/15 K33C L51C–P4 staple (C14)

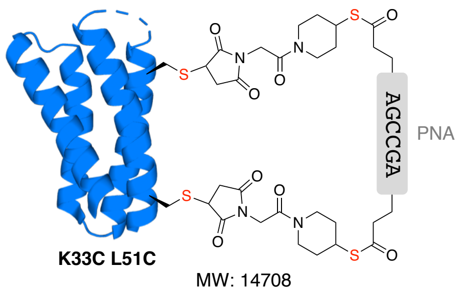

Conjugate **C14** was prepared following the general procedure for stapling of double cysteine proteins with bis-maleimide PNAs by combining protein **K33C L51C** (200 µL, 119 µM, 1.00 equiv) and bis-maleimide–PNA **P4** in PBS–DMF (4:1, v/v) (200 µL, 119 µM, 1.00 equiv) batch wise in PBS (100 µL). SEC chromatography afforded the conjugate **C14** (0.7 mL, 0.7 µM, 2%).

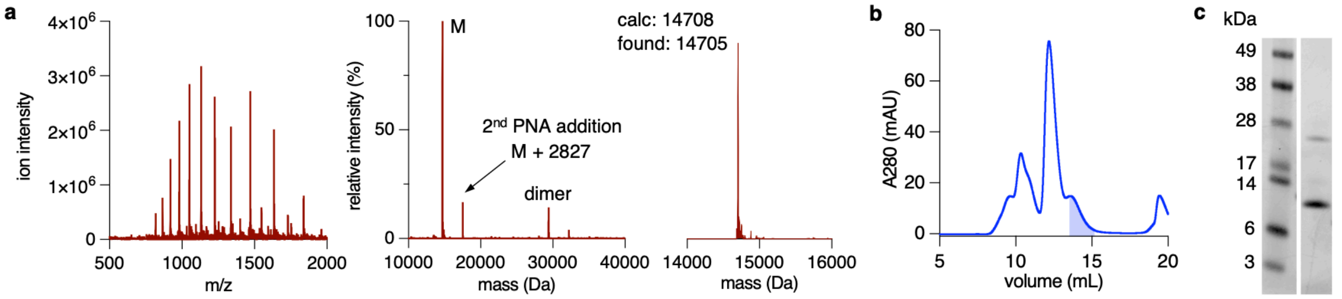

Supporting Figure S52. a, Combined ion series and deconvoluted mass spectra of **C14**. **b**, Chromatogram of SEC purification of **C14**. **c**, SDS-PAGE gel of purified **C14** stained with Coomassie Blue.

Neo-2/15 H8C–P4 dimer (SC3)

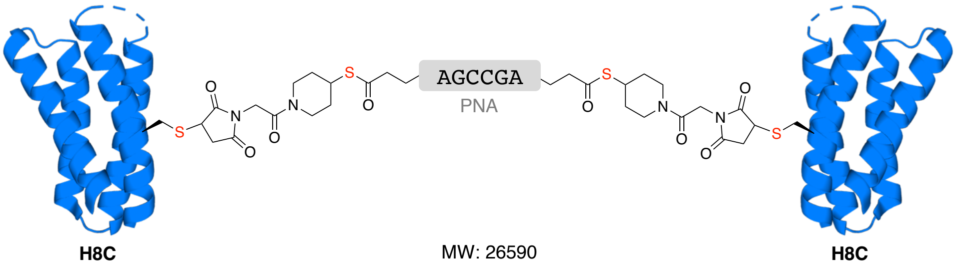

Conjugate **SC3** was prepared following the general procedure for homo-dimerization of single cysteine proteins with bis-maleimide PNAs using protein **H8C** (250 µL, 87 µM, 1.00 equiv) and bis-maleimide–PNA **P4** in water PBS–DMF (4:1, v/v) (125 µL, 87 µM, 0.50 equiv). SEC chromatography afforded the conjugate **SC3** (1.0 mL, 5.5 µM, 25%).

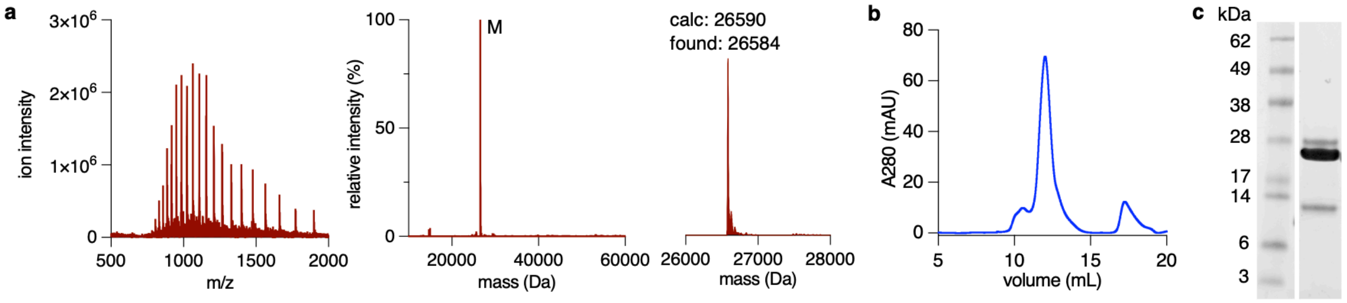

Supporting Figure S53. **a**, Combined ion series and deconvoluted mass spectra of **SC3**. **b**, Chromatogram of SEC purification of **SC3**. **c**, SDS-PAGE gel of purified **SC3** stained with Coomassie Blue.

Neo-2/15 H11C–P4 dimer (SC4)

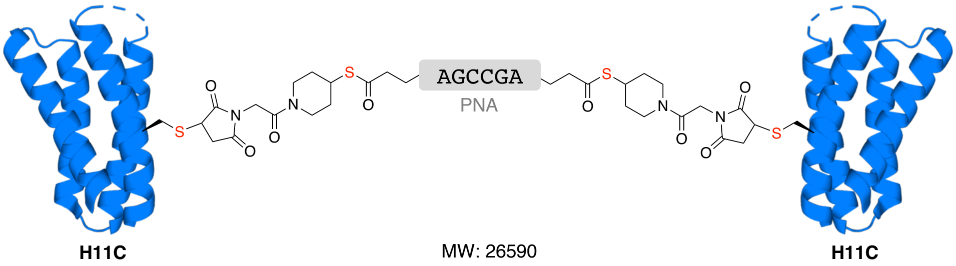

Conjugate **SC4** was prepared following the general procedure for homo-dimerization of single cysteine proteins with bis-maleimide PNAs using protein **H11C** (250 µL, 150 µM, 1.00 equiv) and bis-maleimide–PNA **P4** in water PBS–DMF (8:2, v/v) (125 µL, 150 µM, 0.50 equiv). SEC chromatography afforded the conjugate **SC4** (1.0 mL, 7.8 µM, 22%).

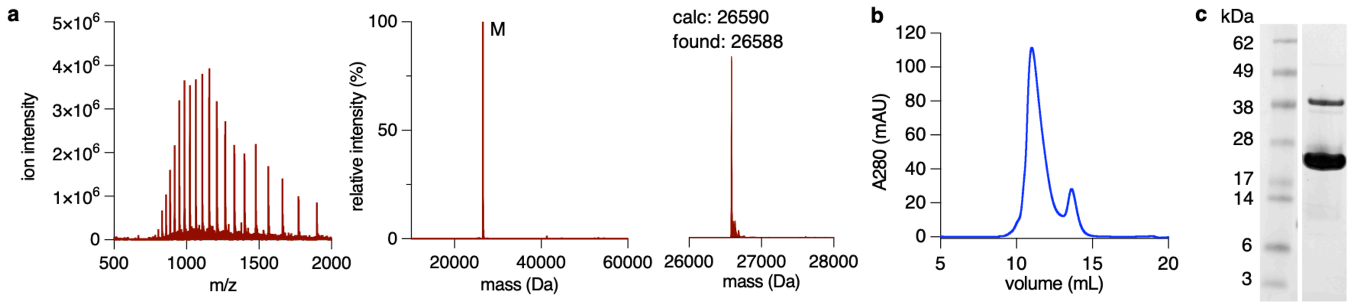

Supporting Figure S54. a, Combined ion series and deconvoluted mass spectra of **SC4**. **b**, Chromatogram of SEC purification of **SC4**. **c**, SDS-PAGE gel of purified **SC4** stained with Coomassie Blue.

Neo-2/15 Y14C–P4 dimer (SC5)

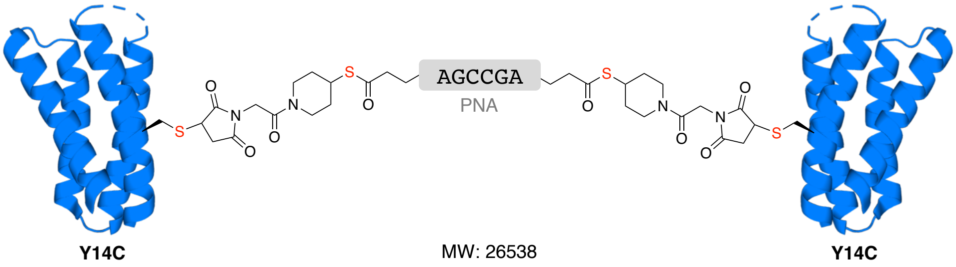

Conjugate **SC5** was prepared following the general procedure for homo-dimerization of single cysteine proteins with bis-maleimide PNAs using protein **Y14C** (250 µL, 109 µM, 1.00 equiv) and bis-maleimide–PNA **P4** in water PBS–DMF (8:2, v/v) (125 µL, 109 µM, 0.50 equiv). SEC chromatography afforded the conjugate **SC5** (1.0 mL, 7.1 µM, 26%).

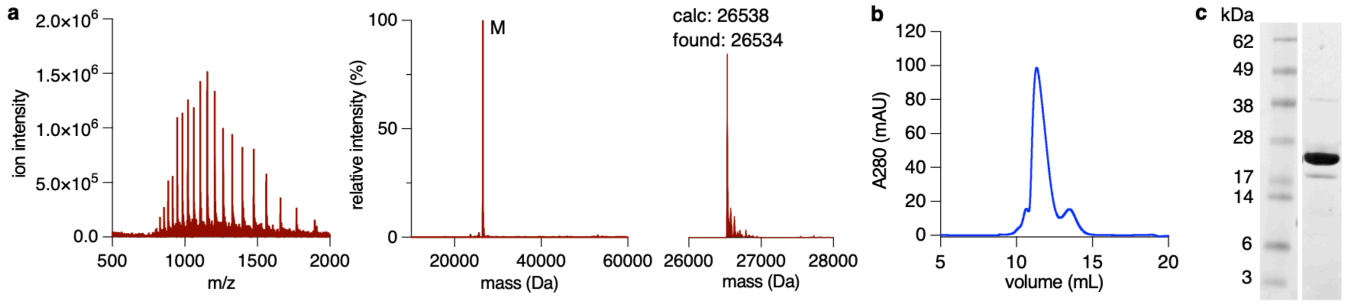

Supporting Figure S55. a, Combined ion series and deconvoluted mass spectra of **SC5**. **b**, Chromatogram of SEC purification of **SC5**. **c**, SDS-PAGE gel of purified **SC5** stained with Coomassie Blue.

Neo-2/15 K33C–P4 dimer (SC6)

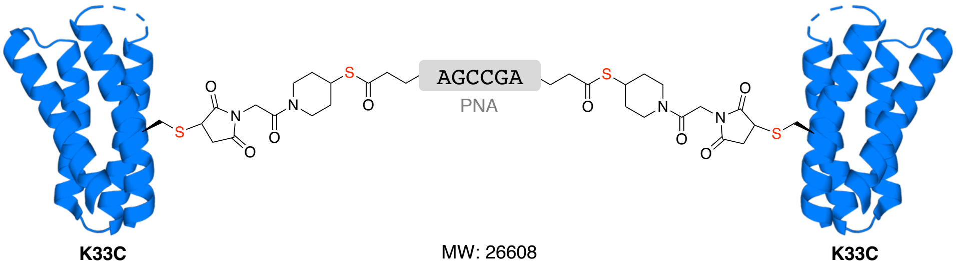

Conjugate **SC6** was prepared following the general procedure for homo-dimerization of single cysteine proteins with bis-maleimide PNAs using protein **K33C** (250 µL, 104 µM, 1.00 equiv) and bis-maleimide–PNA **P4** in water PBS–DMF (8:2, v/v) (125 µL, 104 µM, 0.50 equiv). SEC chromatography afforded the conjugate **SC6** (1.0 mL, 5.3 µM, 20%).

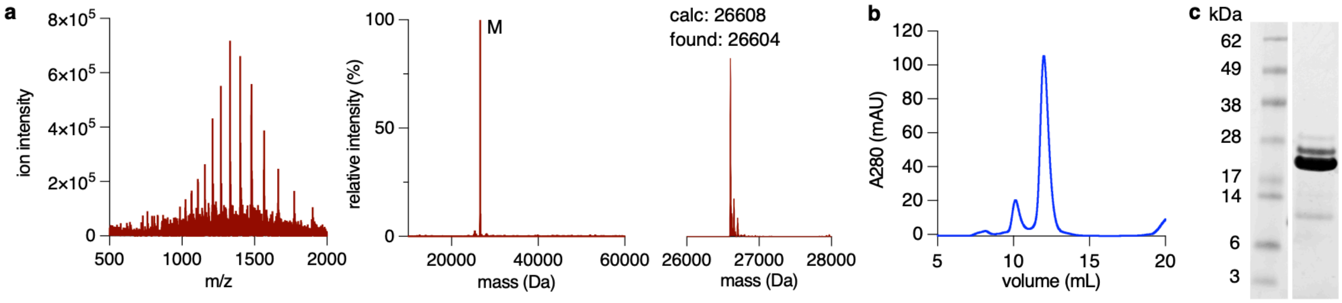

Supporting Figure S56. a, Combined ion series and deconvoluted mass spectra of **SC6**. **b**, Chromatogram of SEC purification of **SC6**. **c**, SDS-PAGE gel of purified **SC6** stained with Coomassie Blue.

Neo-2/15 K33C E47C–P4 dimer (SC7)

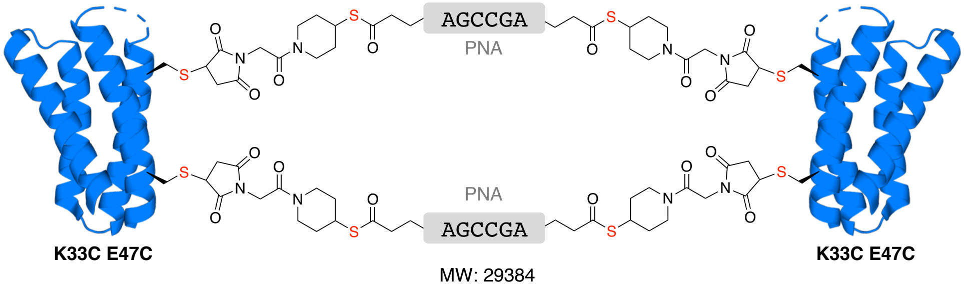

Conjugate **SC7** was isolated from the preparation of **C13**. SEC chromatography afforded the bioconjugate **SC7** (1.0 mL, 3.1 µM).

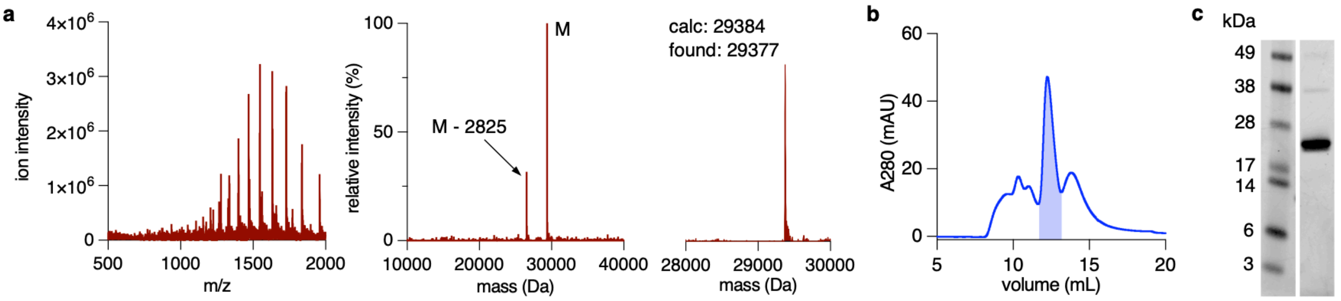

Supporting Figure S57. **a**, Combined ion series and deconvoluted mass spectra of **SC7**. **b**, Chromatogram of SEC purification of **SC7**. **c**, SDS-PAGE gel of purified **SC7** stained with Coomassie Blue.

Neo-2/15 K33C L51C–P4 dimer (C19)

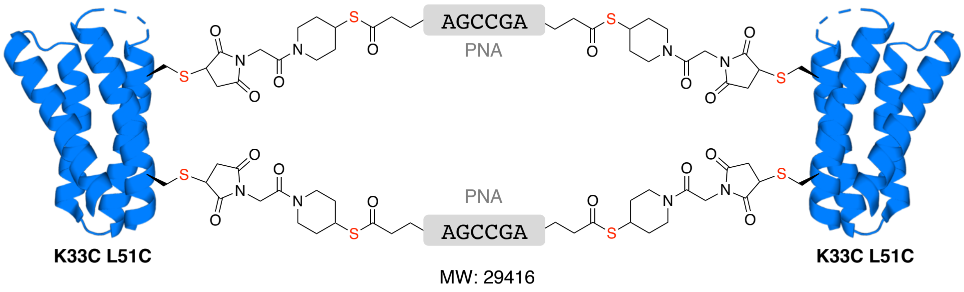

A freshly thawed SEC-purified solution of **L51C K33C** in PBS (4.0 mL, 110 µM, 1.00 equiv) was added dropwise over 5 min to a slowly stirred solution of bis-maleimide PNA **P4** in PBS–DMF (4:1, v/v) (4.8 mL, 110 µM, 1.10 equiv) and the reaction was allowed proceed at room temperature. After 15 min, the reaction was analyzed by LC-MS to confirm full conversion of the protein and the reaction mixture was concentrated using a centrifugal concentrator (3 kDa MWCO) to 1.5 mL and purified by SEC chromatography applying 0.25 mL injection volumes to afford conjugate **C19** (5.0 mL, 16.1 µM, 18%).

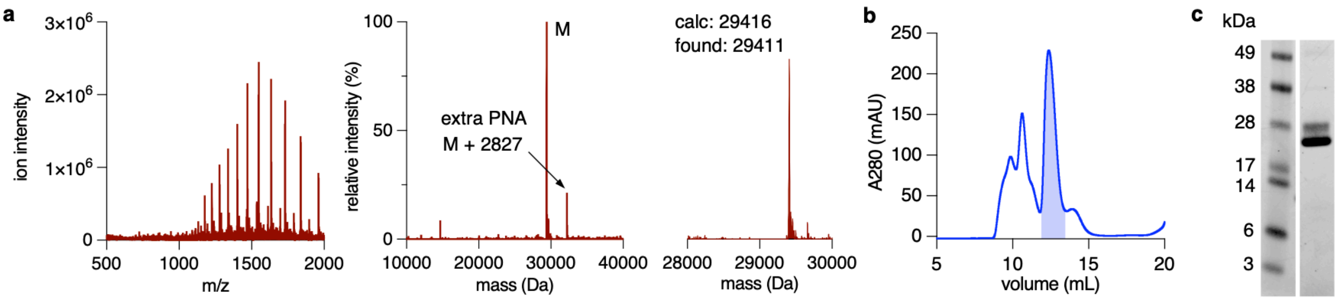

Supporting Figure S58. **a**, Combined ion series and deconvoluted mass spectra of **C19**. **b**, Chromatogram of SEC purification of **C19**. **c**, SDS-PAGE gel of purified **C19** stained with Coomassie Blue.

DARPin Linker Conjugates

Human CD40 DARPin K4C A144C–L1 (C25)

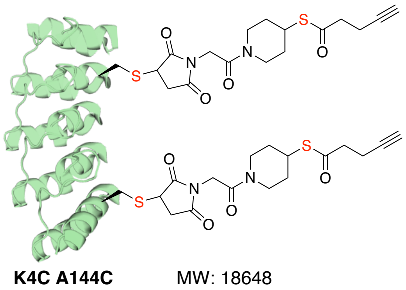

Conjugate **C25** was prepared following the general procedure for cysteine modification with maleimide using protein **K4C A144C** (250 µL, 111 µM, 1.00 equiv), TCEP HCl (27.8 µL, 1.00 mM, 1.00 equiv) and maleimide **L1** in DMF (16.7 µL, 10.0 mM, 6.00 equiv). SEC chromatography afforded the conjugate **C25** (1.0 mL, 8.3 µM, 30%).

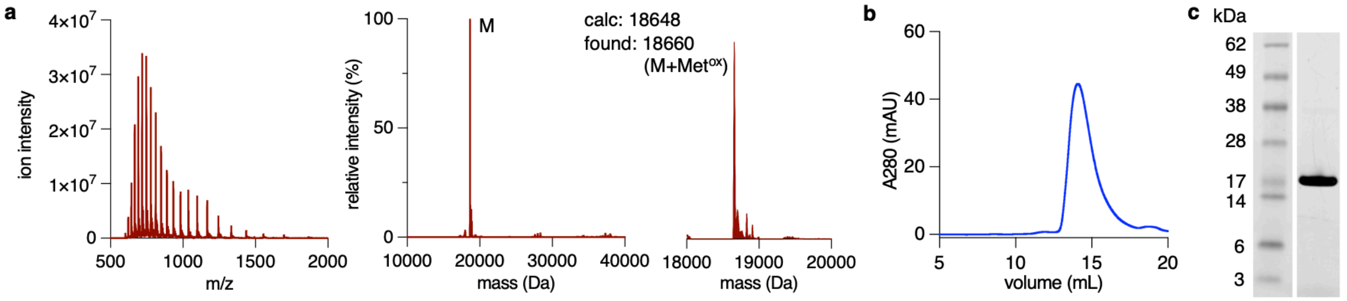

Supporting Figure S59. a, Combined ion series and deconvoluted mass spectra of **C25**. **b**, Chromatogram of SEC purification of **C25**. **c**, SDS-PAGE gel of purified **C25** stained with Coomassie Blue.

Human CD40 DARPin Q8C K135C–L1 (C26)

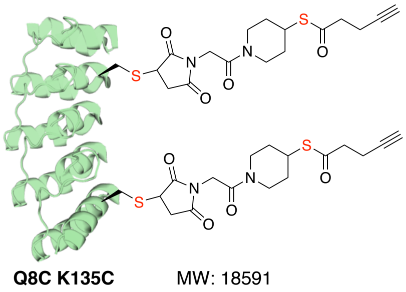

Conjugate **C26** was prepared following the general procedure for cysteine modification with maleimide using protein **Q8C K135C** (300 µL, 139 µM, 1.00 equiv), TCEP HCl (42.0 µL, 1.00 mM, 1.00 equiv) and maleimide **L1** in DMF (25.0 µL, 10.0 mM, 6.00 equiv). SEC chromatography afforded the conjugate **C26** (0.9 mL, 18 µM, 39%).

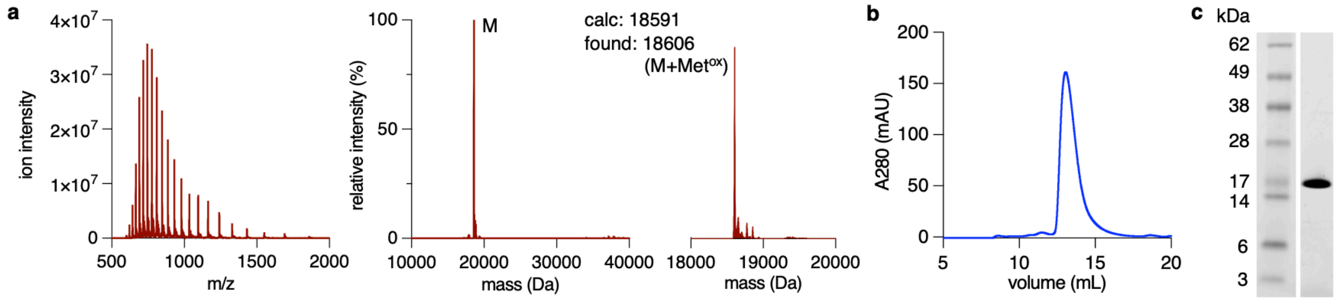

Supporting Figure S60. **a**, Combined ion series and deconvoluted mass spectra of **C26**. **b**, Chromatogram of SEC purification of **C26**. **c**, SDS-PAGE gel of purified **C26** stained with Coomassie Blue.

Human CD40 DARPin R11C K135C–L1 (C27)

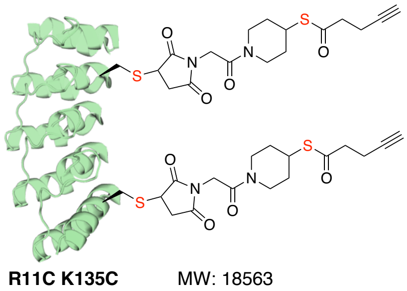

Conjugate **C27** was prepared following the general procedure for cysteine modification with maleimide using protein **R11C K135C** (300 µL, 142 µM, 1.00 equiv), TCEP HCl (43.0 µL, 1.00 mM, 1.00 equiv) and maleimide **L1** in DMF (25.7 µL, 10.0 mM, 6.00 equiv). SEC chromatography afforded the conjugate **C27** (0.9 mL, 15.1 µM, 32%).

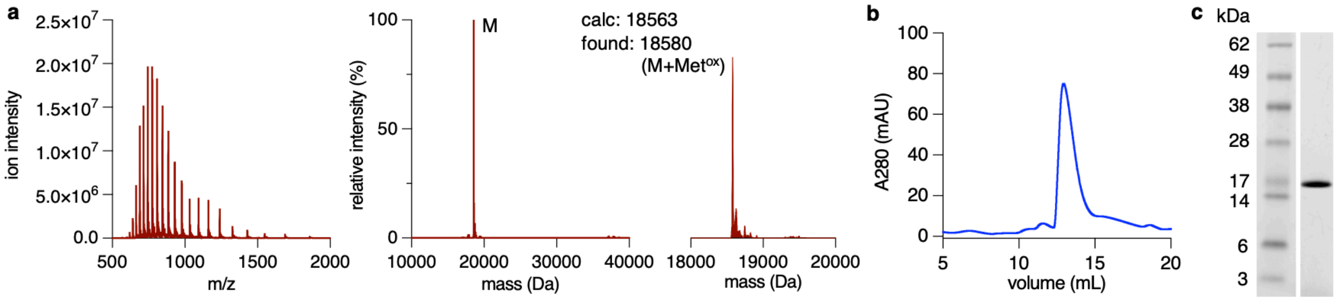

Supporting Figure S61. **a**, Combined ion series and deconvoluted mass spectra of **C27**. **b**, Chromatogram of SEC purification of **C27**. **c**, SDS-PAGE gel of purified **C27** stained with Coomassie Blue.

Human CD40 DARPin A12C S133C–L1 (C28)

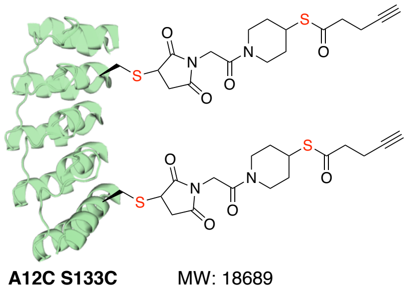

Conjugate **C28** was prepared following the general procedure for cysteine modification with maleimide using protein **A12C S133C** (250 µL, 91 µM, 1.00 equiv), TCEP HCl (22.8 µL, 1.00 mM, 1.00 equiv) and maleimide **L1** in DMF (22.8 µL, 10.0 mM, 6.00 equiv). SEC chromatography afforded the conjugate **C28** (1.0 mL, 4.9 µM, 22%).

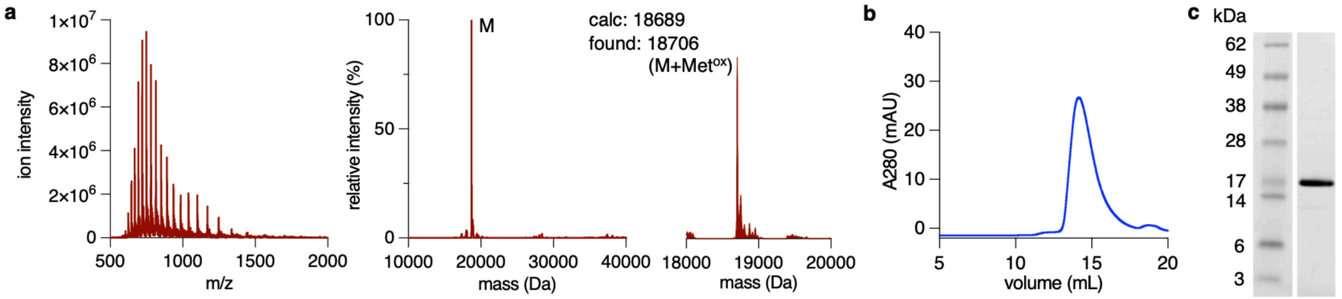

Supporting Figure S62. a, Combined ion series and deconvoluted mass spectra of **C28**. **b**, Chromatogram of SEC purification of **C28**. **c**, SDS-PAGE gel of purified **C28** stained with Coomassie Blue.

DARPin PNA Conjugates

Human CD40 DARPin K4C A144C–P4 staple (C21)

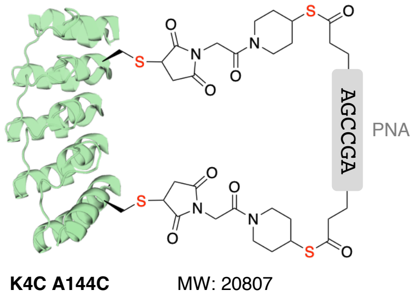

Conjugate **C21** was prepared following the general procedure for stapling of double cysteine proteins with bis-maleimide PNAs by combining protein **K4C A144C** (300 µL, 111 µM, 1.00 equiv) and bis-maleimide–PNA **P4** in PBS–DMF (4:1, v/v) (300 µL, 111 µM, 1.00 equiv) batch-wise in PBS (400 µL). SEC chromatography afforded the conjugate **C21** (1.5 mL, 2.7 µM, 12%).

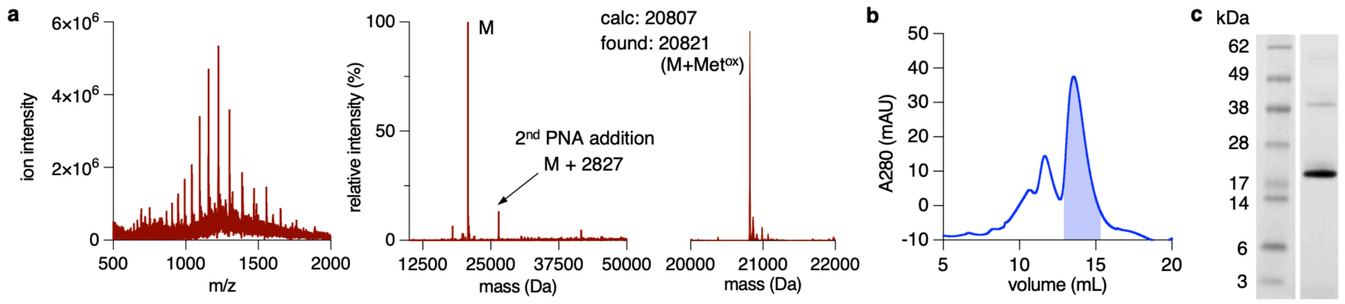

Supporting Figure S63. **a**, Combined ion series and deconvoluted mass spectra of **C21**. **b**, Chromatogram of SEC purification of **C21**. **c**, SDS-PAGE gel of purified **C21** stained with Coomassie Blue.

Human CD40 DARPin Q8C K135C–P4 staple (C22)

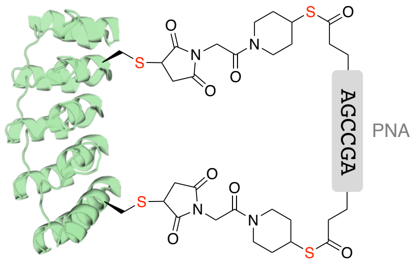

Conjugate **C22** was prepared following the general procedure for stapling of double cysteine proteins with bis-maleimide PNAs by combining protein **Q8C K135C** (300 µL, 95 µM, 1.00 equiv) and bis-maleimide–PNA **P4** in PBS–DMF (4:1, v/v) (300 µL, 95 µM, 1.00 equiv) batch wise in PBS (400 µL). SEC chromatography afforded the conjugate **C22** (1.5 mL, 2.2 µM, 12%).

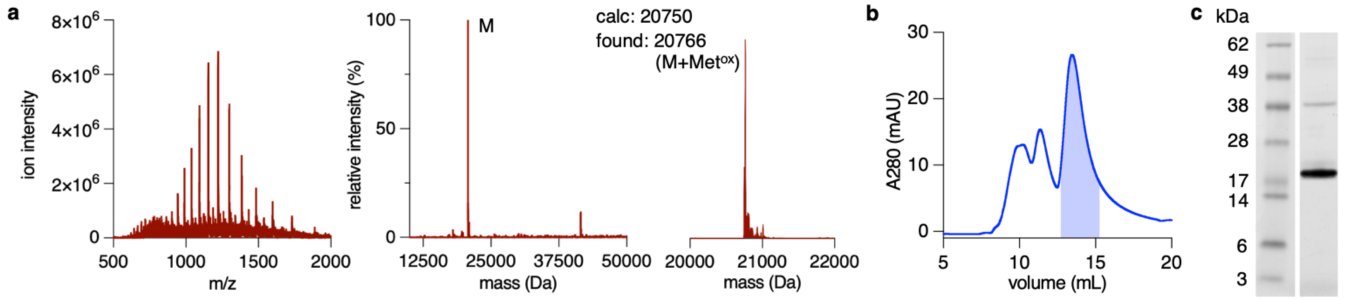

Supporting Figure S64. **a**, Combined ion series and deconvoluted mass spectra of **C22**. **b**, Chromatogram of SEC purification of **C22**. **c**, SDS-PAGE gel of purified **C22** stained with Coomassie Blue.

Human CD40 DARPin R11C K135C–P4 staple (C23)

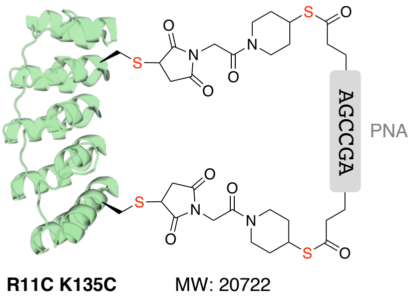

Conjugate **C23** was prepared following the general procedure for stapling of double cysteine proteins with bis-maleimide PNAs by combining protein **R11C K135C** (300 µL, 104 µM, 1.00 equiv) and bis-maleimide–PNA **P4** in PBS–DMF (4:1, v/v) (300 µL, 104 µM, 1.00 equiv) batch-wise in PBS (400 µL). SEC chromatography afforded the conjugate **C23** (1.5 mL, 2.8 µM, 13%).

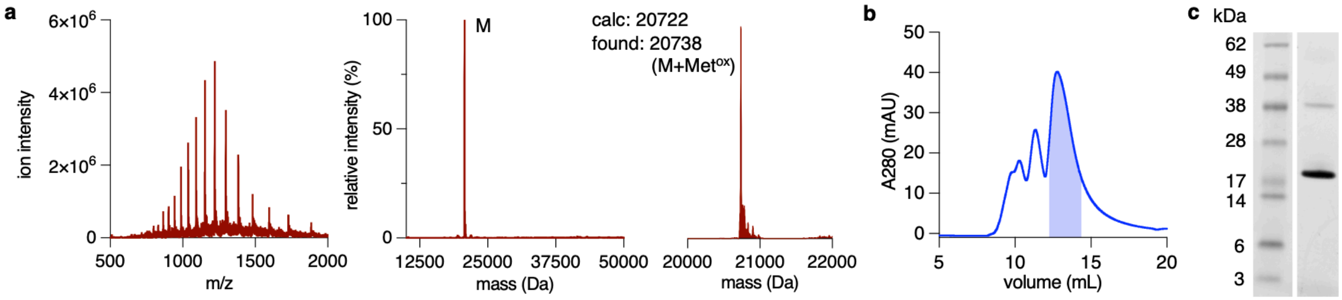

Supporting Figure S65. a, Combined ion series and deconvoluted mass spectra of **C23**. **b**, Chromatogram of SEC purification of **C23**. **c**, SDS-PAGE gel of purified **C23** stained with Coomassie Blue.

Human CD40 DARPin A12C S133C–P4 staple (C24)

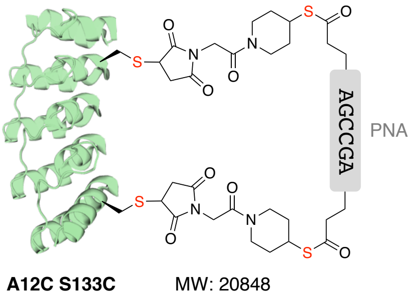

Conjugate **C24** was prepared following the general procedure for stapling of double cysteine proteins with bis-maleimide PNAs by combining protein **A12C S133C** (300 µL, 91 µM, 1.00 equiv) and bis-maleimide–PNA **P4** in PBS–DMF (4:1, v/v) (300 µL, 91 µM, 1.00 equiv) batch wise in PBS (400 µL). SEC chromatography afforded the conjugate **C24** (1.5 mL, 2.5 µM, 14%).

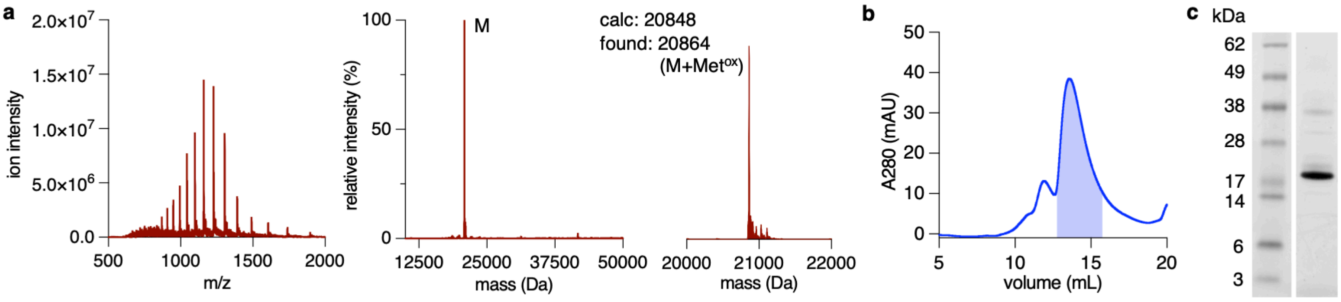

Supporting Figure S66. **a**, Combined ion series and deconvoluted mass spectra of **C24**. **b**, Chromatogram of SEC purification of **C24**. **c**, SDS-PAGE gel of purified **C24** stained with Coomassie Blue.

Fluorescent Conjugates for Serum Studies

Neo-2/15 K33C–maleimide-FAM (SC1)

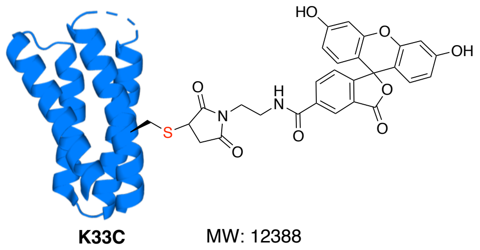

Conjugate **SC1** was prepared following the general procedure for cysteine modification with maleimides using protein **K33C** (200 µL, 93 µM, 1.00 equiv), TCEP HCl (9.3 µL, 1.00 mM, 0.50 equiv) and maleimide-fluorescein in DMF (18.6 µL, 10.0 mM, 2.00 equiv). SEC chromatography afforded the conjugate **SC1** (0.8 mL, 6.0 µM, 26%).

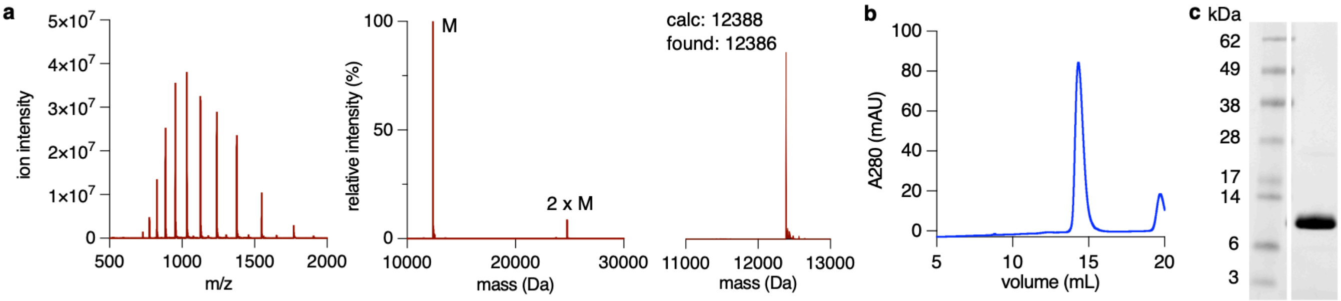

Supporting Figure S67. **a**, Combined ion series and deconvoluted mass spectra of **SC1**. **b**, Chromatogram of SEC purification of **SC1**. **c**, SDS-PAGE gel of purified **SC1** stained with Coomassie Blue.

Neo-2/15 K33C–thioester-FITC (SC2)

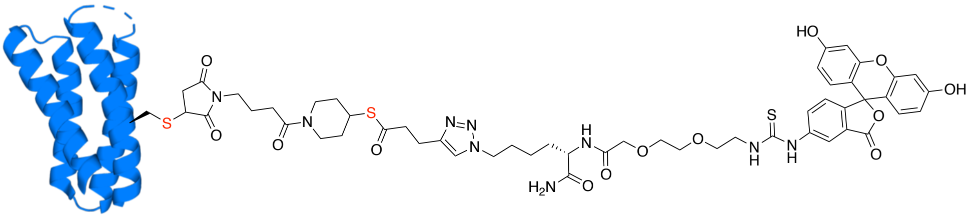

K33C MW: 12958

Conjugate **SC2** was prepared following the general procedure for cysteine modification with maleimides using protein **K33C** (200 µL, 93 µM, 1.00 equiv), TCEP HCl (9.3 µL, 1.00 mM, 0.50 equiv) and maleimide **SP1** in DMF (18.6 µL, 10.0 mM, 2.00 equiv). SEC chromatography afforded the conjugate **SC2** (1.0 mL, 2.0 µM, 11%).

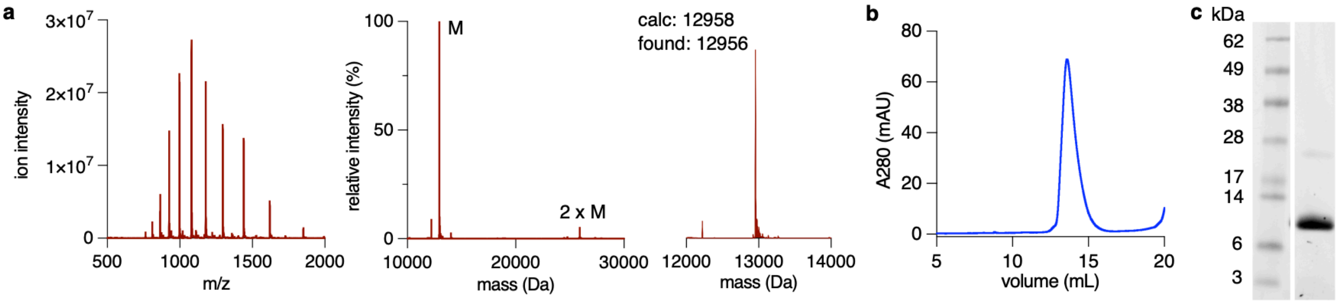

Supporting Figure S68. a, Combined ion series and deconvoluted mass spectra of **SC2**. **b**, Chromatogram of SEC purification of **SC2**. **c**, SDS-PAGE gel of purified **SC2** stained with Coomassie Blue.

Neo2/15 K33C–P2–TAMRA (C5)

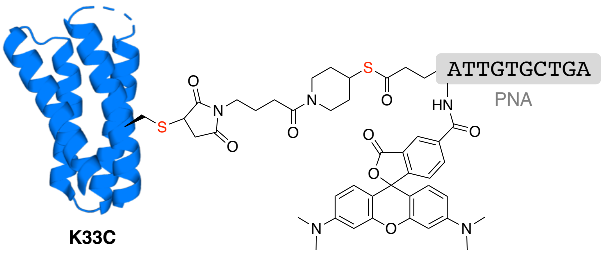

K33C

MW: 16190

Conjugate **C5** was prepared following the general procedure for cysteine modification with maleimides using protein **K33C** (200 µL, 104 µM, 1.00 equiv), TCEP HCl (4.70 µL, 1.00 mM, 0.25 equiv) and maleimide–PNA **SP2** in water (19.0 µL, 1.85 mM, 2.00 equiv). SEC chromatography afforded the conjugate **C5** (1.0 mL, 3.8 µM, 20%).

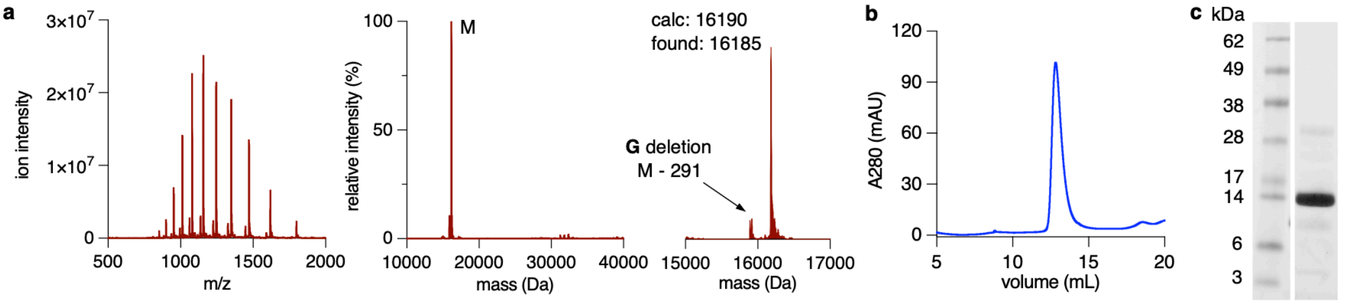

Supporting Figure S69. **a**, Combined ion series and deconvoluted mass spectra of **C5**. **b**, Chromatogram of SEC purification of **C5**. **c**, SDS-PAGE gel of purified **C5** stained with Coomassie Blue.

Superfolder GFP S147C–P4 dimer (C20)

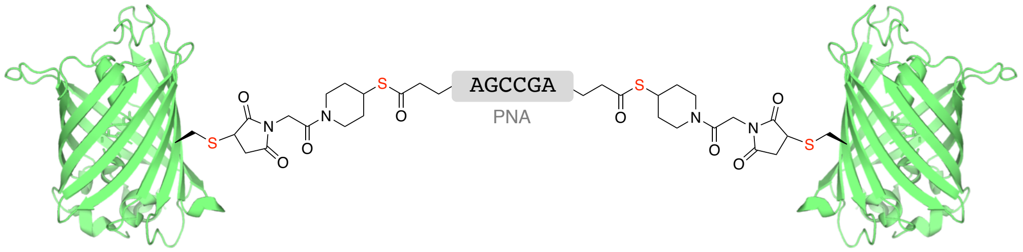

Conjugate **C20** was prepared following the general procedure for homo-dimerization of single cysteine proteins with bis-maleimide PNAs using protein **sfGFP S147C** (300 µL, 77 µM, 1.00 equiv) and bis-maleimide–PNA **P4** in PBS–DMF (4:1, v/v) (116 µL, 100 µM, 0.50 equiv). SEC chromatography afforded the conjugate **C20** (1.0 mL, 3.3 µM, 14%).

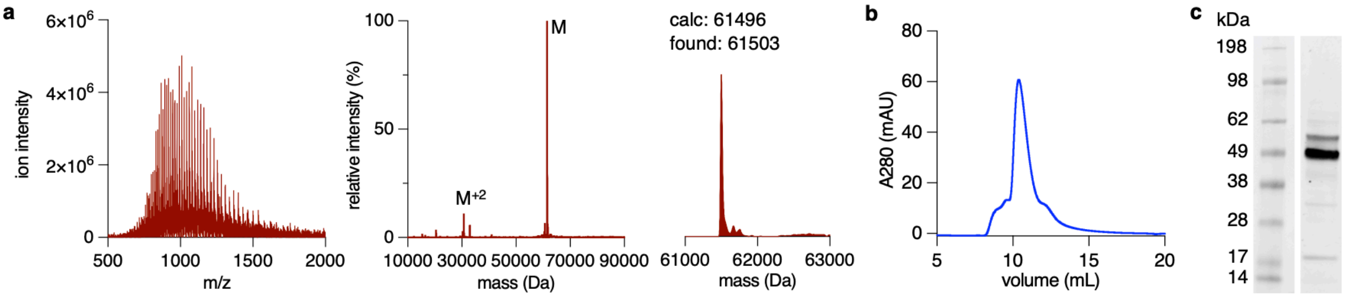

Supporting Figure S70. **a**, Combined ion series and deconvoluted mass spectra of **C20**. **b**, Chromatogram of SEC purification of **C20**. **c**, SDS-PAGE gel of purified **C20** stained with Coomassie Blue.

Binding and Stability Assays of Bioconjugates

Biolayer Interferometry (BLI) Measurements

BLI measurements were performed on an Octet® K2 system (Pall ForteBio) using Octet® streptavidin-coated (SA) biosensors in the following buffered solution (pH 7.4): 10 mM HEPES, 150 mM NaCl, 3.0 mM EDTA, 0.05% Tween-20, 10 µM bovine serum albumin (BSA). Biotinylated proteins were dissolved following the manufacturer's instructions at a concentration of 200 µg/mL and loading onto SA biosensor was performed at 1.0 µg/mL. Binding experiments were conducted at 30°C and under 1000 rpm shaking using the following steps: 1. SA biosensor regeneration in 10 mM glycine buffer (pH 3.0) and neutralization; 2. equilibration (t = 60 s); 3. loading (t = 100 s); 4. washing step (t = 60 s); 5. baseline (t = 60 s); 6. association (t = 200 s); 7. dissociation (t = 350 s). A reference SA biosensor (no biotinylated ligand) was used as background and smoothing of curves was applied using Savitzky–Golay filter and sensorgrams were normalized to the average of the baseline step. Fitting was performed using the manufacturer's program.

Serum Stability Determination

Serum stability experiments were performed with human serum (cat. H4522) purchased from Sigma Aldrich. Fluorescently-labeled conjugates in PBS (25 µL, 2.0 µM) were combined with freshly-thawed human serum (25 µL) and incubated at 37 °C without shaking. For timepoints, 3.0 µL of the solutions were taken, frozen with liquid nitrogen and stored at -80 °C. For the analysis by SDS-PAGE, all aliquots were thawed, diluted with 3.0 µL water and loaded on the gels with 2.0 µL LDS loading buffer. SDS-PAGE gels were analyzed using the fluorescein or Cy3 channel on a Bio-Rad ChemiDoc MP system with 2.0 s manual exposure time. Band intensities were analyzed using the software Image Lab version 6.1.0 and half-life times estimated by plotting the loss of intensity over time using GraphPad Prism 9.3.1.

Templated Removal of PNA Masking Groups

Templated removal reactions were performed at in PBS or PBS–human serum (1:1) at room temperature or 37 °C. To a solution of PNA conjugate in PBS (2–9 µM) was added TCEP HCl (stock solution: 1.0 mM, 1.0–3.0 equiv) followed by a solution of Key-PNA in water (stock solution: 0.5 m–1.0 mM, 1.0–3.0 equiv). For control experiments, only TCEP HCl was added to the PNA conjugate. The removal reaction mixture was mixed using a pipette and allowed to proceed without shaking. For timepoints, 10 µL of the solution were taken, frozen with liquid nitrogen and stored at -80 °C. For timepoints with *N*-methyl maleimide (NMM) quenching, 10 µL of the solution were taken and 1.0 µL of a solution of NMM in MeCN (0.3 mM) was added. Templated removal was analyzed by LC-MS or SDS-PAGE.

Ex vivo T cell STAT5 Signaling Assay

Freshly isolated BALB/c splenocytes were stained with APC anti-CD4 (Clone GK1.5, eBioscience) and CD4 T cells were separated by magnetic-associated cell sorting (MACS) using Anti-APC MojoSort magnetic beads (Biolegend) in a magnetic stand. Alternatively, HH cells were used. 5×10^4 cells were stimulated *ex vivo* with different concentrations of Neo-2/15 **WT**, PNA-bridged dimer **C19**, and **C19** treated with Key-PNA **P6** for 30 min at 37°C in complete RPMI [RPMI supplemented with 10% HI fetal bovine serum (FBS), GlutaMAX, HEPES, Non-essential amino acids (NEAA), penicillin-streptomycin, and sodium pyruvate]. For pSTAT5 assays, cells were fixed for 15 minutes at 37ºC, permeabilised with pre-chilled True-Phos Perm Buffer (Biolegend) for 1 h and stained with PE Mouse anti-Stat5 (Clone 47/Stat5(pY694), BD Phosflow). Flow cytometric data were acquired on BD LSRFortessa with a High Throughput Sampler using BD FACSDiva software, and analysed using FlowJo Software (version 10.10.0).

NMR Spectra of Synthesized Compounds

tert-Butyl 4-(pent-4-ynoylthio)piperidine-1-carboxylate (S2)

^{1}H NMR (400 MHz, 298K)
$CDCl_3$

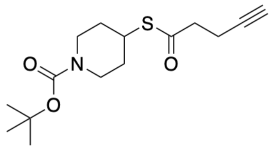

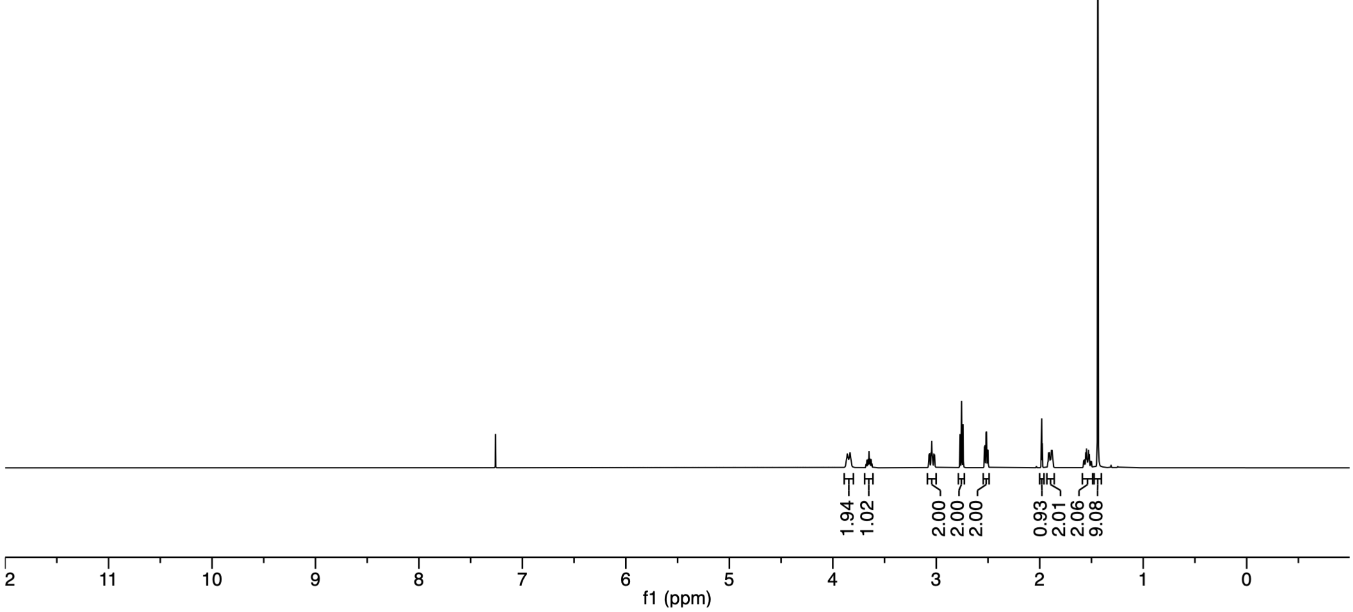

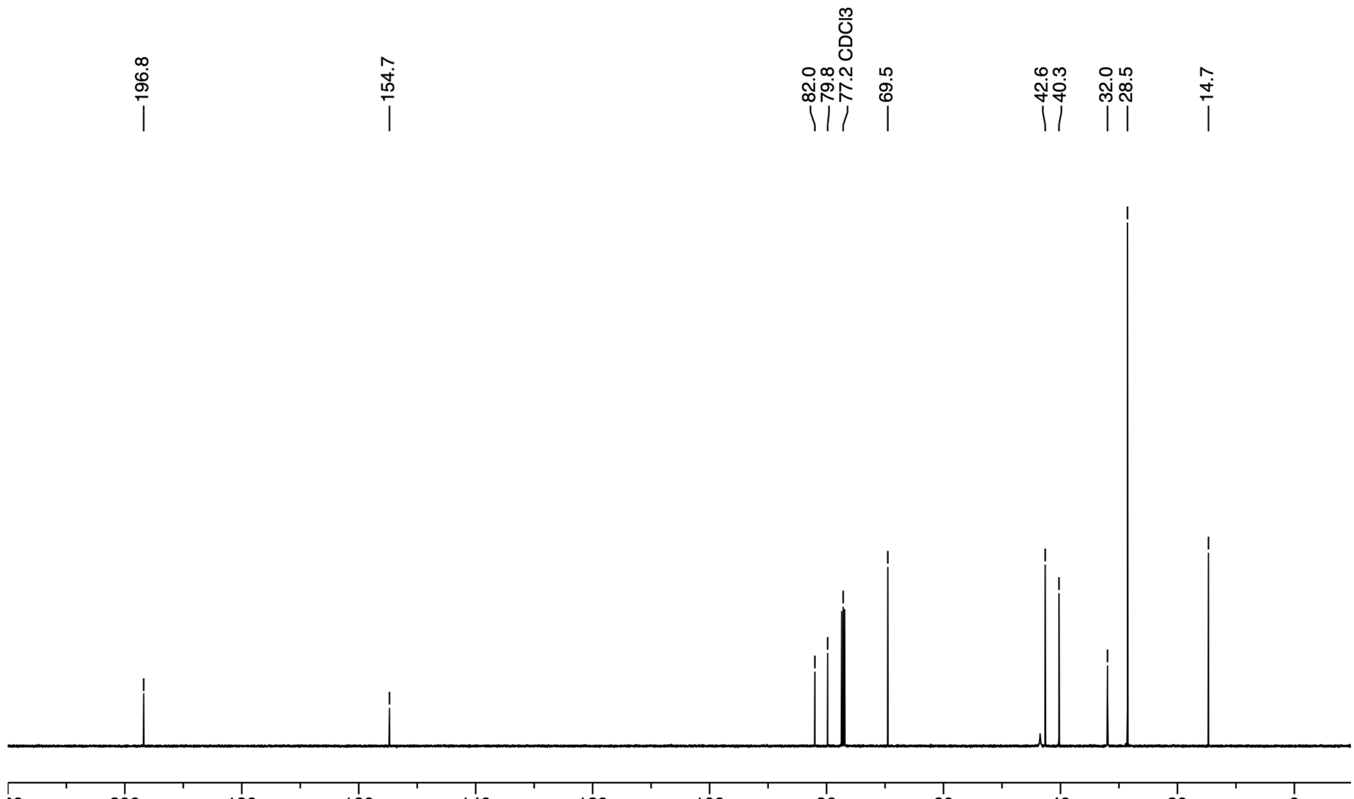

Thioester linker 1 (L1)

^{1}H NMR (400 MHz, 298K)
$CDCl_3$

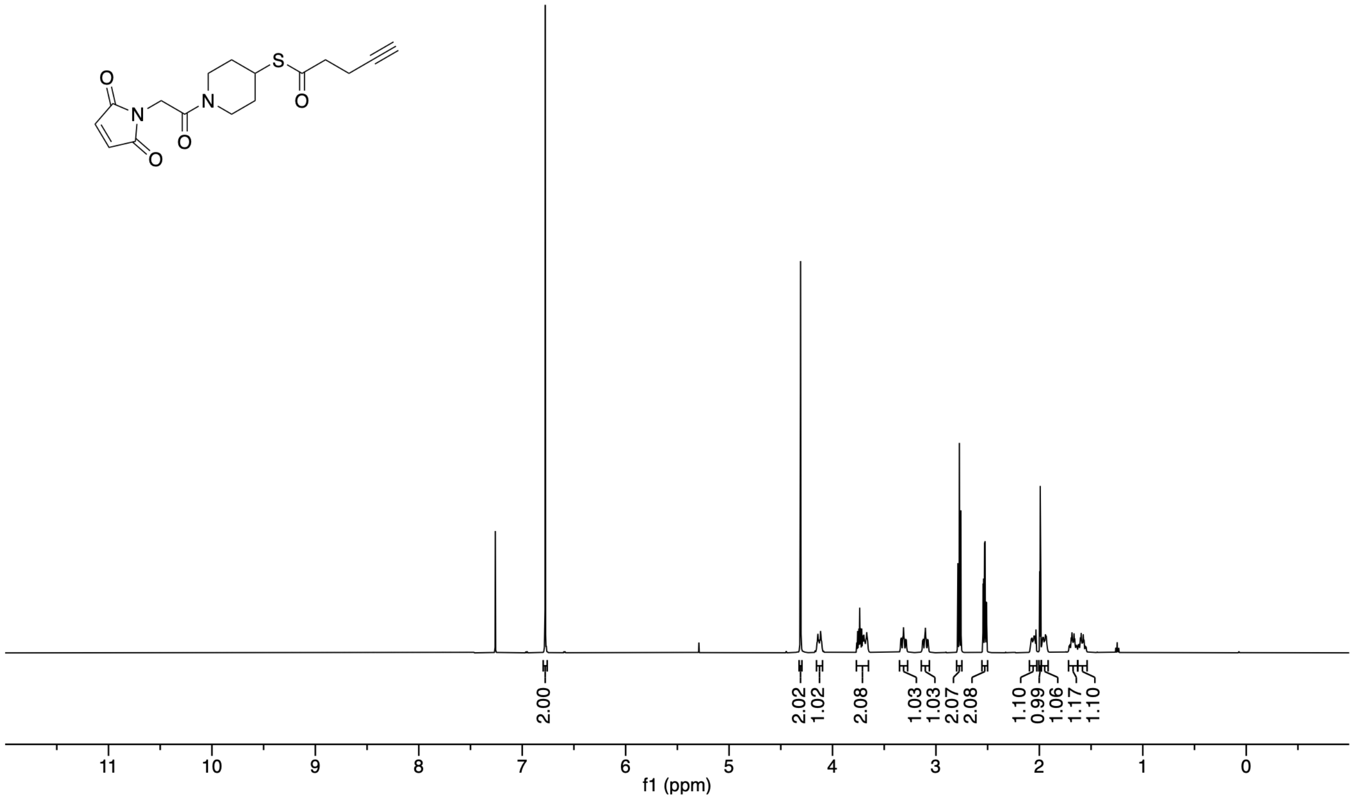

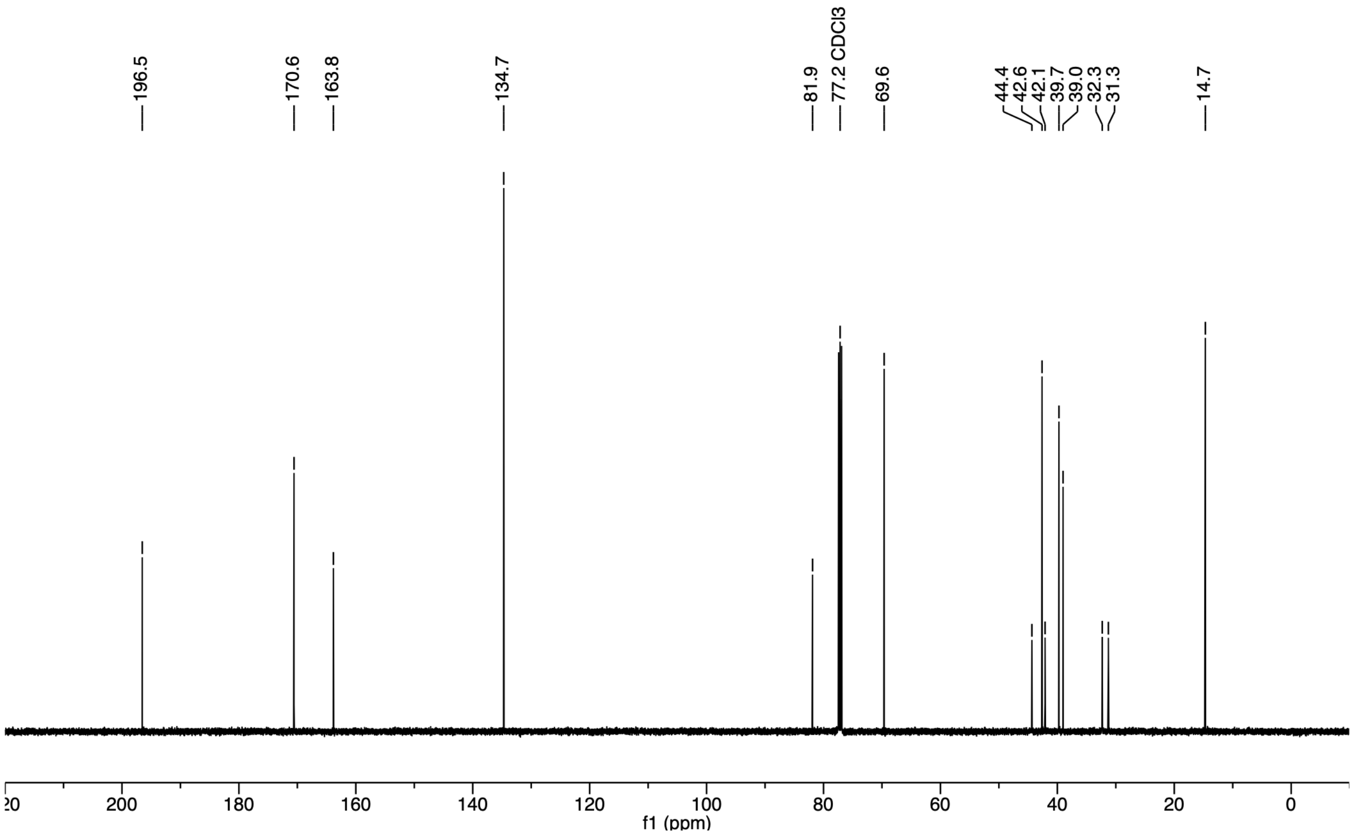

Thioester linker 2 (L2)

^{1}H NMR (400 MHz, 298K)
$CDCl_3$

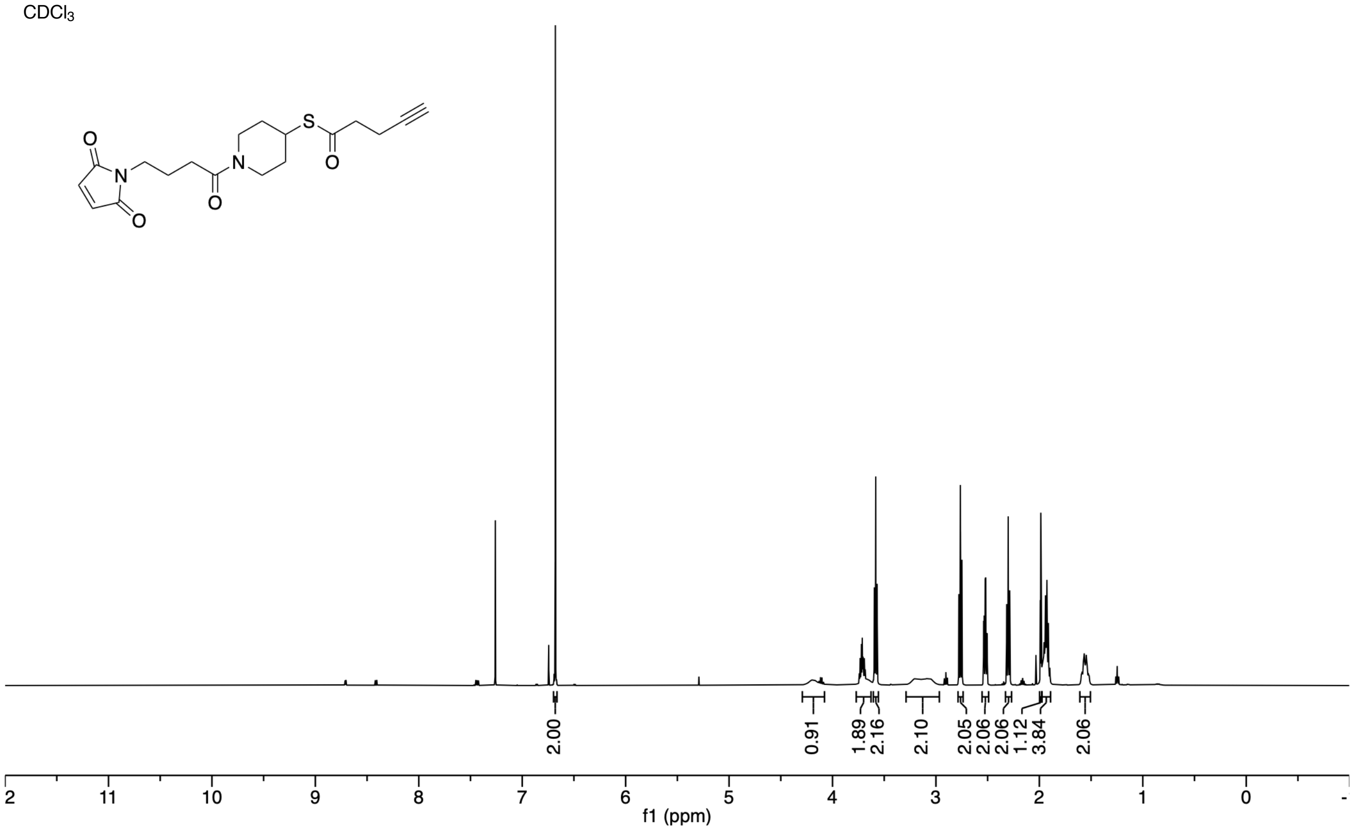

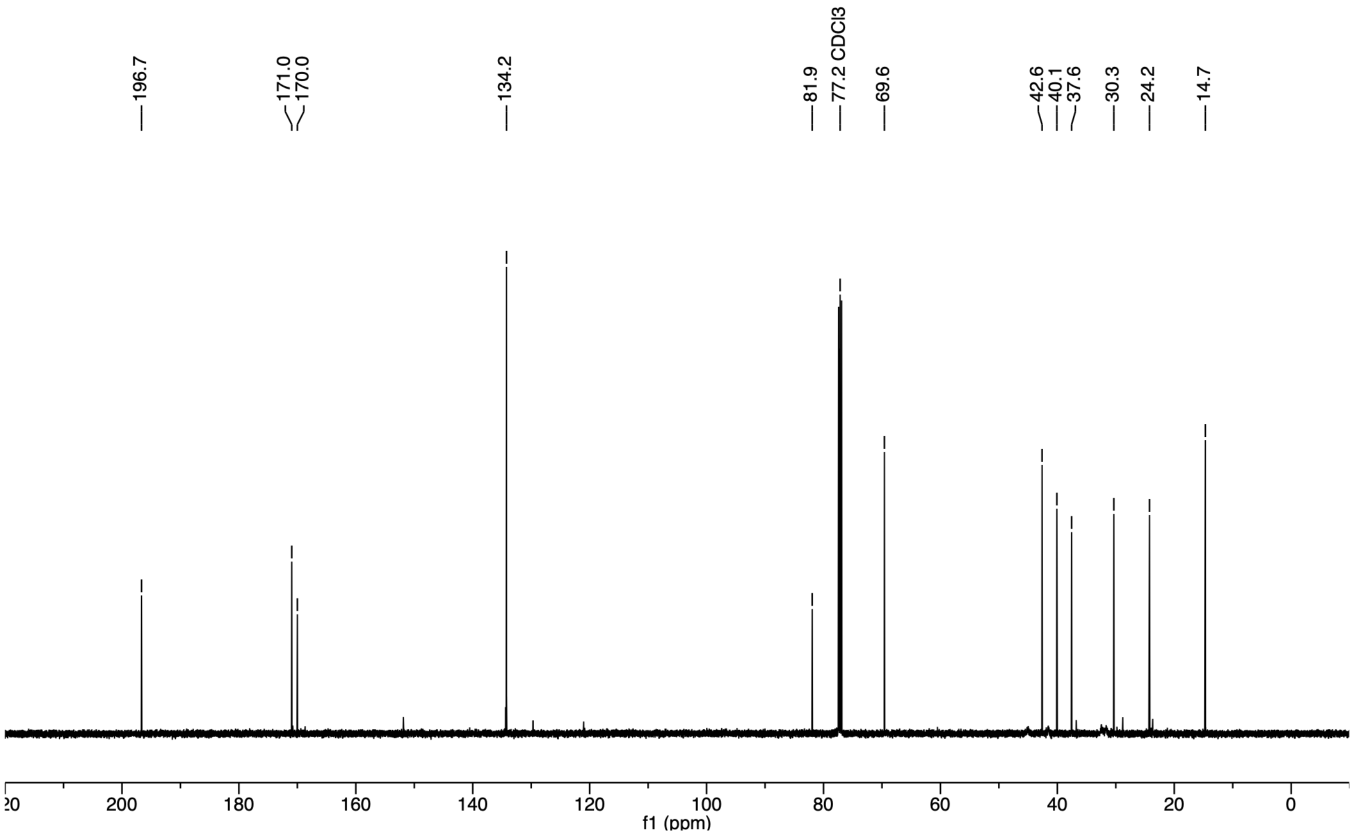

Uncropped SDS-PAGE gels

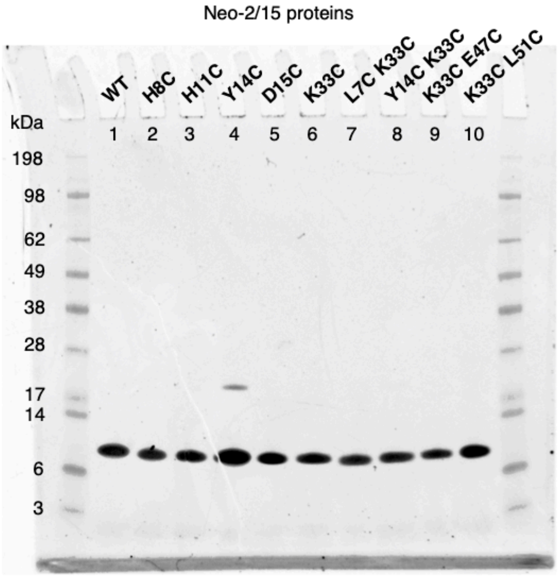

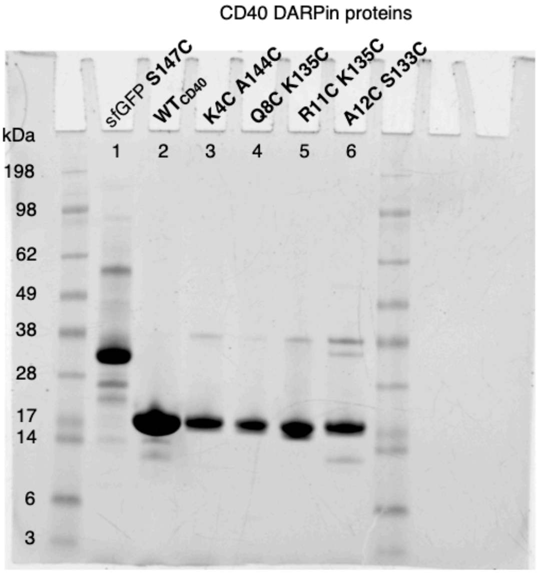

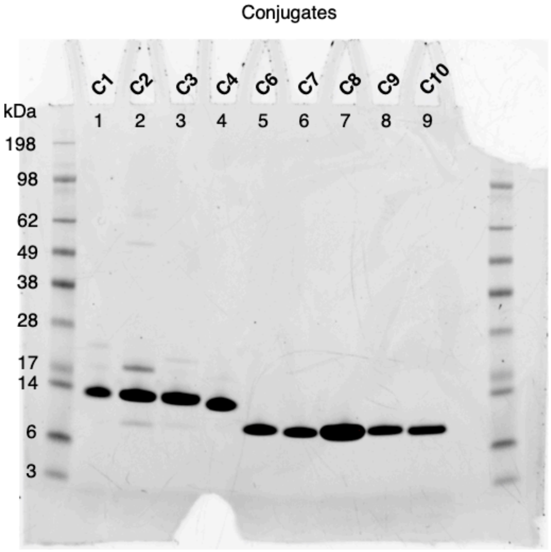

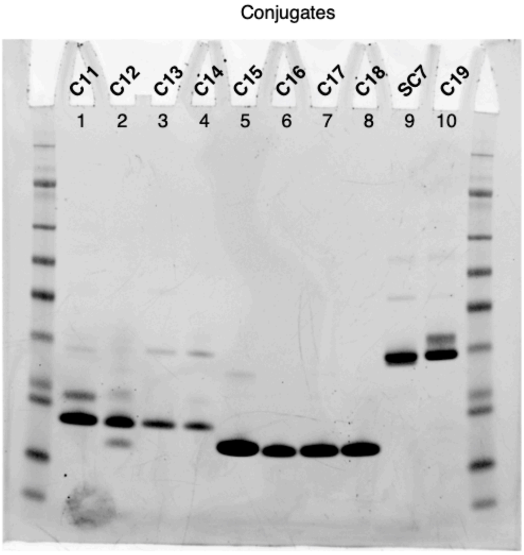

Supporting Figure S71. Uncropped SDS-PAGE gels.

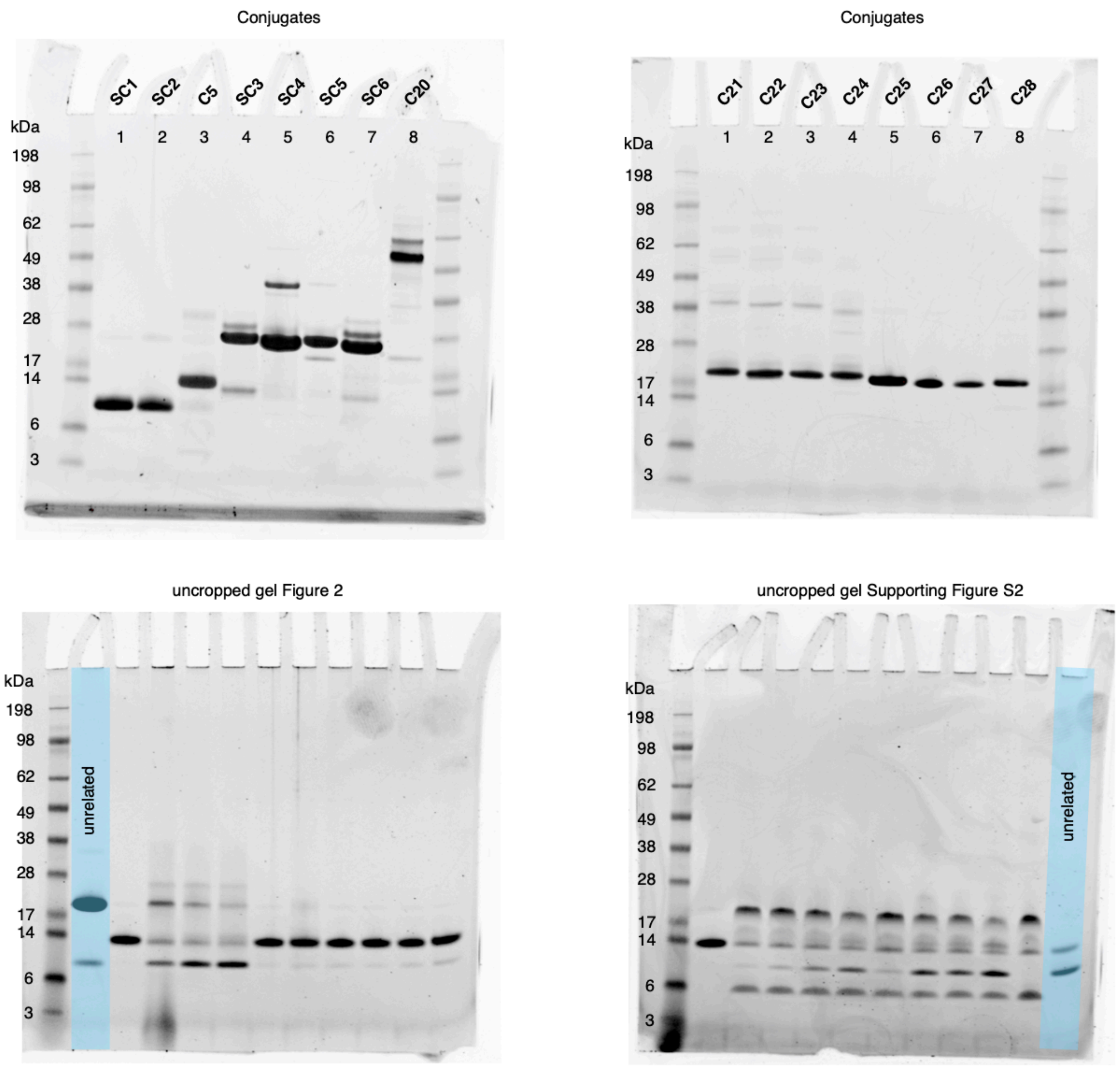

Supporting Figure S72. Uncropped SDS-PAGE gels.

uncropped gel Supporting Figure S3

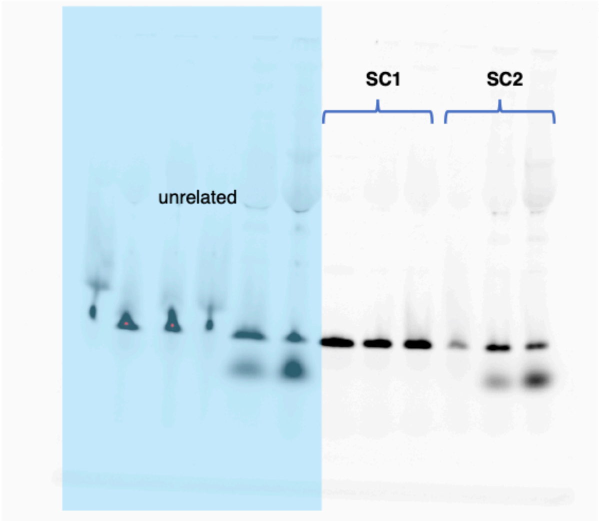

fluorescence (fluorescein channel)

uncropped gel Supporting Figure S3

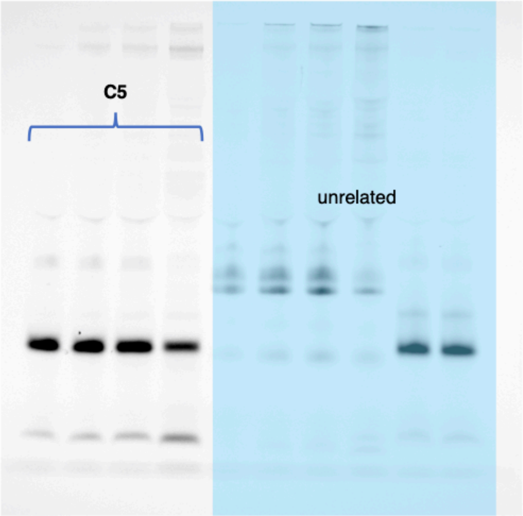

fluorescence (Cy3 channel)

uncropped gel Supporting Figure S3

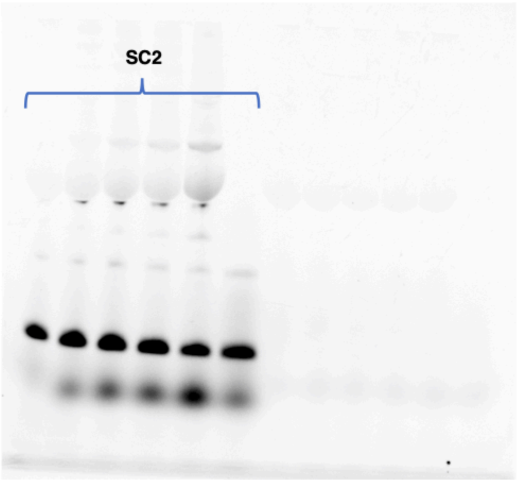

fluorescence (fluorescein channel)

uncropped gel Supporting Figure S3

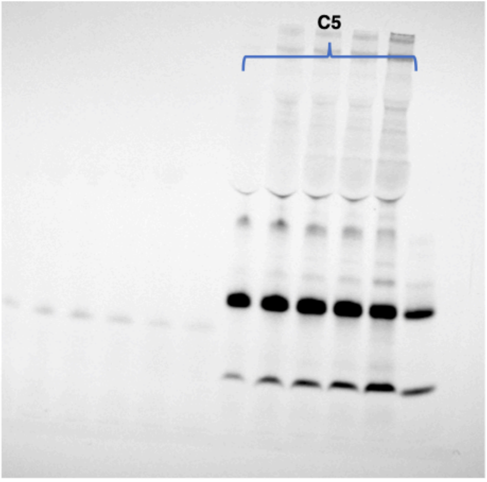

fluorescence (Cy3 channel)

Supporting Figure S73. Uncropped SDS-PAGE gels.

uncropped gel Figure 4 and Supporting Figure S7

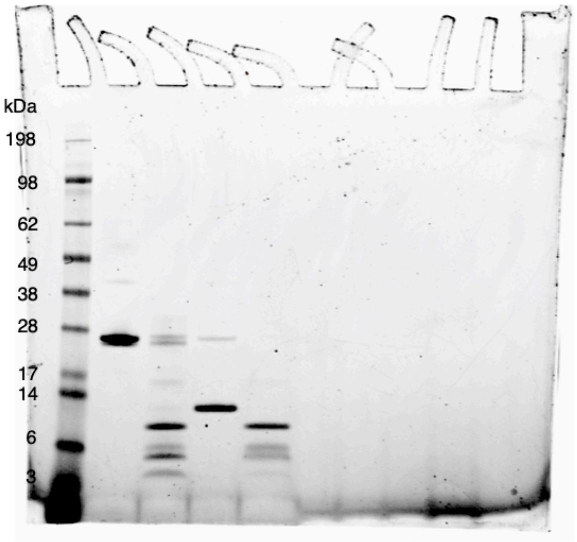

uncropped gel Supporting Figure S8

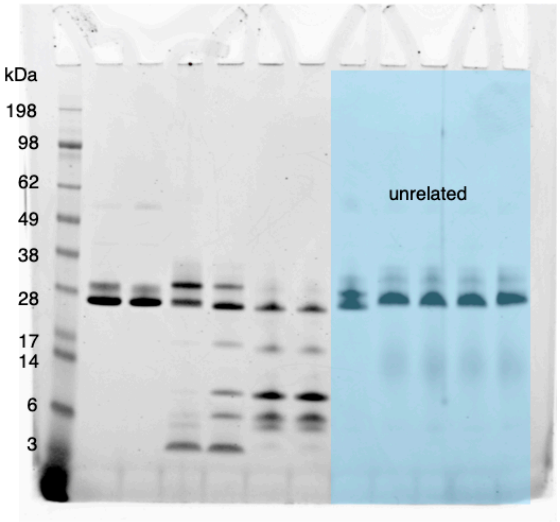

uncropped gel Supporting Figure S9

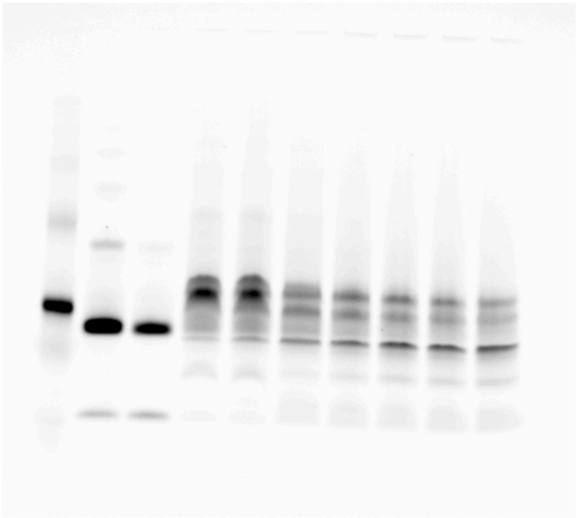

fluorescence (Cy3 channel)

uncropped gel Supporting Figure S9

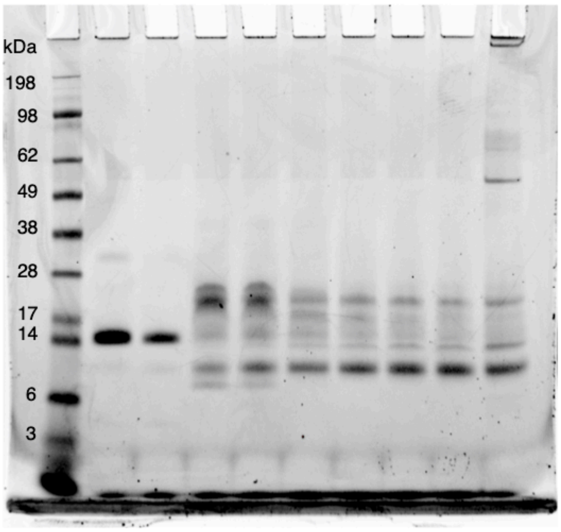

Supporting Figure S74. Uncropped SDS-PAGE gels.

uncropped gel Figure 5 and Supporting Figure S9

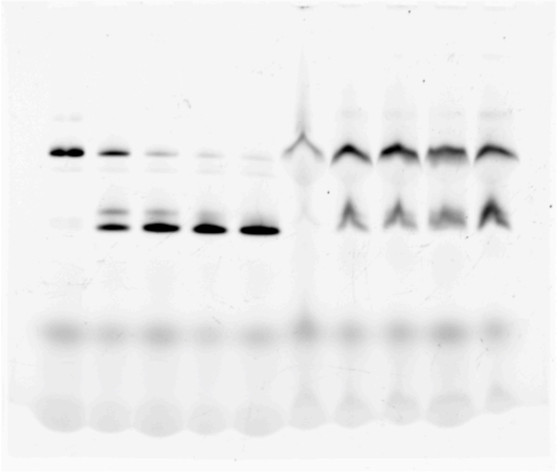

fluorescence (fluorescein channel)

uncropped gel Figure 6

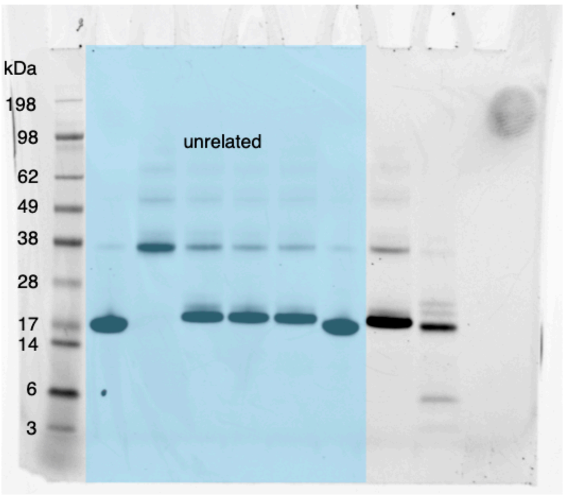

uncropped gel Supporting Figure S6

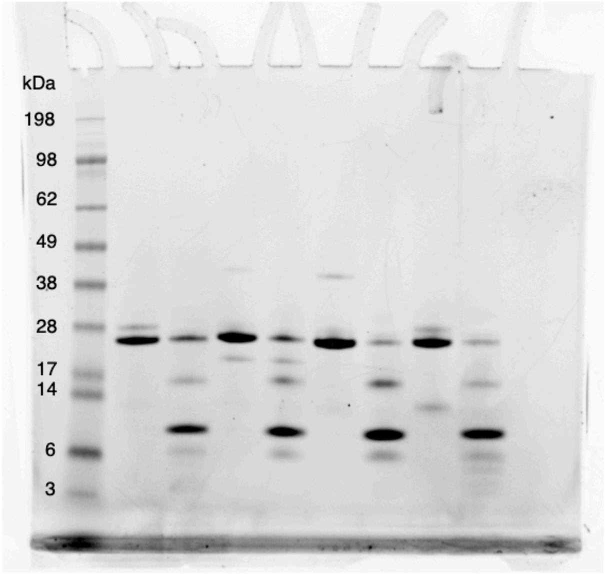

Supporting Figure S75. Uncropped SDS-PAGE gels.

References

[1] D.-A. Silva, S. Yu, U. Y. Ulge, J. B. Spangler, K. M. Jude, C. Labão-Almeida, L. R. Ali, A. Quijano-Rubio, M. Ruterbusch, I. Leung, T. Biary, S. J. Crowley, E. Marcos, C. D. Walkey, B. D. Weitzner, F. Pardo-Avila, J. Castellanos, L. Carter, L. Stewart, S. R. Riddell, M. Pepper, G. J. L. Bernardes, M. Dougan, K. C. Garcia, D. Baker, *Nature* **2019**, *565*, 186.

[2] J. I. Yeh, E. Pohl, D. Truan, W. He, G. M. Sheldrick, S. Du, C. Achim, *Chem. Eur. J.* **2010**, *16*, 11867.

[3] N. Rigamonti, N. Veitonmäki, C. Domke, S. Barsin, S. Jetzer, O. Abdelmotaleb, R. Bessey, T. Lekishvili, F. Malvezzi, M. Gachechiladze, M. Behe, V. Levitsky, P. A. Trail, *Cancer Immunol. Res.* **2022**, *10*, 626.
